# Current Status and Issues Regarding Pre-processing of fNIRS Neuroimaging Data: An Investigation of Diverse Signal Filtering Methods Within a General Linear Model Framework

**DOI:** 10.3389/fnhum.2018.00505

**Published:** 2019-01-11

**Authors:** Paola Pinti, Felix Scholkmann, Antonia Hamilton, Paul Burgess, Ilias Tachtsidis

**Affiliations:** ^1^Department of Medical Physics and Biomedical Engineering, University College London, London, United Kingdom; ^2^Institute of Cognitive Neuroscience, University College London, London, United Kingdom; ^3^Department of Neonatology, Biomedical Optics Research Laboratory, University Hospital Zurich, University of Zurich, Zurich, Switzerland

**Keywords:** functional near infrared spectroscopy, digital filter, general linear model, pre-processing standardization, functional data analysis, pre-processing guidelines

## Abstract

Functional near-infrared spectroscopy (fNIRS) research articles show a large heterogeneity in the analysis approaches and pre-processing procedures. Additionally, there is often a lack of a complete description of the methods applied, necessary for study replication or for results comparison. The aims of this paper were (i) to review and investigate which information is generally included in published fNIRS papers, and (ii) to define a signal pre-processing procedure to set a common ground for standardization guidelines. To this goal, we have reviewed 110 fNIRS articles published in 2016 in the field of cognitive neuroscience, and performed a simulation analysis with synthetic fNIRS data to optimize the signal filtering step before applying the GLM method for statistical inference. Our results highlight the fact that many papers lack important information, and there is a large variability in the filtering methods used. Our simulations demonstrated that the optimal approach to remove noise and recover the hemodynamic response from fNIRS data in a GLM framework is to use a 1000th order band-pass Finite Impulse Response filter. Based on these results, we give preliminary recommendations as to the first step toward improving the analysis of fNIRS data and dissemination of the results.

## Introduction

**Figure 1:**
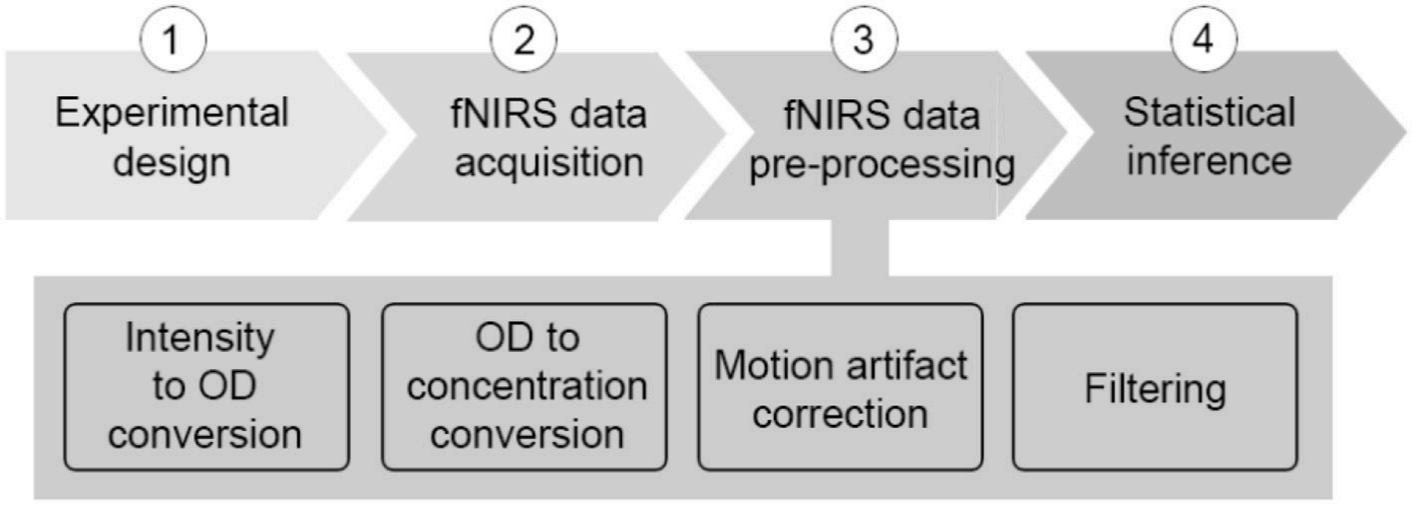
Typical neuroscience experiment pipeline with fNIRS.

The last few years have seen a rapid (almost exponential) growth in the number of functional neuroimaging studies performed and published with functional near-infrared spectroscopy (fNIRS) (Yücel et al., 2017). fNIRS has provided neuroscientists and clinicians with a novel and invaluable tool to study and monitor tissue oxygenation changes in the brain non-invasively. Based on neurovascular coupling, fNIRS measures the brain tissue concentration changes in oxyhemoglobin (HbO_2_) and deoxyhemoglobin (HbR) associated with an increased metabolic demand of the brain during neuronal activity, and an increased tissue perfusion (Scholkmann et al., 2014). To date, one of the major fields of application of fNIRS is cognitive neuroscience, where the mechanisms underlying brain functioning are typically investigated by monitoring the task or stimulus-evoked changes in the brain during the execution of cognitive tasks (see Pinti et al., 2018 for review). fNIRS is well-suited to this application since it allows the study of cognition with very few physical constraints, allowing brain monitoring in a wide range of cognitive tasks, e.g., those including bodily movements, and in a variety of populations, e.g., infants, healthy adults, clinical patients. A typical sequence of steps performed in a neuroscience with fNIRS is shown in Figure 1, usually comprising 4 main steps.

*Step 1:* The first step is the design and implementation of the experimental protocol. Block or event-related designs are usually employed, in which the stimuli are presented several times to increase the statistical power, and experimental task periods are typically interspersed with contrast conditions or stimuli (or in some cases rest periods) to better assess the presence of hemodynamic responses. fNIRS data are then collected during the execution of the designed experiment. A mixed block/event-related design can be also employed (Petersen and Dubis, 2012).

*Step 2:* The data acquisition step comprises the placement of a certain number of light sources and detectors (i.e., “optodes”) on the participants' head by means of fiber optics, and at a light source-detector distance of 3 cm in case of studies with adults. The raw fNIRS signal measured by the detector, e.g., the raw light intensity signal, originates from the tissue volume located below the source and detector having a maximal depth a bit less than half the source-detector distance [i.e., this is called “channel” (Patil et al., 2011)]. The number of channels and the sampling frequency of the acquisition depend on the particular fNIRS instrument used.

*Step 3:* During the pre-processing phase, the raw intensity data are usually visually inspected to assess signals' quality (e.g., the presence of large motion artifacts, and of heart beat oscillations indicating a good optical coupling between the optodes and the scalp). Intensity time-series are converted into changes in attenuation (or optical density, ΔOD) and then into concentration changes of HbO_2_ and HbR (ΔHbO_2_ and ΔHbR), usually by means of the modified Beer-Lambert law (Delpy et al., 1988). In order to extract useful information from fNIRS data, any source of variability in the ΔHbO_2_ and ΔHbR signals not related to the task-evoked hemodynamic activity needs to be removed, or at least minimized. For a review on the structures and the statistical properties of the noises that are often present in fNIRS data, we advise the reader to see the publication of Huppert (2016). One typically experienced source of noise is that due to head movements. In fact, although fNIRS is more robust to motion artifacts than other modalities [e.g., functional magnetic resonance imaging (fMRI), electroencephalography/magnetoencephalography (EEG/MEG)], signals can be corrupted by head movements, causing fast spikes or shifts from the baseline values. The most common practice to deal with these motion errors is to include the identification and correction of such artifacts as a step in the signal pre-processing stream. Several techniques have been proposed so far and have been reviewed elsewhere (Brigadoi et al., 2014). In addition, fNIRS data are also contaminated by physiological noises not directly related to cortical brain activity that can deteriorate the Signal-to-Noise Ratio (SNR), and mask and/or mimic the presence of brain hemodynamic responses (Tachtsidis and Scholkmann, 2016). The origin of these components and the methods developed so far to reduce their impact on the estimation of brain activity by fNIRS have been reviewed by Scholkmann et al. (2014). Briefly, such physiological changes contribute a large amount of variance to the fNIRS signals and can be elicited both (i) by the execution of the cognitive task, and (ii) spontaneously. In the first case, the execution of particularly complex or stressful tasks can affect the psychophysiological state of the participant, resulting in changes in heart rate, breathing rate, blood pressure, carbon dioxide (CO_2_) concentration of the blood and autonomic regulatory activity happening both at the intra- and extra-cerebral levels (Rowley et al., 2006; Kirilina et al., 2012; Scholkmann et al., 2013; Holper et al., 2014; Tachtsidis and Scholkmann, 2016); the second case refers to the spontaneous hemodynamic oscillations related to physiological vasomotor regulations and breathing-related fluctuations (Tachtsidis et al., 2004; Tong et al., 2012). These spontaneous components are characterized by signals at specific frequencies associated with the heart rate (~1 Hz), breathing rate (~0.3 Hz), Mayer waves (~0.1 Hz), and very low frequency (< 0.04, VLF) oscillations. One of the most common and more straightforward approaches used by the scientific community to reduce the impact of these components, is to remove specific frequency bands in fNIRS signals by means of digital filters. Digital filtering is a mathematical procedure applied to discrete time-series to reduce or enhance certain properties of the input signals (e.g., frequency ranges). Filters are divided into three classes: (i) *high-pass filters*, which remove high frequency components above the cut-off frequency; (ii) *low-pass filters*, which remove low frequency components below the cut-off frequency; (iii) *band-pass filters*, which preserve the frequency range between a lower and a higher cut-off frequency. Some research groups apply filters on ΔOD data prior the conversion into concentration changes; others apply the filter on the ΔHbO_2_ and ΔHbR signals. However, in both cases, the frequency range to include needs to be chosen carefully in order to preserve the stimulus frequency and to preserve the task-evoked response.

*Step 4:* Once the data are pre-processed, statistical analyses are performed, and the pre-processed ΔHbO_2_ and ΔHbR signals are used to make inference about task-evoked functional brain activity (see Tak and Ye, 2014 for a review). One of the most common statistical frameworks employed by the community is the General Linear Model (GLM). This approach has more statistical power than other methods commonly used for fNIRS (e.g., block averaging). In fact, the GLM considers the entire fNIRS time series taking advantage of the high temporal resolution of fNIRS. It also provides good flexibility as it allows to test specific hypothesis by comparing combinations of the experimental conditions with different statistical testing approaches (e.g., t-tests, F-tests, ANOVAs, ANCOVAs; Monti, 2011). In addition, it permits the inclusion of other covariates within the model or design matrix [e.g., behavioral performance, head movement, physiological signals, short-separation fNIRS channels (Tachtsidis and Scholkmann, 2016)] to improve the inference accuracy. However, the GLM has the disadvantage that it assumes a specific pre-defined hemodynamic response function, which e.g., to a great extent is still unknown for neonates or might be different across brain regions.

It is important to highlight how the experiment pipeline described above (Figure 1) is not made of stand-alone steps. Each phase influences the other and, more importantly, they influence the outcome of the statistical analyses and the study results. For instance, if the experimental protocol is not carefully designed and, for example, a task block duration of ~10 s is chosen, the task frequency (~0.1 Hz) overlaps with the Mayer wave oscillation, leading to inflated statistics. Additionally, the pre-processing stream has a major impact also on the comparison of results among different studies and research groups, and on study replication, because the statistical analyses depend on the data content. It is therefore extremely important and good practice to always report detailed information about each individual step of the experiment pipeline (Figure 1), from the protocol specification (type of stimuli, structure, durations, presentation software), the device features (model, sampling frequency, wavelengths), signal pre-processing (algorithm to compute ΔHbO_2_ and ΔHbR, motion artifact correction algorithm, filter parameters), to the statistical analyses (hypotheses, statistical test).

Whilst all these procedures are almost standardized for other neuroimaging modalities such as fMRI, this is not the case for fNIRS yet. As recently highlighted by Hocke et al. (2018), fNIRS publications often lack useful information, and there is a huge variability in the analysis procedures and in the way methods are described. For instance, the absence of standardization of input parameters for fNIRS pre-processing and analysis methods can lead to suboptimal papers or irreproducible studies and results. In addition, the authors demonstrated how the use and the combination of different methods (e.g., criteria for identifying noisy channels, motion artifact correction, signals' filtering, etc.) can lead to different results, influencing the neuroscientific conclusions. Another relevant issue is related to the best fNIRS-derived signal to infer functional brain activity, as fNIRS provides measurements of both HbO_2_ and HbR. For example, some papers draw neuroscientific conclusions based only on ΔHbO_2_. But, others report total hemoglobin (ΔHbO_2_ +ΔHbR), and yet others describe both ΔHbO_2_ and ΔHbR. Therefore, there is an urgent need to move toward a standardization of the experimental procedures, right through from the study design phase to the presentation of results. The aim of the current report is to start tackling this standardization issue and to set the ground for the development of toward common guidelines. More precisely, in this work we focus (i) on the filtering step of the pre-processing phase and (ii) on the assessment of the completeness of the information reported in the published research articles. To this end, we first review the papers published in 2016 in the field of functional neuroimaging with fNIRS to analyse information on (i) the latest most used filtering approaches and (ii) the data inclusiveness. Then, we test the identified filter specifications on synthetic fNIRS data generated from 18 subjects resting state data with a superimposed task-related component simulating a block-design experiment, and explore the effect of filters and their application to ΔOD or ΔHbO_2_ and ΔHbR on the outcome of statistical analyses in order to optimize the inference procedure in a GLM-based framework.

## Literature Review

A literature review of fNIRS articles published in the field of cognitive neuroscience was performed as first step with the aim of identifying the most common filtering approaches adopted by the community, and of evaluating the completeness of the information reported in research papers. To this end, we used the PubMed database, plus a manual search from articles, references, and the publication surveys made available by the Society for functional Near Infrared Spectroscopy (http://fnirs.org/publications/nirs-niri-publications/). Articles were selected on the basis of the following criteria:

1. Papers published in 2016, in order to review the most updated and advanced pre-processing approaches as a representative sample of the fNIRS field
2. Original research articles published on peer-reviewed journals. Conference proceedings and review papers were excluded from further analyses
3. Studies conducted on adults, as infants' fNIRS data have different spectral characteristics [e.g., a higher heart rate frequency band (von Siebenthal et al., 1999)] and thus different filtering specifications must be used
4. Papers that included task-evoked functional activation experiments, as fNIRS is by far mostly used for monitoring task-related brain activity in response to cognitive tasks
5. Our analysis included only continuous-wave (CW) fNIRS studies looking at concentration changes of oxy- and deoxy- hemoglobin due to the popularity of CW-fNIRS in current fNIRS research and neuroscience applications.

**Figure 2:**
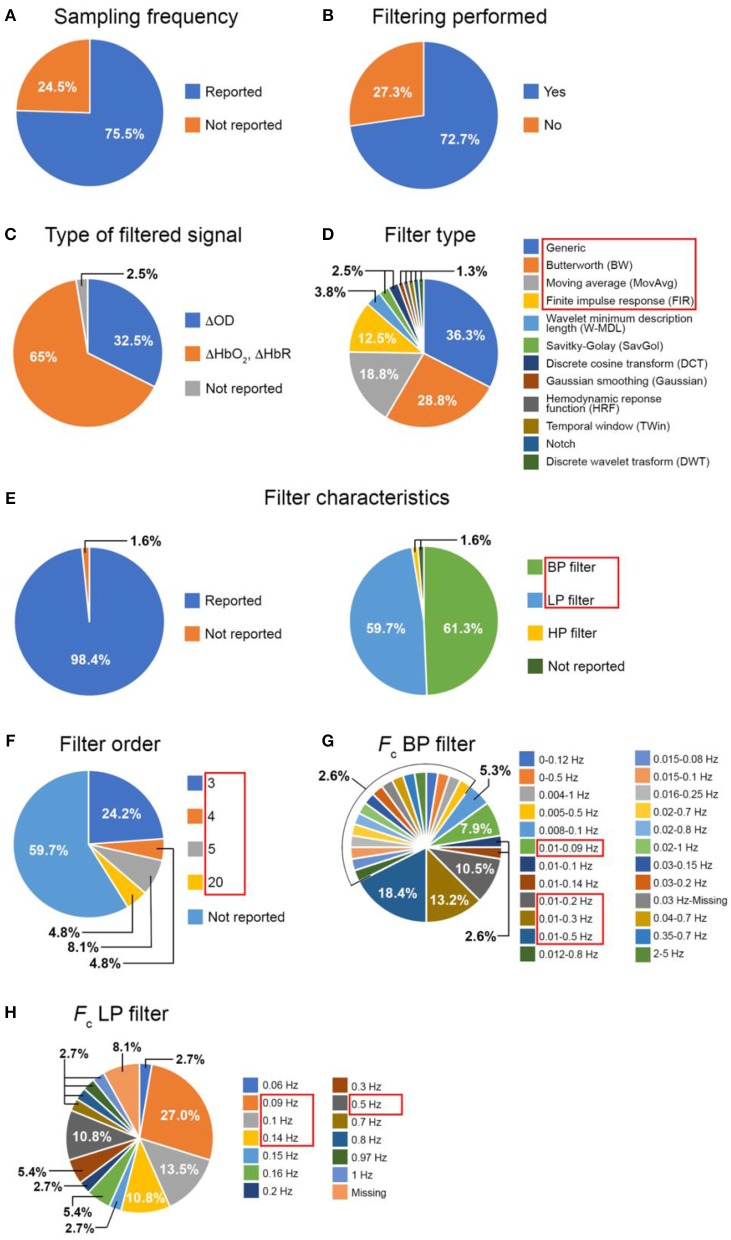
Summary of the literature review results: inclusion of **(A)** the *F*_s_ and of **(B)** a filtering step in the studies; proportion of the filtered signals **(C)**, filter type **(D)**, filter characteristics **(E)**, filter order **(F)**, *F*_*c*_ of band-pass **(G)** and low-pass **(H)** filters across the papers that included a filtering step. (BP, band-pass; LP, low-pass).

Note: If the authors stated in the paper that they used the Homer2 software package (http://homer-fnirs.org/) for their analysis and did not report any information about the filter type, we automatically considered they used a 3^rd^ order Butterworth filter as this is the default option in the software. Papers including more than one functional experiment within the same work were considered as separate studies.

A total of 110 papers were selected (see Supplementary Material 4 for a complete list). From each full-text, we collected the following information (Figure 2):

1. the sampling frequency (*F*_s_) of the fNIRS acquisition
2. the inclusion of the filtering step in the pre-processing stream
3. the type of filtered signal (ΔOD or ΔHbO_2_ and ΔHbR)
4. the type of filter applied (e.g., Butterworth, finite impulse response (FIR), Moving Average)
5. the filter characteristic (low-pass (LP), high-pass (HP), band-pass (BP) filtering)
6. the filter order, where applicable
7. the cut-off frequencies (*F*_*c*_)

Out of the 110 papers, 75.5% of the articles reported the *F*_s_ of the fNIRS acquisition (Figure 2A).

This result suggests that not all the papers report all the relevant information necessary for replicating or comparing the study results. Indeed, the *F*_s_ is an important parameter to evaluate the frequency bandwidth of the fNIRS signals or for assessing the filter stability within a certain frequency range. Additionally, as Figure 2C shows, there is not a clear agreement about whether it is a better practice to filter the optical density data or concentration data, and the fNIRS community is divided between the two approaches. In fact, for the papers we analyzed, the filter is applied on ΔOD signals in 32.5% of the studies and on ΔHbO_2_ and ΔHbR in 65%. The remaining 2.5% of the papers did not include this information.

Concerning the use of filters, the 72.7% of the papers included a filtering step in the pre-processing pipeline (Figure 2B). Figure 2D shows the distribution of the filter types across these papers. With “Generic” (Figure 2D) we refer to those filters for which the authors did not mention the particular filter type (e.g., ‘…data were band-pass filtered…'). The filter types shown in Figure 2D were both used individually or in combination with each other (e.g., W-MDL together with HRF). Within the majority of the papers (36.3%), the filter type was not properly described (i.e., Generic), further proving that not all the articles provide the most salient information, hence making it difficult for others to replicate the same procedure.

For the following analyses, we focused on the filter types being used in more than 3 papers (1.8%, Figure 2D, red rectangle), these are the Generic, Butterworth (BW), Moving Average (MovAvg) and Finite Impulse Response (FIR) filters. Among these filters, we determined how many articles included information about:

- the type of filter (LP/HP/BP, where applicable, i.e., Generic, BW, FIR)
- the filter order (where applicable, i.e., Generic, BW, FIR)
- the cut-off frequency ranges (where applicable, i.e., Generic, BW, MovAvg, FIR)

Results are presented in Figures 2E–H. Encouragingly enough only 1.6% of the papers did not include information about the type of filters (Figure 2E). Figure 2E also illustrates the distribution of the filter characteristics, showing that BP and LP are more often used rather than HP filters. However, concerning the filter orders (Figure 2F), the majority of the papers (59.7%) did not provide information about this parameter, which is really important for filters design. For our further analyses (see section Data Analysis), we have focused on BP and LP filters (Figure 2E red rectangle) since they are the most popular; and on all the filter orders (3, 4, 5, 20, Figure 2F red rectangle). Regarding the cut-off frequencies (Figures 2G,H), authors usually reports these except for the lower *F*_*c*_ for one BP filter (Figure 2G) and for 8.1% of the LP filters (Figure 2H). For our tests (see section Data Analysis), we used the *F*_*c*_ adopted by at least 3 papers (1.8%) and we indicated those with red rectangles in Figures 2G,H.

## Materials and Methods

### Participants

In order to investigate the effects of the filters on the outcome of statistical analyses, resting-state fNIRS data were collected on a cohort of healthy adults. Sixteen individuals (9 females, 7 males; age = 26.9 ± 2.9 years) were recruited and 18 sessions were performed. Prior the experiment, participants acclimated for about 15 min within the testing room. During the experiment, they were asked to keep their eyes closed for the entire session while being awake. The study was approved by the UCL ethics committee (Reference 1133/001) and participants gave informed consent prior to the experimental session.

### fNIRS Data Acquisition

**Figure 3:**
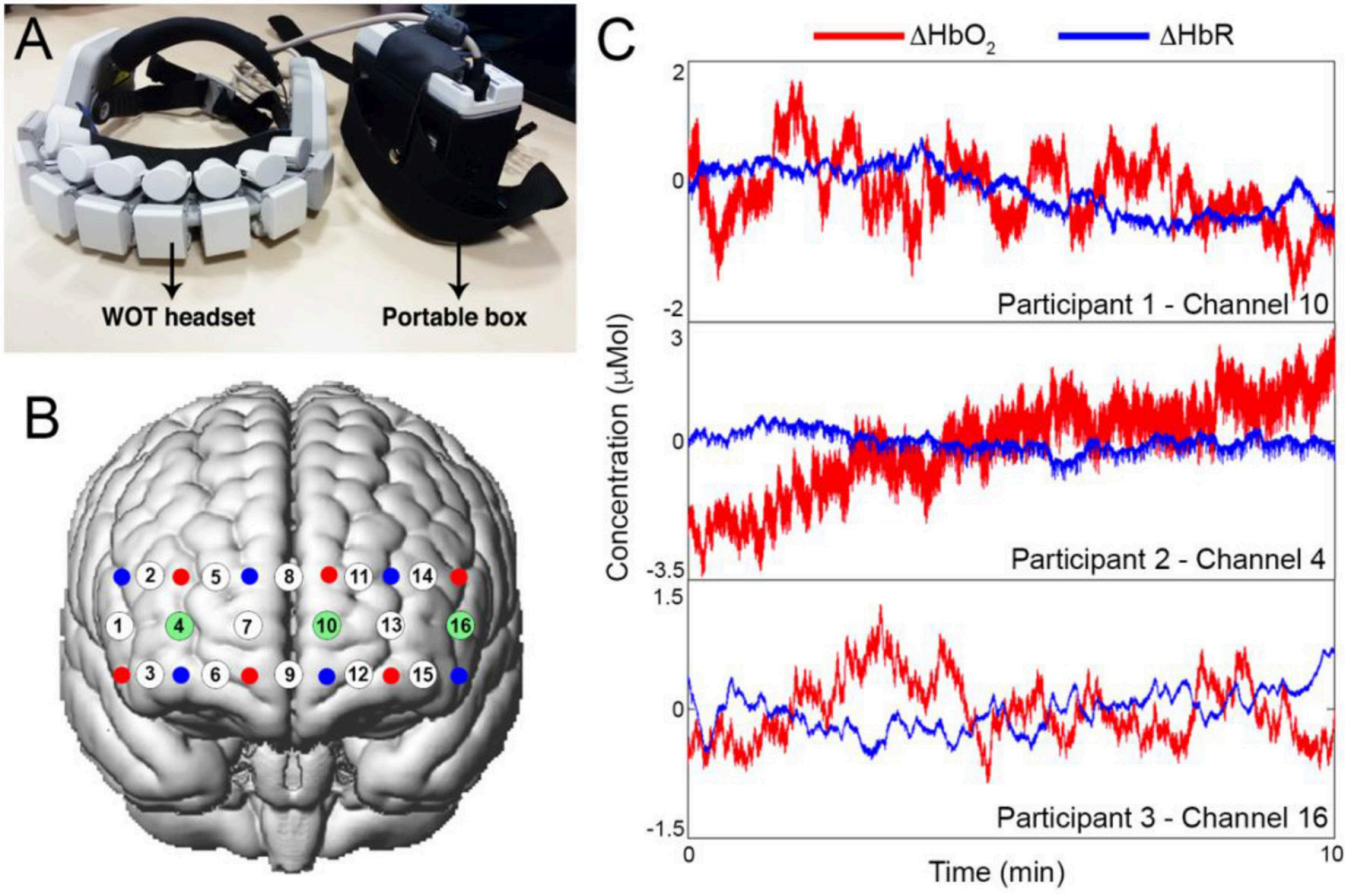
**(A)** Hitachi Wearable Optical Tomography fNIRS device, and corresponding channels configuration onto the prefrontal cortex **(B)**. Sources are represented as red dots, detectors as blue dots and channels as white dots. Highlighted in green are the channels for which the corresponding time-series are presented in **(C)**. **(C)** shows examples of raw ΔHbO_2_ and ΔHbR resting-state signals for one channel for each of three participants.

Spontaneous changes in prefrontal cortex hemodynamics were measured using the Wearable Optical Tomography (WOT, Hitachi High-technologies Corporation, Japan) fNIRS device. The system is made of a portable box, containing the recording unit, and a headset, containing the optical components (Figure 3A). The headset is equipped with 6 laser diodes emitting light at 705 and 830 nm, and 6 silicon photodiodes (Atsumori et al., 2009), arranged in an alternating geometry creating 16 measurement channels (Figure 3B; source-detector separation: 3 cm). Raw fNIRS data were recorded at 5 Hz. In order to place the WOT headset in a reliable way across all the participants, we used the 10/20 electrode positioning system (Okamoto et al., 2004) and placed channel 8 in correspondence of the Fpz point and channel 8 and 9 were aligned to the Nasion-Inion line. Resting-state data were collected for ~10 min while participants were comfortably sitting on a chair with their eyes closed. Examples of resting-state data for one channel from three subjects are shown in Figure 3C.

### Data Analysis

**Figure 4:**
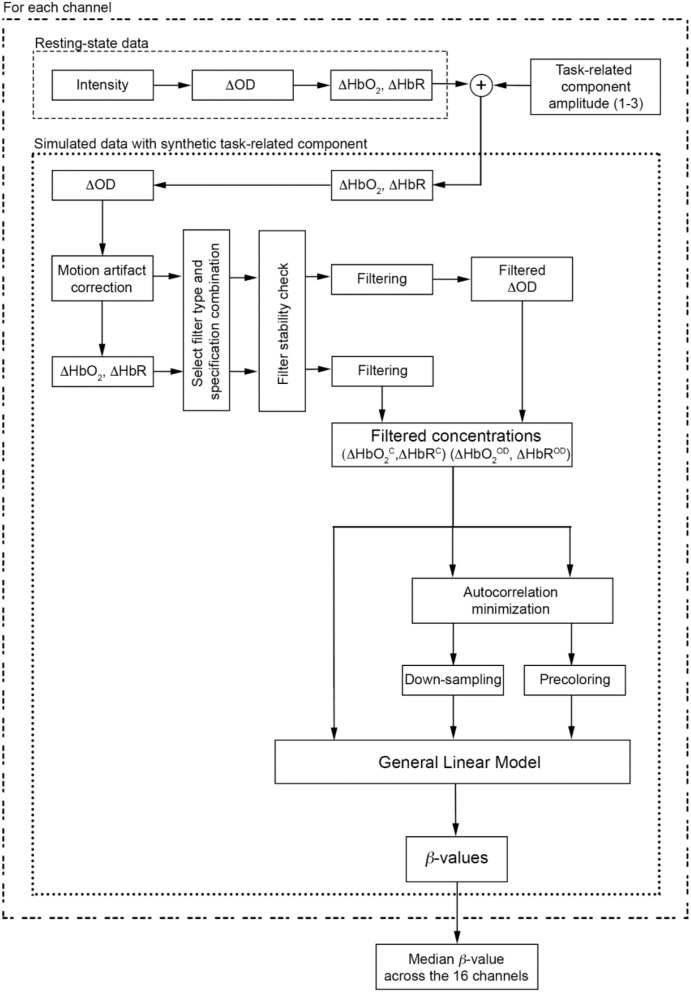
Data analysis flow chart applied to each participant and to each task-related component amplitude. The procedure is also applied for each filter type and specification combinations.

Single-subject's data analysis flowchart is presented in Figure 4.

Raw time-series were visually inspected to detect noisy channels (e.g., due to large motion errors, sudden amplitude changes, poor coupling) and channels with a poor optical coupling [e.g., absence of the ~1 Hz heartbeat oscillations in raw signals (Pinti et al., 2015)] were excluded from further analyses. Raw resting-state fNIRS data were first converted into optical density data and then into changes in concentration through the modified Beer-Lambert law, using a differential pathlength factor of 6 (Yücel et al., 2016). For all channels, a synthetic task-related component (the same for all 16 channels) simulating a block-design experiment was added to both ΔHbO_2_ and ΔHbR signals. This was created by convolving a Hemodynamic Response Function (HRF) with a boxcar function reflecting the simulated experimental protocol. The HRF was composed of two gamma functions, the positive one modeling the response and the negative one modeling the undershoot (peak: 6 s and undershoot: 16 s after the onset); the boxcar included 14 task blocks of 20 s spaced out by 20 s rest periods. This resulted in a stimulation frequency (*F*_*stim*_) of 0.025 Hz (*F*_*stim*_ = 1/(20+20) Hz). We used different amplitudes for the HbO_2_ and HbR task-related components, with the HbR one being ~-1/3 of the HbO_2_ component, as HbR has smaller changes than HbO_2_ (Gagnon et al., 2012). More precisely, in order to generate signals with different SNR, we considered the following amplitudes:

1. Amplitude 1: 0.8 μMol for ΔHbO_2_ and −0.27 μMol for ΔHbR
2. Amplitude 2: 0.5 μMol for ΔHbO_2_ and −0.17 μMol for ΔHbR
3. Amplitude 3: 0.3 μMol for ΔHbO_2_ and −0.1 μMol for ΔHbR

Three different synthetic datasets were thus generated for each of the 18 resting-state data.

Synthetic ΔHbO_2_ and ΔHbR signals were re-converted into ΔOD data and motion artifacts were identified and corrected (Figure 4) using the targeted principal component analysis (tPCA Yücel et al., 2014) implemented in the Homer2 software package, as it acts only on corrupted data segments, thus not altering the frequency content of the signals (function: *hmrMotionCorrectPCArecurse*; input parameters: *tMotion* = 0.5, *tMask* = 1, *STDthresh* = 10, *AMPthresh* = 5, *nSV* = 0.97, *maxIter* = 5). Optical density data corrected for motion errors were converted into ΔHbO_2_ and ΔHbR.

Based on the literature review (see section Literature Review), we filtered both synthetic ΔOD and, ΔHbO_2_ and ΔHbR signals using the filter specifications summarized in Table 1. All the *F*_*c*_ ranges include our *F*_*stim*_ (0.025 Hz). Filter orders of 100, 500, 1000 were also included in addition to the ones found in the literature, as FIR filters require higher orders than IIR filters (i.e., Butterworth) to achieve a good level of performance. More precisely, for each type of filter, we used all the combinations of filter type, filtered signals, filter order, and *F*_*c*_.

**Table 1:** Type of filter and filer specifications resulting from the literature review process.

**FILTER CHARACTERISTIC: BP**	
Filter type	BW, FIR
Filtered signals	ΔOD, ΔHbO_2, _ ΔHbR
Filter order	3, 4, 5, 20, 100*, 500*, 1000*
*F*_*c*_ [Hz]	0.01–0.09, 0.01–0.2, 0.01–0.3, 0.01–0.5
**FILTER CHARACTERISTIC: LP**	
Filter type	BW, FIR, MovAvg
Filtered signals	ΔOD, ΔHbO_2, _ ΔHbR
Filter order	3, 4, 5, 20, 100*, 500*, 1000*
*F*_*c*_ [Hz]	0.09, 0.1, 0.14, 0.5

*Asterisks indicate filter orders that were further added*.

Whilst FIR filters are always stable (i.e., for a finite input, the output is always finite, and the region of convergence of the transfer function of the filter includes the unit circle), IIR filters can be unstable for a given order and *F*_*c*_ (Ifeachor and Jervis, 2002). In fact, considering the transfer function of the filters, FIR filters have as many poles as zeros but they are all located at the origin of the *z*-plane, thus being always stable; by contrast, IIR filters are stable only if the poles are inside the unitary circle in the *z*-plane. Moving average filters operate by averaging the input signal within a certain window to produce the output signal. They are a particular type of low-pass FIR filters where the output signal is not multiplied by filter coefficients, but it is only scaled by 1/(window length). MovAvg are thus also always stable. Therefore, we first checked the stability of BP and LP Butterworth filters since they are IIR, for all the *F*_*c*_ and orders, using the zero-pole analysis, i.e., looking at the location of poles in the *z*-plane (for this procedure we used the Matlab functions *butter, isstable*, and *zplane*). Once the stability was assessed, we applied the type of filter with all the possible combination of specifications to synthetic ΔOD and, ΔHbO_2_ and ΔHbR signals. Filtered ΔOD were then converted into concentration changes. We will refer to ΔHbO${}_{2}^{\text{C}}$ and ΔHbR^C^ if the filter was applied directly to concentration data, and to ΔHbO${}_{2}^{\text{OD}}$ and ΔHbR^OD^ if the filter was applied to optical density data and then converted into concentration changes. In addition to the filters' stability, the phase delay introduced by the filter needs to be taken into account. In fact, the filtered signal can be shifted in time respect to the original unfiltered signal. In case of a FIR filter, the phase delay is constant, i.e., the same across the whole frequency range, and can be corrected by shifting back in time the filtered signal of the delay amount. With IIR filters (i.e., Butterworth), the phase delay is frequency-dependent, i.e., the shift is different for the different frequencies. This phenomenon is known as phase distortion and can be compensated using a zero-phase filter that we performed in Matlab with the *filtfilt* function.

Filtered concentration data were used to carry out statistical analyses and to establish the best filtering approach. The procedure described below was applied for each task-related component amplitude, to each channel of each participant's filtered signal (ΔHbO${}_{2}^{\text{C}}$, ΔHbR^C^, ΔHbO${}_{2}^{\text{OD}}$, ΔHbR^OD^), each type of filter (BP and LP), and each filter specification combination (Table 1). Statistical analyses were performed using the GLM approach (Figure 4). This method consists of regressing fNIRS data with a linear combination of explanatory variables (or regressors) and an error term. Regressors are computed through the convolution of the boxcar function describing the experimental protocol with the HRF (Friston et al., 1994). In our case, the design matrix was composed of the task-related regressor modeling the hemodynamic response to the simulated block-design experiment, plus the constant term. β-values were estimated through the least square estimation. These parameters are indicators of the strength of the relationship between a regressor and the experimental fNIRS data, and represent the contribution of each regressor to the fNIRS signal. However, fNIRS data are affected by serial autocorrelations due to the oscillating nature of the fNIRS signals (Barker et al., 2016) that impact on the accuracy of GLM-based analyses (Ye et al., 2009). Autocorrelations originate from the high sampling rate of fNIRS acquisition and from the physiological noises and motion errors present in the signals (Barker et al., 2016; Huppert, 2016). To account for serial autocorrelations and to minimize their impact on the GLM, we used two approaches: (i) down-sampling, and (ii) precoloring. In the first approach, we down-sampled the filtered concentration data to 1 Hz using a spline interpolation to reduce the sampling rate. Down-sampling the signal before the filter is applied can introduce a form of distortion in the data called aliasing, especially at the high-frequencies and when the new sampling rate is smaller than twice the highest frequency of interest in the signal (Nyquist frequency). To avoid this issue, low-pass filters (i.e., anti-aliasing filter) are typically used to remove the components above the new Nyquist frequency. In the second approach, we applied the precoloring method, i.e., smoothing the fNIRS data and the design matrix with a low-pass filter shaped like the HRF (Worsley and Friston, 1995; Huppert, 2016), which is a common method for analyzing fMRI and fNIRS data (Worsley and Friston, 1995; Ye et al., 2009). In order to test the impact of serial autocorrelations, we applied the GLM also to the filtered concentration data without any of these corrections (Figure 4). For each participant's data, the GLM was applied to each channel and each chromophore individually. β-values were then estimated for each channel and the median β-value across the 16 channels was computed for each participant. Median β-values for all the subjects were used to run statistical analyses at group-level. More precisely, we first checked for (i) the normality of the distribution of the group median β-values using the Shapiro-Wilk test as recommended for small sample sizes (Shapiro et al., 1968; Ghasemi and Zahediasl, 2012; < 50), and (ii) the presence of outliers. We considered as outliers when the β-values are below Q1 − 1.5 × IQR or above Q3 + 1.5 × IQR (Q1: 1^st^ quartile; Q3: 3^rd^ quartile; IQR: interquartile range). Amplitude 1, Amplitude 2 and Amplitude 3 of the imposed task-related components constitute the reference β-values and represent the metric to assess filters' performance. In fact, the closer are the estimated β-values to the reference β, the better the filter, i.e., less task-related information and more physiological noise were removed. Therefore, in order to establish the best type of filter, we used one sample *t*-tests to test the null hypothesis that the estimated group median β-values are equal to the reference β at a significance level α = 0.05. The closer the *p*-value to α, the more the group β-values are similar to the reference β, and thus the better the filter performance.

Additionally, we tested whether the filter performs better if applied to optical density or concentration data. To this goal, we used paired sample *t*-tests to compare the group β-values estimated on ΔHbO${}_{2}^{\text{C}}$ and ΔHbR^C^ with the group β-values estimated on ΔHbO${}_{2}^{\text{OD}}$ and ΔHbR^OD^, for a given type of filter and specification combinations.

All the analyses were carried out using Matlab (The MathWorks Inc., Natick, Massachusetts; v. R2014a) and the Homer2 software package.

## Results

**Figure 5:**
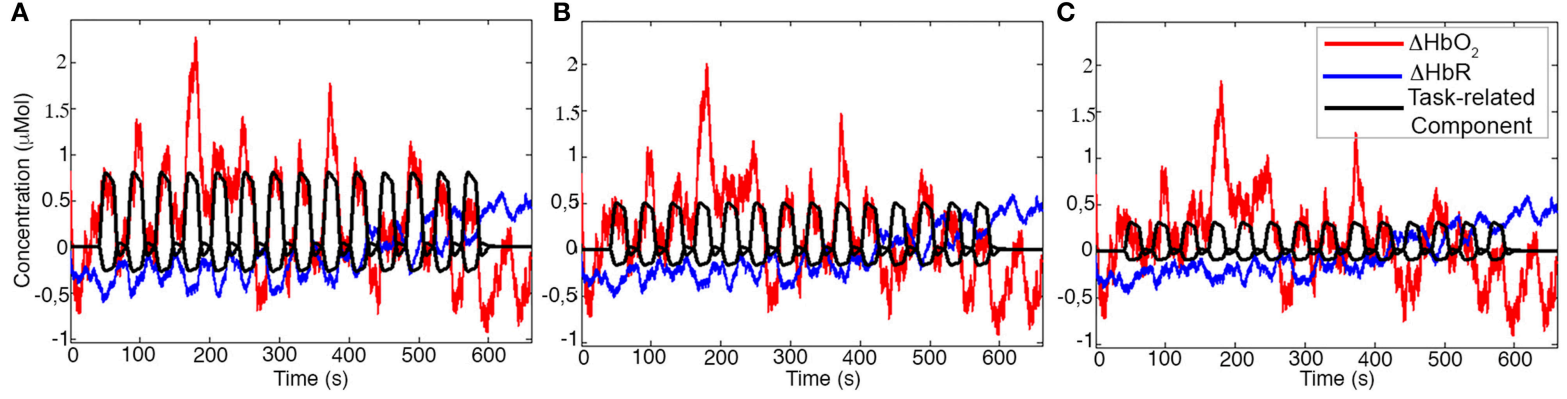
Examples of synthetic ΔHbO_2_ (red) and ΔHbR (blue) signals for one channel of a representative participant. The black signal represents the task-related component with Amplitude 1 **(A)**, Amplitude 2 **(B)**, and Amplitude 3 **(C)** added to the concentration data.

Examples of synthetic iconcentration data for a representative participant and channel generated using task-related components with Amplitude 1, Amplitude 2, and Amplitude 3 are shown in Figure 5.

**Figure 6:**
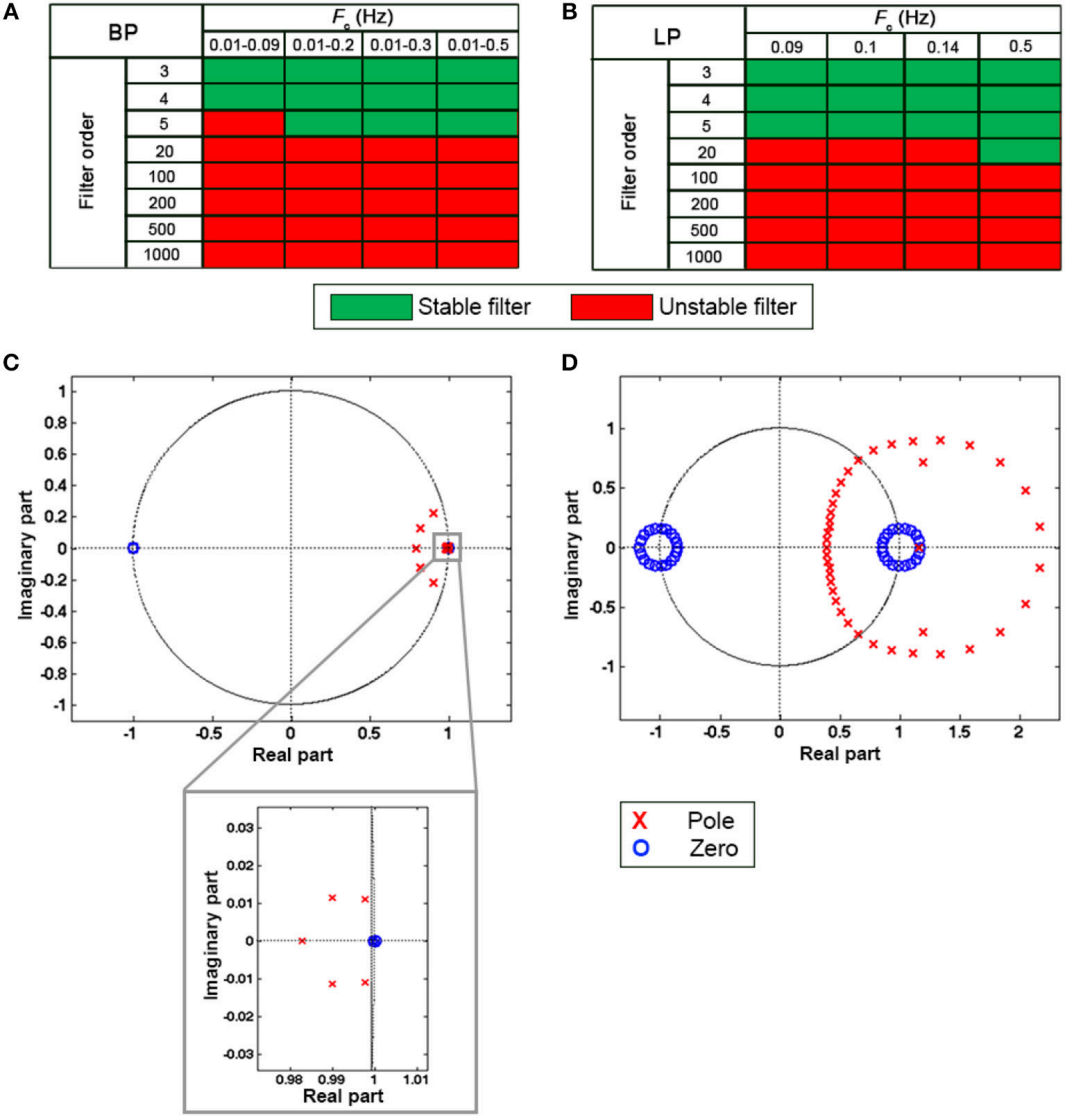
Results of the filter stability for the BP BW filters **(A)** and LP BW filters **(B)** for all the combination of orders and *F*_*c*_. Green and red squares indicate stable and unstable filters, respectively. **(C,D)** show examples of filter stability analysis considering a BW BP filter with *F*_*c*_ = [0.01 0.2] Hz. The filter results stable for a filter order 5 **(C)**, as the poles are inside the unitary circle as shown in the zoom. By contrast, with an order: 20 **(D)** the filter becomes unstable. Zeros are indicated by blue circles and poles by red crosses.

Due to the poor coupling between the fNIRS headset and the head, channel 11 was excluded from further analyses for participant 11, and channel 14 and 16 were excluded for participant 18. Synthetic datasets simulating a block-design experiment with 20 s task blocks were used to test the performance of filters in reducing the unwanted noise components in the fNIRS signals and in preserving the task-evoked hemodynamic response. To achieve this, we applied the type of filters and filter specifications summarized in Table 1 to the synthetic datasets. More precisely, we filtered both the ΔOD and, ΔHbO_2_ and ΔHbR time-series to determine the best signal to filter to obtain correct statistics. Prior the application of these filters, we tested the stability of BW filters for data sampled at 5 Hz using the zero-pole analysis, i.e., looking at the location of the poles of the filter transfer function with respect to the unitary circle in the *z*-plane. Filters with poles located within the unitary circles were considered stable. The procedure was applied to all the combinations of orders and *F*_*c*_ and to both BP and LP BW filters. Results for BP and LP filters are summarized in Figures 6A,B respectively. Green squares indicate stable filters, red elements indicate unstable filters.

For instance, a BW BP filter for data sampled at 5 Hz with order 5 and *F*_*c*_ = [0.01 0.2] Hz results stable as all the poles of the transfer function are inside the unitary circle (Figure 6C), whereas the same BW with order 20 is unstable (Figure 6D).

**Figure 7:**
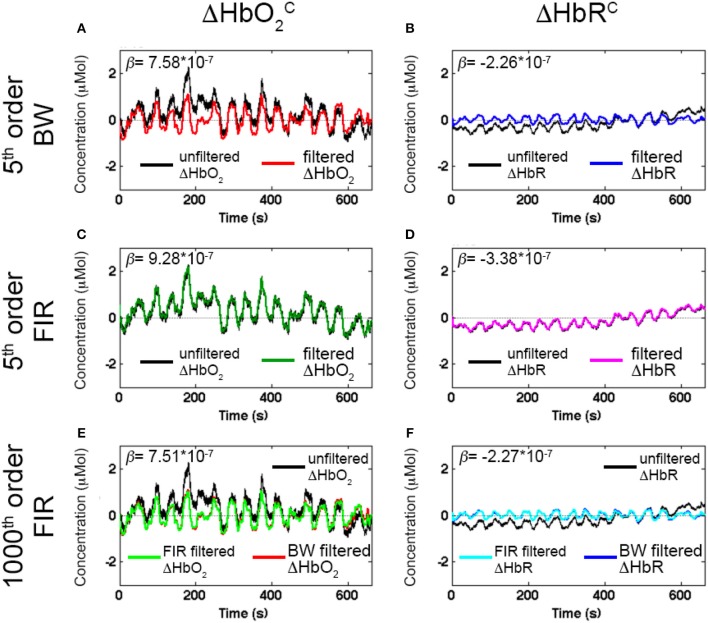
Examples of filtered ΔHbO${}_{2}^{\text{C}}$ and ΔHbR^C^ signals with Amplitude 1 for one channel of a representative participant using a 5th order BW BP filter (**A,B**, red and blue signals), a 5th order FIR BP filter (**C** and **D**, green and magenta signals), and a 1000th order FIR BP filter (**E,F**, light green and cyan signals) within the range [0.01, 0.2] Hz. The estimated β-values from the GLM fitting of the filtered ΔHbO${}_{2}^{\text{C}}$ and ΔHbR^C^ are included. The reference β are 8 × 10^−7^ for ΔHbO_2_ and of −2.7 × 10^−7^ for ΔHbR.

Figure 7 shows an example of filtered ΔHbO${}_{2}^{\text{C}}$ and ΔHbR^C^ signals using a BW BP filter (5th order, *F*_*c*_: 0.01–0.2 Hz, Figures 7A,B), FIR BP filters (5th order, *F*_*c*_: 0.01–0.2 Hz, Figures 7C,D) and FIR BP filters (1000th order, *F*_*c*_: 0.01–0.2 Hz, Figures 7E,F) to synthetic ΔHbO_2_ and ΔHbR, demonstrating the need for higher orders for FIR filters respect to IIR filters. The corresponding estimated β-values are reported as well.

Whilst the 5th order BW BP filter was able to remove the slow drifts in the unfiltered ΔHbO_2_ and ΔHbR signals (Figures 7A,B), a FIR filter with order 5 is not effective enough (Figures 7C,D). In fact, very low frequency modulations in the filtered ΔHbO_2_ signal can still be observed as well as a slow trend in the filtered ΔHbR (Figures 7C,D) and both signals are not centered around the zero-level. This results in an overestimation of the β-values (9.28 × 10^−7^ for ΔHbO_2_ and of −3.38 × 10^−7^ for ΔHbR). As a property of FIR filters, they require much higher orders than IIR filters to achieve comparable performance. As expected, with a 1000th order FIR filter, slow trends are effectively removed (Figures 7E,F), the signal mean is reported to be around the zero-level and a similar performance of the 5th order BW filter is achieved (light green and cyan signals in Figures 7E,F). The improvement in filters' performance is also reflected in the estimated β-values. The 1000th order FIR filter corresponds to a β-value of 7.51 × 10^−7^ for ΔHbO${}_{2}^{\text{C}}$ and of −2.27 × 10^−7^ for ΔHbR^C^ that are more similar to the reference β (8 × 10^−7^ for ΔHbO_2_ and of −2.7 × 10^−7^ for ΔHbR) than the estimated β-values for the 5th order FIR filter (β-value = 9.28 × 10^−7^ for ΔHbO${}_{2}^{\text{C}}$; β-value = −3.38 × 10^−7^ for ΔHbR^C^). More precisely, the β-values for the 5th order FIR filter are higher than the reference β because the slow trends of the signals were not removed effectively, worsening the GLM-fitting. The 1000th order FIR filter also performs similarly to the 5th order BW BP filter for which the β-values are 7.58 × 10^−7^ for ΔHbO${}_{2}^{\text{C}}$ and of −2.26 × 10^−7^ for ΔHbR^C^, demonstrating that FIR filters require higher orders than IIR to achieve comparable performance.

For each task-related component amplitude, and each type of filter and filter specification, ΔHbO${}_{2}^{\text{C}}$ and ΔHbR^C^, and ΔHbO${}_{2}^{\text{OD}}$ and ΔHbR^OD^ were used to run statistical analyses by means of the GLM approach. Since GLM-based analyses of fNIRS data can be influenced by serial autocorrelations, β-values were estimated (i) with no correction for serial correlations, (ii) down-sampling to 1 Hz the filtered data, (iii) using the precoloring method. The corresponding median β-values computed for each participant across the 16 measurement channels were used to assess filters performance. To achieve this, we first checked for the normality of the group β*-*values distribution using the Shapiro-Wilk test, testing the null hypothesis that median β-values are normal at significance level α = 0.05. Results referring to BP filters, Amplitude 1 and ΔHbO${}_{2}^{\text{C}}$ obtained using the precoloring method are shown in Table 2 and in Table 3 for ΔHbR^C^. The corresponding normality test results for LP and BP filters, all amplitudes, ΔHbO${}_{2}^{\text{OD}}$ and ΔHbR^OD^ are included in Supplementary Material 1.

**Table 2:** Shapiro-Wilk test results computed on ΔHbO${}_{2}^{\text{C}}$ BP filtered data, with Amplitude 1.

***Order/F*_*c*_**	**0.01–0.09 Hz**		**0.01–0.2 Hz**		**0.01–0.3 Hz**		**0.01–0.5 Hz**	
	**W_**(18)**_**	***p***	**W_**(18)**_**	***p***	**W_**(18)**_**	***p***	**W_**(18)**_**	***p***
**BW**								
3	0.91	0.10	0.91	0.07	0.91	0.07	0.90	0.07
4	0.92	0.14	0.91	0.08	0.91	0.08	0.91	0.08
5	-	-	0.93	0.21	0.93	0.20	0.93	0.19
20	-	-	-	-	-	-	-	-
100	-	-	-	-	-	-	-	-
200	-	-	-	-	-	-	-	-
500	-	-	-	-	-	-	-	-
1000	-	-	-	-	-	-	-	-
**FIR**								
3	0.77	1.04E-03	0.77	1.04E-03	0.77	1.04E-03	0.77	1.04E-03
4	0.77	1.04E-03	0.77	1.04E-03	0.77	1.04E-03	0.77	1.04E-03
5	0.77	1.03E-03	0.77	1.03E-03	0.77	1.03E-03	0.77	1.03E-03
20	0.77	9.85E-04	0.77	9.96E-04	0.77	1.01E-03	0.77	1.04E-03
100	0.77	1.09E-03	0.78	1.44E-03	0.78	1.43E-03	0.78	1.43E-03
200	0.85	1.14E-02	0.85	1.18E-02	0.85	1.17E-02	0.85	1.16E-02
500	0.92	1.09E-01	0.91	9.55E-02	0.91	9.23E-02	0.91	9.11E-02
1000	0.91	8.16E-02	0.91	7.44E-02	0.91	7.34E-02	0.91	7.34E-02

*W-values and corresponding p-values are reported. p < 0.05 are underlined, meaning that the null hypothesis that the β-values are normally distributed is rejected at significance level α = 0.05. Results are not reported in case of unstable filters. ‘-' indicates unstable filters for which the Shapiro-Wilk test was not carried out*.

**Table 3:** Shapiro-Wilk test results computed on ΔHbR^C^ BP filtered data, with Amplitude 1.

***Order/F*_*c*_**	**0.01–0.09 Hz**		**0.01–0.2 Hz**		**0.01–0.3 Hz**		**0.01–0.5 Hz**	
	***W*_**(18)**_**	***p***	***W*_**(18)**_**	***p***	***W*_**(18)**_**	***p***	***W*_**(18)**_**	***p***
**BW**								
3	0.95	0.48	0.95	0.50	0.95	0.50	0.95	0.48
4	0.95	0.51	0.95	0.44	0.95	0.47	0.95	0.48
5	-	-	0.95	0.50	0.95	0.47	0.95	0.47
20	-	-	-	-	-	-	-	-
100	-	-	-	-	-	-	-	-
200	-	-	-	-	-	-	-	-
500	-	-	-	-	-	-	-	-
1000	-	-	-	-	-	-	-	-
**FIR**								
3	0.81	3.33E-03	0.81	3.33E-03	0.81	3.33E-03	0.81	3.33E-03
4	0.81	3.33E-03	0.81	3.33E-03	0.81	3.33E-03	0.81	3.33E-03
5	0.81	3.32E-03	0.81	3.32E-03	0.81	3.32E-03	0.81	3.33E-03
20	0.81	3.11E-03	0.81	3.16E-03	0.81	3.23E-03	0.81	3.35E-03
100	0.82	4.25E-03	0.83	6.28E-03	0.83	6.25E-03	0.83	6.13E-03
200	0.94	2.97E-01	0.94	3.01E-01	0.94	3.01E-01	0.94	2.97E-01
500	0.93	1.76E-01	0.93	2.06E-01	0.93	2.09E-01	0.93	2.10E-01
1000	0.96	6.04E-01	0.96	6.04E-01	0.96	6.03E-01	0.96	6.01E-01

*W-values and corresponding p-values are reported. p < 0.05 are underlined, meaning that the null hypothesis that the β-values are normally distributed is rejected at significance level α = 0.05. Results are not reported in case of unstable filters. ‘-' indicates unstable filters for which the Shapiro-Wilk test was not carried out*.

Median β-values were normally distributed for BW filters (*p* > α) according to the Shapiro-Wilk normality test. By contrast, for ΔHbO${}_{2}^{\text{C}}$ the null hypothesis of normal distribution was rejected (*p* < α) for all the FIR filters with an order < 500 (Table 2) and with an order < 200 for ΔHbR^C^ (Table 3). In fact, as also shown in Figure 7, FIR filters require higher orders to effectively remove unwanted noise. For instance, with lower orders, slow trends in the signals related to e.g., instrumental noise or spontaneous physiological fluctuations are not properly filtered, introducing variability in the group β-values, since these types of noise can differ from subject to subject. As ΔHbR is less influenced by physiological interferences (Kirilina et al., 2012; Zhang et al., 2016) and there is thus less inter-subject variability, FIR filters with orders > 200 for are effective enough for ΔHbR. This variability results in outliers that alter the β-values distribution, as it can be observed in the box-plots in Supplementary Figures 9, 10 (Supplementary Material 2) referring to the data in Tables 2 and 3, respectively. The normality assumption is not violated when an order > 500 for ΔHbO_2_ and order > 200 for ΔHbR is used for FIR filters and no outliers are present (Supplementary Figures 9, 10 in Supplementary Material 2), further demonstrating the need of high orders.

The same is true for LP filters (Table 4 for ΔHbO${}_{2}^{\text{C}}$, and Table 5 for ΔHbR^C^), where median β-values never follow a normal distribution for any filter. In fact, especially in this case, slower signal modulations related to instrumental noise and slow vascular regulations are not filtered out since LP filters only attenuate noise with higher frequency content than the *F*_*c*_ reported in Table 1.

**Table 4:** Shapiro-Wilk test results computed on ΔHbO${}_{2}^{\text{C}}$ LP filtered data, with Amplitude 1.

**Order/*F*_***c***_**	**0.09 Hz**		**0.1 Hz**		**0.14 Hz**		**0.5 Hz**	
	***W*_**(18)**_**	***p***	***W*_**(18)**_**	***p***	***W*_**(18)**_**	***p***	***W*_**(18)**_**	***p***
**BW**								
3	0.77	1.04E-03	0.77	1.04E-03	0.77	1.03E-03	0.77	1.04E-03
4	0.77	1.05E-03	0.77	1.05E-03	0.77	1.03E-03	0.77	1.04E-03
5	0.77	1.08E-03	0.77	1.06E-03	0.77	1.04E-03	0.77	1.04E-03
20	-	-	-	-	-	-	0.77	1.04E-03
100	-	-	-	-	-	-	-	-
200	-	-	-	-	-	-	-	-
500	-	-	-	-	-	-	-	-
1000	-	-	-	-	-	-	-	-
**FIR**								
3	0.77	1.04E-03	0.77	1.04E-03	0.77	1.04E-03	0.77	1.04E-03
4	0.77	1.04E-03	0.77	1.04E-03	0.77	1.04E-03	0.77	1.04E-03
5	0.77	1.03E-03	0.77	1.03E-03	0.77	1.03E-03	0.77	1.03E-03
20	0.77	9.84E-04	0.77	9.85E-04	0.77	9.88E-04	0.77	1.03E-03
100	0.76	8.65E-04	0.77	9.39E-04	0.77	1.03E-03	0.77	1.05E-03
200	0.77	1.02E-03	0.77	1.05E-03	0.77	1.04E-03	0.77	1.05E-03
500	0.77	1.02E-03	0.77	1.05E-03	0.77	1.04E-03	0.77	1.04E-03
1000	0.77	1.04E-03	0.77	1.05E-03	0.77	1.04E-03	0.77	1.04E-03
**MovAvg**								
	0.77	1.03E-03	0.77	1.03E-03	0.77	1.04E-03	-	-

*W-values and corresponding p-values are reported. p < 0.05 are underlined, meaning that the null hypothesis that the β-values are normally distributed is rejected at significance level α = 0.05. Results are not reported in case of unstable filters and F_c_ = 0.5 Hz that corresponds to a null window length for the MovAvg filter. ‘-' indicates unstable filters for which the Shapiro-Wilk test was not carried out*.

**Table 5:** Shapiro-Wilk test results computed on ΔHbR^C^ LP filtered data, with Amplitude 1.

***Order/F*_*c*_**	**0.09 Hz**		**0.1 Hz**		**0.14 Hz**		**0.5 Hz**	
	***W*_**(18)**_**	***p***	***W*_**(18)**_**	***p***	***W*_**(18)**_**	***p***	***W*_**(18)**_**	***p***
**BW**								
3	0.81	3.38E-03	0.81	3.38E-03	0.81	3.38E-03	0.81	3.34E-03
4	0.81	3.36E-03	0.81	3.37E-03	0.81	3.38E-03	0.81	3.34E-03
5	0.81	3.37E-03	0.81	3.37E-03	0.81	3.38E-03	0.81	3.34E-03
20	-	-	-	-	-	-	0.81	3.34E-03
100	-	-	-	-	-	-	-	-
200	-	-	-	-	-	-	-	-
500	-	-	-	-	-	-	-	-
1000	-	-	-	-	-	-	-	-
**FIR**								
3	0.81	3.33E-03	0.81	3.33E-03	0.81	3.33E-03	0.81	3.33E-03
4	0.81	3.33E-03	0.81	3.33E-03	0.81	3.33E-03	0.81	3.33E-03
5	0.81	3.32E-03	0.81	3.32E-03	0.81	3.32E-03	0.81	3.33E-03
20	0.81	3.11E-03	0.81	3.11E-03	0.81	3.13E-03	0.81	3.34E-03
100	0.80	2.60E-03	0.81	3.05E-03	0.81	3.42E-03	0.81	3.40E-03
200	0.82	3.65E-03	0.82	3.72E-03	0.81	3.47E-03	0.81	3.41E-03
500	0.81	3.48E-03	0.81	3.51E-03	0.81	3.43E-03	0.81	3.36E-03
1000	0.81	3.58E-03	0.81	3.49E-03	0.81	3.42E-03	0.81	3.35E-03
**MovAvg**								
	0.81	3.31E-03	0.81	3.32E-03	0.81	3.33E-03	-	-

*W-values and corresponding p-values are reported. p < 0.05 are underlined, meaning that the null hypothesis that the β-values are normally distributed is rejected at significance level α = 0.05. Results are not reported in case of unstable filters and F_c_ = 0.5 Hz that corresponds to a null window length for the MovAvg filter. ‘-' indicates unstable filters for which the Shapiro-Wilk test was not carried out*.

In fact, outliers can be found for all the three filter types (Supplementary Figures 11, 12 in Supplementary Material 2). This also results in an overestimation of the β-values since the noise amplifies the signal amplitude and change its dynamics. This applies for all amplitudes and filtered signals (Supplementary Materials 1, 2). The use of LP filters on their own has thus not enough performance for denoising fNIRS data so that LP filters were excluded from further analyses.

Concerning the filter performance, we used one-sample *t*-tests to compare the group β-values to the reference β for each amplitude, filtered signals, type of filter and filter specifications. In addition, this was done for data not corrected for serial correlations, corrected through down-sampling and precoloring (Supplementary Material 3). In Table 6 and Table 7 we report the results referring to the β-values computed on ΔHbO${}_{2}^{\text{C}}$ and ΔHbR^C^ data corrected through the precoloring method, for Amplitude 1.

**Table 6:** One sample *t*-test results computed on ΔHbO${}_{2}^{\text{C}}$ BP filtered data, with Amplitude 1, comparing the group median β-values to the reference β, in case of precoloring correction.

***Order/F*_*c*_**	**0.01–0.09 Hz**		**0.01–0.2 Hz**		**0.01–0.3 Hz**		**0.01–0.5 Hz**	
	***t*_**(17)**_**	***p***	***t*_**(17)**_**	***p***	***t*_**(17)**_**	***p***	***t*_**(17)**_**	***p***
**BW**								
3	−5.12	8.59E-05	−5.10	8.98E-05	−5.09	9.04E-05	−5.11	8.69E-05
4	−5.32	5.62E-05	−5.22	6.87E-05	−5.22	6.93E-05	−5.25	6.49E-05
5	-	-	−5.09	9.04E-05	−5.20	7.25E-05	−5.20	7.27E-05
20	-	-	-	-	-	-	-	-
100	-	-	-	-	-	-	-	-
200	-	-	-	-	-	-	-	-
500	-	-	-	-	-	-	-	-
1000	-	-	-	-	-	-	-	-
**FIR**								
3	0.80	4.32E-01	0.88	3.91E-01	0.99	3.36E-01	1.32	2.04E-01
4	0.82	4.25E-01	0.95	3.57E-01	1.14	2.71E-01	1.68	1.12E-01
5	0.83	4.17E-01	1.03	3.18E-01	1.31	2.09E-01	2.05	5.58E-02
20	1.38	1.85E-01	3.11	6.32E-03	3.94	1.05E-03	2.35	3.13E-02
100	1.43	1.72E-01	−5.95	1.59E-05	−5.99	1.45E-05	−5.98	1.48E-05
200	−10.37	9.04E-09	−10.63	6.29E-09	−10.67	5.90E-09	−10.74	5.41E-09
500	−5.76	2.32E-05	−5.93	1.66E-05	−5.93	1.66E-05	−5.93	1.66E-05
1000	–4.73	1.93E-04	−4.93	1.27E-04	−4.92	1.28E-04	−4.92	1.29E-04

*Underlined is the highest negative t-value obtained for a 1000th order BP FIR filter. The t-value is negative as the reference β (0.8) is higher than the group median β-values (0.7). ‘-' indicates unstable filters for which the t-test was not carried out*.

**Table 7:** One sample *t*-test results computed on ΔHbR^C^ BP filtered data, with Amplitude 1, comparing the group median β-values to the reference β, in case of precoloring correction.

***Order/F*_*c*_**	**0.01–0.09 Hz**		**0.01–0.2 Hz**		**0.01–0.3 Hz**		**0.01–0.5 Hz**	
	***t*_**(17)**_**	***p***	***t*_**(17)**_**	***p***	***t*_**(17)**_**	***p***	***t*_**(17)**_**	***p***
**BW**								
3	4.94	1.23E-04	4.82	1.61E-04	4.85	1.49E-04	4.88	1.41E-04
4	5.15	8.06E-05	5.06	9.67E-05	5.05	9.95E-05	5.01	1.07E-04
5	-	-	4.96	1.19E-04	4.98	1.13E-04	4.94	1.25E-04
20	-	-	-	-	-	-	-	-
100	-	-	-	-	-	-	-	-
200	-	-	-	-	-	-	-	-
500	-	-	-	-	-	-	-	-
1000	-	-	-	-	-	-	-	-
**FIR**								
3	−3.05	7.31E-03	−3.12	6.24E-03	−3.23	4.94E-03	−3.55	2.45E-03
4	−3.06	7.12E-03	−3.18	5.42E-03	−3.37	3.63E-03	−3.90	1.16E-03
5	−3.07	6.90E-03	−3.26	4.58E-03	−3.54	2.54E-03	−4.27	5.22E-04
20	−3.59	2.26E-03	−5.28	6.12E-05	−6.10	1.19E-05	−4.55	2.81E-04
100	−3.65	1.99E-03	3.71	1.76E-03	3.75	1.59E-03	3.74	1.64E-03
200	10.09	1.35E-08	10.48	7.79E-09	10.52	7.33E-09	10.59	6.65E-09
500	5.36	5.23E-05	5.56	3.49E-05	5.55	3.54E-05	5.55	3.54E-05
1000	4.01	9.05E-04	4.24	5.49E-04	4.23	5.61E-04	4.22	5.71E-04

*Underlined is the lowest positive t-value obtained for a 1000th order BP FIR filter. The t-value is positive as the reference β (-0.27) is smaller than the group median β-values (−0.24). ‘-' indicates unstable filters for which the t-test was not carried out*.

For our experimental design with the *F*_*stim*_ of 0.025 Hz, we found that the best compromise across the three amplitudes, filtered signals, and in terms of outliers (Tables 2 and 3) is to use a BP FIR filter with order 1000 and *F*_*c*_ = [0.01, 0.09] Hz (Supplementary Material 3). In fact, the *F*_*c*_ range is more centered and narrower around the *F*_*stim*_ than the other *F*_*c*_ ranges (Table 1), and includes both the *F*_*stim*_ and the following two harmonics (2 × *F*_*stim*_ and 3 × *F*_*stim*_), maximizing the hemodynamic content and removing unnecessary frequency components. These filter specifications generally correspond to smallest *t*-value that means more similarity to the reference β, i.e., a better recovery of the hemodynamic response. Concerning the correction for serial autocorrelations, we found that the best results were obtained using the precoloring method (Ye et al., 2009), as the median β-values are more similar to the reference β for all the three amplitudes respect to the median β-values computed with no correction and down-sampling (Supplementary Material 3). This further establishes the precoloring as an effective way of accounting for autocorrelation in fNIRS signal and a fundamental step for GLM analyses (Ye et al., 2009).

We did not find statistically significant differences (*p* > 0.05) between corresponding β-values computed on ΔHbO${}_{2}^{\text{OD}}$/ΔHbR^OD^ and ΔHbO${}_{2}^{\text{C}}$/ΔHbR^C^, suggesting that it does not make any difference if the filter is applied to ΔOD data prior the conversion in concentration changes or to ΔHbO_2_ and ΔHbR (Supplementary Material 3).

## Discussion

Since fNIRS is one of the most recent neuroimaging modalities, there is no agreement yet about the way of analyzing data and describing the methodological details in research articles. We have identified 110 papers published in 2016 which reported task-related investigation of brain activity with fNIRS to identify the most common missing information that is critical for any study replication or comparison. More precisely, we found that nearly ¼ of the papers did not report the sampling frequency of the fNIRS acquisition, which is important for defining some preprocessing parameters (e.g., filters' cut-off frequencies). More than a half of the reviewed papers used BP filters to denoise fNIRS data and nearly half employed LP filters. Among the articles using BP filters, 24 different *F*_*c*_ were proposed with the most common being [0.01, 0.5] Hz (18.4% of the papers), and the most employed filter type was not defined (i.e. Generic, 36.3%) followed by Butterworth filters (28.8%). In terms of LP filters, a *F*_*c*_ of 0.09 Hz was most often used. However, important filtering parameters are very often missing in articles (see section Literature Review), especially the filter type (36.3%, Figure 2D) and the filter order (59.7%, Figure 2F). These are extremely important information that must be explicitly included in research papers to allow their full replication and understanding. In addition, there is not an agreement either on the filter type (Figure 2D) and the best signal to filter (Figure 2C).

In order to clarify these aspects and to start setting the ground for common practice in filtering and analyzing fNIRS data, we investigated the performance of the most frequently used band-pass and low-pass filters in terms of their influence on the outcome of the statistical inference step (Figure 1) in a GLM framework. The main findings of our simulation analysis using synthetic fNIRS data were:

1. there is no difference in outcome of the statistical analyses in terms of filtered signals (optical density or concentration, Supplementary Material 3)
2. low-pass filters and FIR filters with low orders (< 500) are not effective in removing the physiological VLF components and slow trends in the fNIRS signals, resulting in higher inter-subjects variability that impacts on group-level statistical analyses (section Materials and Methods, Supplementary Materials 1, 2, 3). LP filters should thus be combined with HP filters or detrending approaches (e.g., linear detrending) to remove very slow trends and VLF from fNIRS data
3. the best signal denoising is achieved using a BP FIR filter with high orders (e.g., >1000)
4. better results and more suitable statistics are obtained when correcting the GLM-analysis for serial correlations by means of the precoloring method (Supplementary Material 3).

Here, we have only tested three different types of filters with some specifications based on the most common practices adopted by the community. Further studies are needed that explore additional filtering methods in case of e.g., event-related design and block-design experiments with variable durations, and using additional parameter specifications. In the following section, we provide some recommendations and guidelines that we believe could help users in designing an appropriate filter for fNIRS data and in disseminating the research procedures in articles.

## Recommendations For Filter Design and The Way Forward

**Figure 8:**
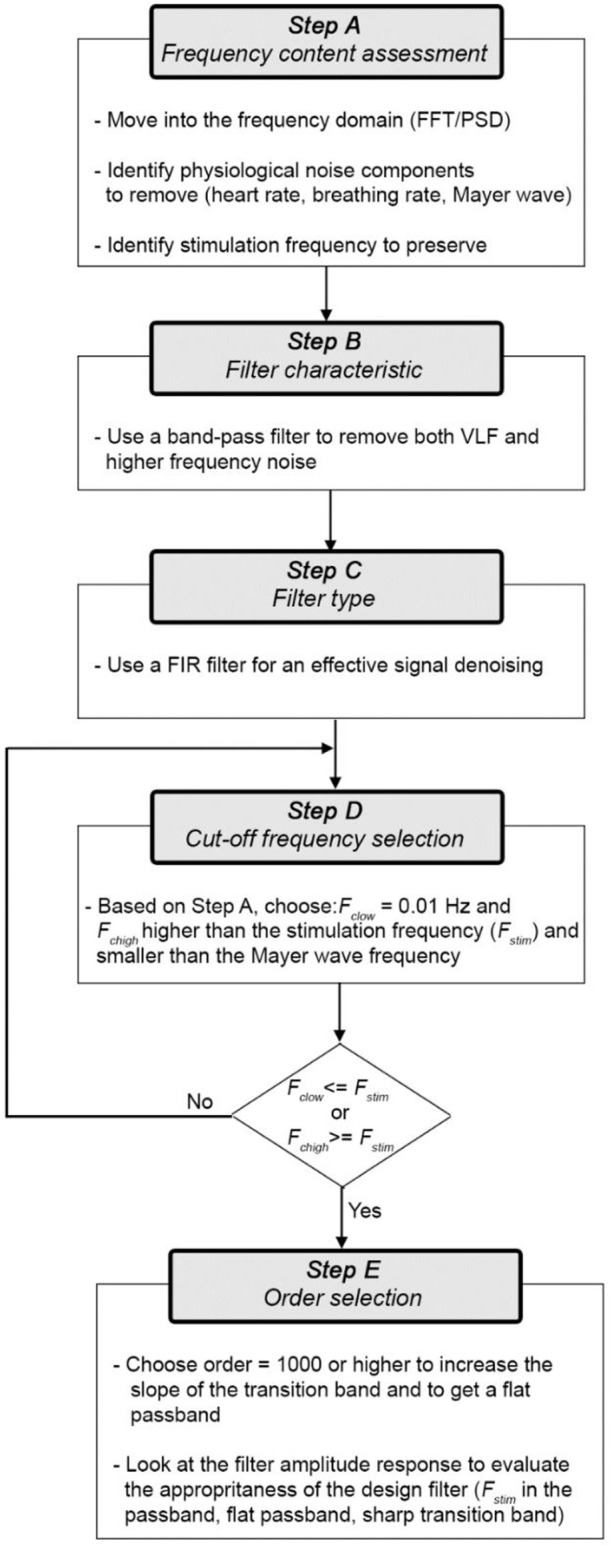
Digital filter design flow-chart.

Figure 8 shows the flow-chart of practical steps (A-E) that we advise to follow to design an effective filter for fNIRS data. Here, we consider ΔHbO_2_ and ΔHbR as the signals to filter, but the same flow-chart applies to ΔOD.

**Figure 9:**
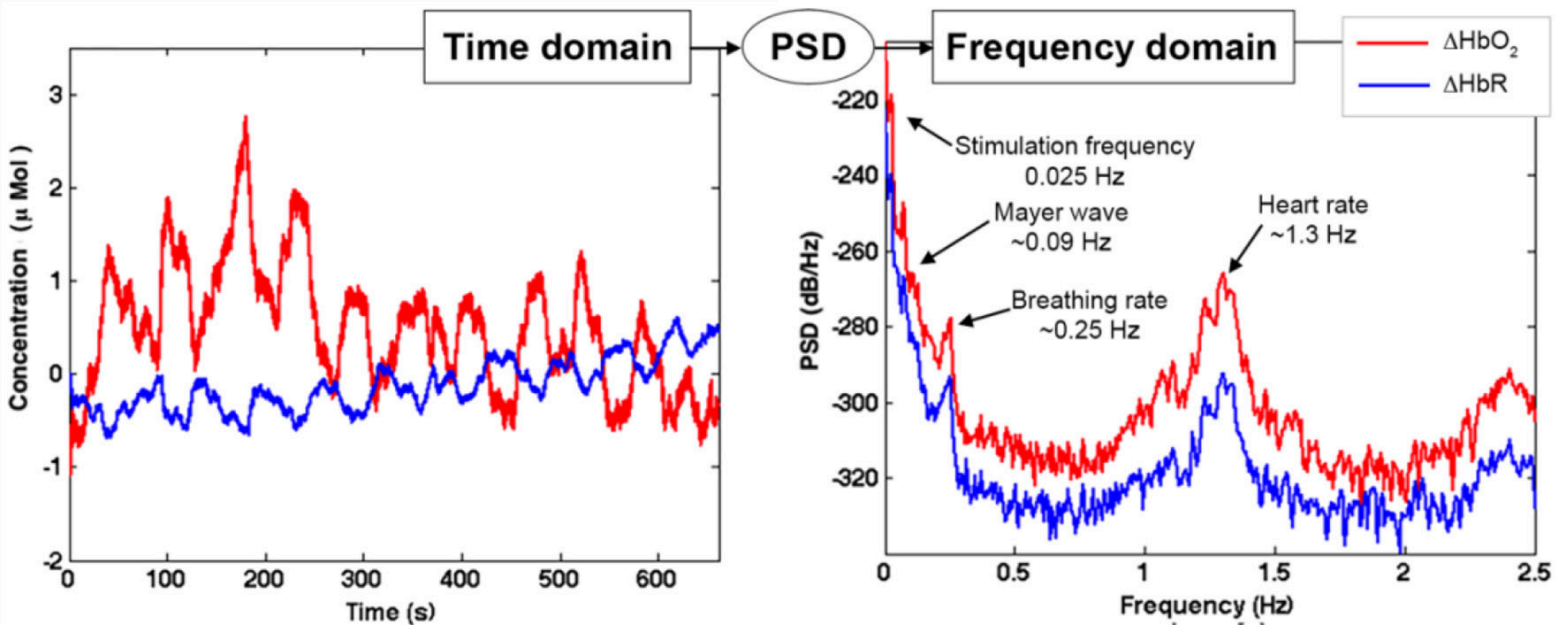
Example of ΔHbO_2_ and ΔHbR signals with Amplitude 1 for one channel of a representative participant in the time domain (left panel) and frequency domain (right panel). The PSD transforms the fNIRS signal from the time domain into the frequency domain. This allows the identification of the noise components (heart rate, breathing rate, Mayer waves) and the stimulation component, as shown in the left panel.

More precisely, the steps are the follows:

*Step A) Frequency content assessment:* the first step we advise to perform is to evaluate the frequency content of fNIRS signals. This will allow the identification of the stimulus frequency band to preserve and of the physiological noise components (e.g., heart rate, respiration, Mayer waves) to remove. To this goal, there are different algorithms that can be used to e.g., compute the Fast Fourier Transform (FFT) of the signal or the Power Spectral Density (PSD). For instance, in Figure 9 we used the Welch's estimation method to compute the PSD (function: *pwelch*; window length: 120 s; overlap: 50%) of the synthetic ΔHbO_2_ and ΔHbR signals to assess the physiological noises frequency ranges to remove. The PSD shows how the power of a signal is distributed as a function of frequency. From the PSD of the fNIRS signals of a representative participant (Figure 9), we can identify the heart rate component (~1.3 Hz), the respiration component (~0.25 Hz), and the Mayer wave component (~0.09 Hz); these are frequencies that we want to remove. We can also identify the stimulation frequency (*F*_*stim*_ = 1/40 s = ~0.025 Hz in our case) that we want to preserve; and that guides the choice of the *F*_*c*_ of the filter.

*Step B) Filter characteristic:* the first choice to make prior to designing a filter is the filter characteristic (BP/LP/HP). Based on the literature review (see section Literature Review) and our results, a BP filter achieves the highest performances in the outcome of statistical analyses. In fact, a LP filter alone is not enough as it does not remove the VLF frequencies corresponding to the very low vasomotion regulations and instrumental noise (e.g., low trends) (see section Materials and Methods).

*Step C) Filter type:* Different BP filters are available (e.g., FIR or IIR). Based on our results (see Section Materials and Methods), we recommend the use of BP FIR filters as they are (i) more stable and hence easier to control than IIR filters (i.e., the output is always finite), and (ii) do not introduce phase distortions and phase shift across the whole frequency band.

**Figure 10:**
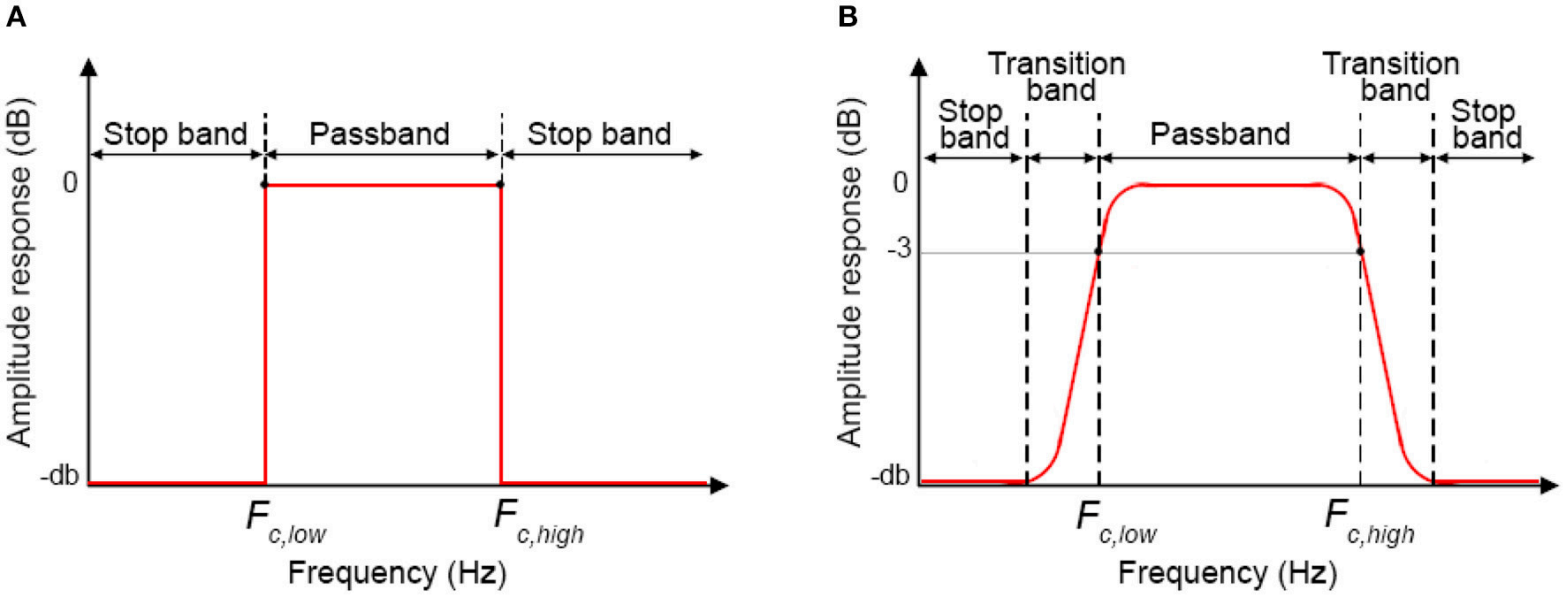
Filter amplitude response for ideal filters **(A)** and real filters **(B)**.

*Step D) Cut-off frequencies selection:* For BP filters, two cut-off frequencies must be selected. The lowest *F*_*c*_ (*F*_*c, low*_) will allow the frequencies higher than *F*_*c, low*_ to pass. The highest *F*_*c*_ (*F*_*c, high*_) will allow the frequencies lower than *F*_*c, high*_ to pass. In this way, *F*_*c, low*_ and *F*_*c, high*_ define the passband of the BP filter, i.e. the frequency range that can pass through the filter (Figure 10A).

The cut-off frequency choice is a compromise between noise reduction and hemodynamic signal maximization. In fact, whilst it is relatively easy to remove e.g., the heart rate component and the VLF such as those related to vascular endothelial regulations [ <0.01 (Yücel et al., 2016)], other components [e.g., Mayer waves or vascular neurogenic regulations (~0.04 Hz Yücel et al., 2016)] might overlap or be very close to the stimulation frequency. This must be taken into consideration when designing the experimental protocol, e.g., avoiding 10 s blocks overlapping the Mayer waves frequency and using variable rest durations. We also have to consider that it is impossible to design ideal digital filters (Figure 10A) where the filter amplitude response is rectangular with very sharp passband edges that allow an exact separation between passband and stopband and e.g., a precise separation between stimulation and noise frequencies. In reality, one also has to consider the transition band (which will depend on the filter order and type, see *Step E* and Figure 10B), which includes the frequency components that are progressively attenuated from −3 dB (i.e., the *F*_*c*_) to the total attenuation of the filter. Therefore, some of the signal's frequencies outside the passband will be attenuated and will still pass through the filter.

**Figure 11:**
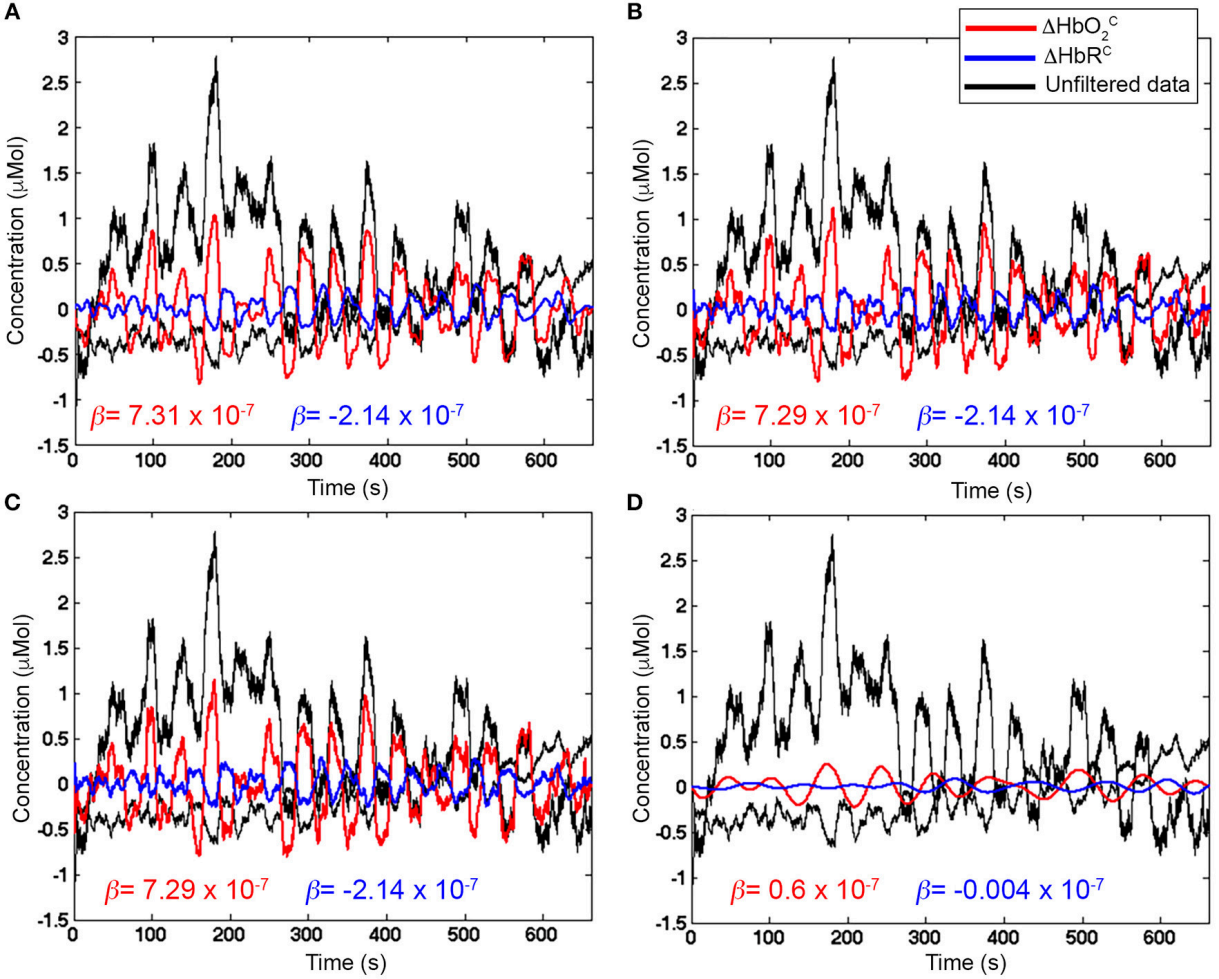
Examples of filtered ΔHbO_2_ (red) and ΔHbR (blue) signals with Amplitude 1 for one channel of a representative participant. Unfiltered ΔHbO_2_ and ΔHbR are presented in black. β-values (in red for ΔHbO_2_ and in blue for ΔHbR) are included as well. **(A)** shows properly filtered ΔHbO_2_ and ΔHbR data (BP FIR filter, order = 1,000, *F*_*c*_ = [0.01, 0.09] Hz) where the stimulation frequency (0.025 Hz) is correctly included in the *F*_*c*_ range, so that the hemodynamic response component is preserved and the β-values are the closest to the reference (8 × 10^−7^ for ΔHbO_2_ and of −2.7 × 10^−7^ for ΔHbR). **(B,C)** present filtered ΔHbO_2_ and ΔHbR (BP FIR filter, order = 1,000) with wider passband ranges (*F*_*c*_ = [0.01, 0.6] Hz and *F*_*c*_ = [0.01, 1.2] Hz, respectively) that let pass also unnecessary higher frequency noise (i.e., faster oscillations in the signals) that worsen the fit with the GLM approach. **(D)**, wrongly filtered ΔHbO_2_ and ΔHbR data (BP FIR filter, order = 1,000, *F*_*c*_ = [0.01 0.015] Hz) are presented, where the stimulation frequency (0.025 Hz) is not included in the *F*_*c*_ range, and the hemodynamic response component is strongly attenuated.

In our case with 20 s task-rest periods, the stimulation frequency (0.025 Hz) does not overlap with the Mayer wave component (~0.09 Hz). In this way, based on Figure 9, we can set *F*_*c, high*_ = 0.09 Hz so that the Mayer wave, breathing rate, heart rate components can be filtered out, and we include also the second and third harmonic of the fundamental stimulation frequency (i.e., 2 × *F*_*stim*_ and 3 × *F*_*stim*_) that still have substantial information. In terms of *F*_*c, low*_, *F*_*c, low*_ = 0.01 Hz is typically used (Figure 2). It allows to effectively remove very slow trends and vascular endothelial regulations (Yücel et al., 2016) in fNIRS signals, as slow as 100 s, and to preserve the stimulation frequency as task block/event durations smaller than 100 s are typically used. In case of stimulation protocols in which brain activity is expected to be sustained for periods longer than 100 s, then a smaller *F*_*c, low*_ should be used. Neurogenic regulations (~0.04 Hz) can be difficult to remove as they are really close to our stimulation frequency (0.025 Hz). By choosing a passband in the range [0.01, 0.09] Hz (Figure 11A), we can ensure that the stimulation frequency falls within the flat passband region (0 dB attenuation; Figure 10B) and is not attenuated, and that additional unnecessary components are not preserved. For instance, if higher *F*_*c, high*_ is used such as 0.6 Hz (Figure 11B) and 1.2 Hz (Figure 11C), higher frequency oscillations in the signals are included, worsening the GLM-fitting as shown by the estimated β-values that are more dissimilar to the reference β (8 × 10^−7^ for ΔHbO_2_ and of −2.7 × 10^−7^ for ΔHbR) than the ones obtained with the range [0.01, 0.09] Hz (Figure 11A).

Including the stimulation frequency in the flat passband—and in the passband in general—is extremely important to avoid removing the hemodynamic responses that can correctly pass through the filter (Figure 11A). If the *F*_*c, high*_ is lower than the stimulation frequency, for instance *F*_*c, high*_ = 0.015 Hz as shown in Figure 11D, the task-related component is strongly attenuated and can lead to false negatives in the statistical inference step, as proven by the very small β-values compared to the reference β.

**Figure 12:**
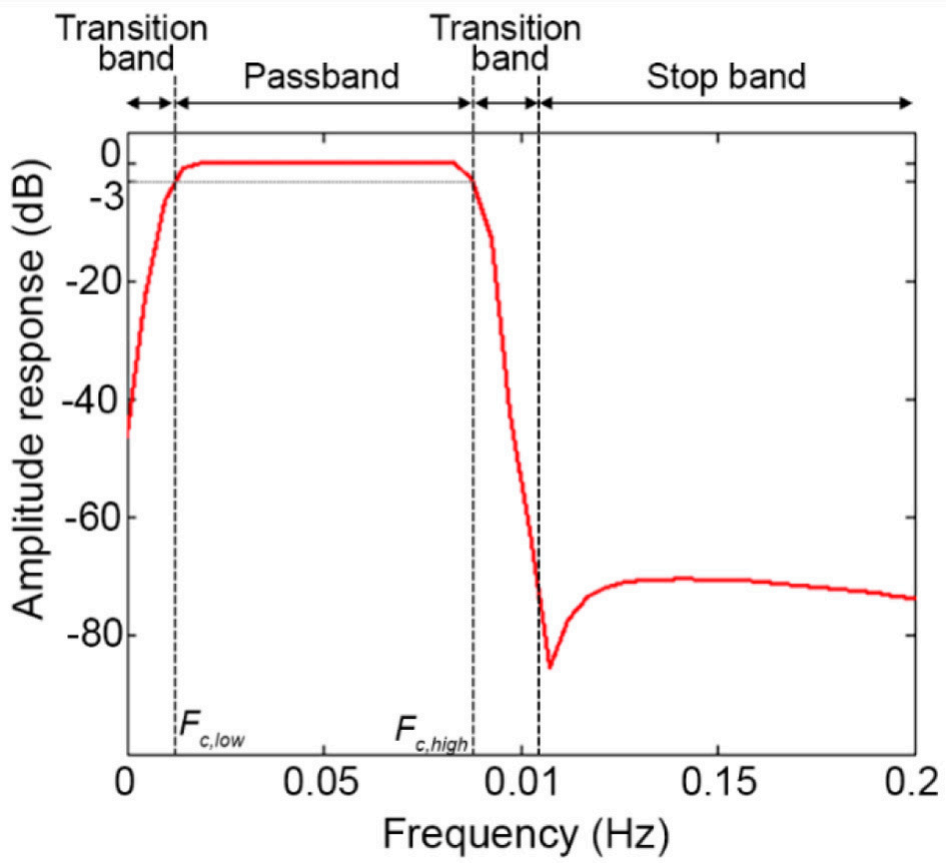
Filter amplitude response considering a BP FIR filter with order = 1000 and *F*_*c*_ = [0.01, 0.09] Hz.

In case the stimulation protocol has different task-rest durations, a stimulation frequency range [*F*_*stim*_*min*_ *F*_*stim*_*max*_] must be identified and preserved. *F*_*stim*_*min*_ is the inverse of the maximum block duration (e.g., the maximum rest duration + the maximum task duration); *F*_*stim*_*max*_ is the inverse of the minimum block duration (e.g., the minimum rest duration + the minimum task duration);

*Step E) Order selection:* In order to minimize the transition band (Figure 10B) and make the filter response more similar to the response of an ideal filter (Figure 10A), high filter orders should be used. This is not always possible with IIR filters because, as demonstrated in Figure 6, they can become unstable with higher orders in certain passband ranges. On the contrary, FIR filters are always stable and high orders can be used to maximize the performance. Based on our analyses, effective filtering can be achieved with order = 1000. Through the use of a high order and a passband with a range of [0.01, 0.09] Hz, we obtain a filter that has a flat passband region (0 dB attenuation) including the stimulation frequency and a narrow transition band (Figure 12; for illustration purposes, the frequency axis limit is set at 0.2 Hz).

For an effective filter design and to choose appropriate filters parameters, a useful tool is to look at the amplitude response of the filter [e.g., using the Matlab function *freqz* or the filter visualization tool (*FVtool*)] to optimize the passband based on the task design and the transition band. For instance, a sharper transition band can be achieved increasing the filter order (i.e., the higher the order, the higher the slope of the response in the transition band). Different formulas have been proposed for the estimation of the optimal FIR filter order to meet the design specification. The two oldest ones are the Kaiser's (Kaiser, 1974) and the Hermann-Rabiner-Chan's (Herrmann et al., 1973) formulas. The Kaiser's formula is the simplest and expresses the filter order as inversely proportional to the transition bandwidth (function *kaiserord* in Matlab). The estimation accuracy can decrease when the band ripples are not equal and the passband and stopband are very narrow respect to the transition band. Hermann-Rabiner-Chan's formula provides a solution for equiripple filters with either very narrow or very wide bandwidth. However, both formulas were optimized for filter orders smaller than ~150 and only for FIR filters with odd orders or length. New estimation methods were later proposed, e.g., Ichige et al. (2000) (Ichige et al., 2000), to overcome the abovementioned limitations.

**Figure 13:**
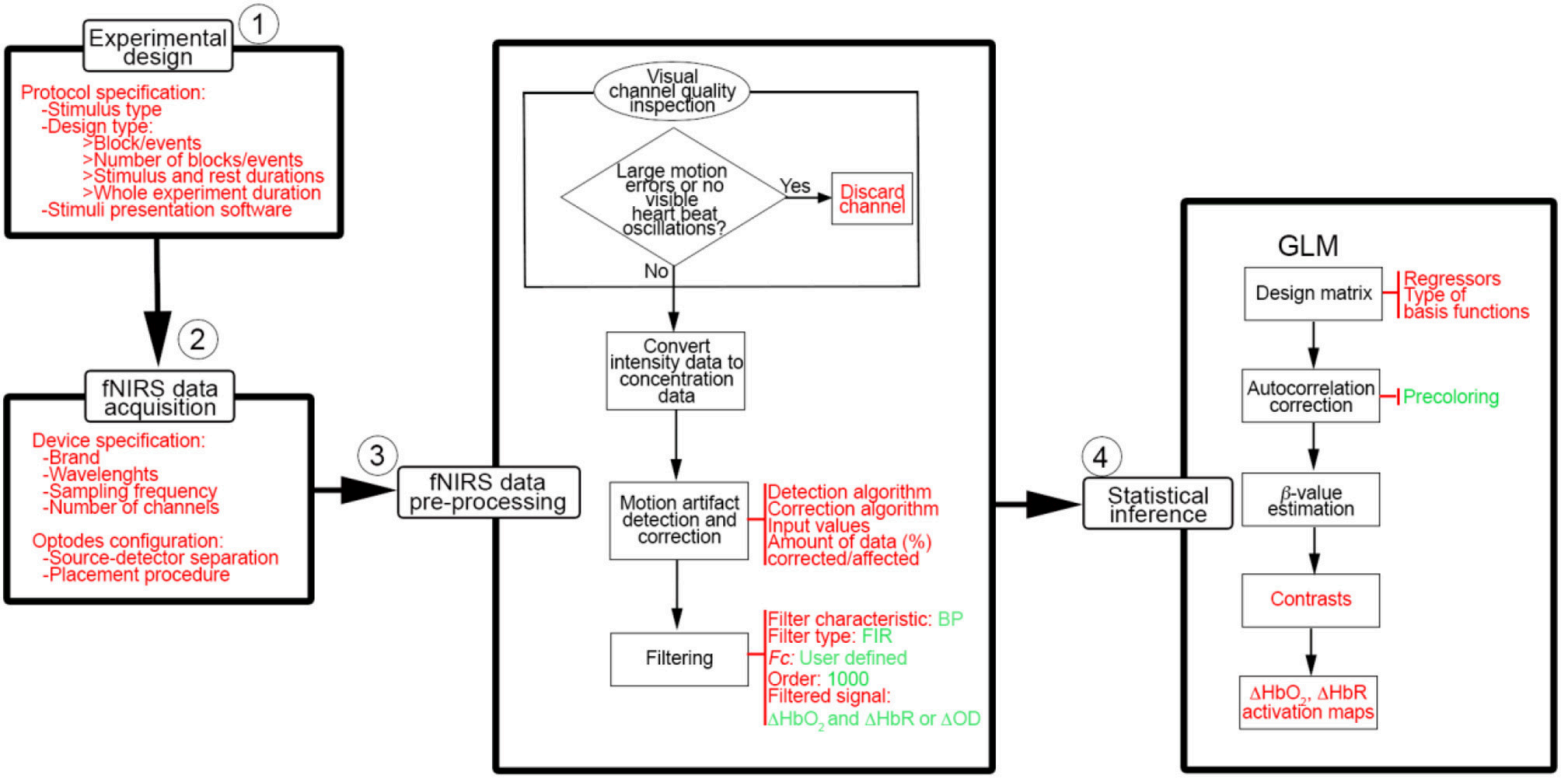
Basic workflow to conduct a typical neuroscience experiment with fNIRS. Information and parameters that we advise to report in research papers are indicated in red and the ones we recommend to use are presented in green.

Besides the optimization of fNIRS signals preprocessing, there are other aspects that have to be taken into consideration to improve the information communicated within the fNIRS papers. Following the experimental stream in Figure 1, we summarized in Figure 13 the workflow that we think should be applied when conducting a typical neuroscience experiment with fNIRS. More importantly, for each stage of the process, we have indicated in red the information that we recommend to use and report in the methods section of any fNIRS research article.

Our recommendations refer to basic procedures and the workflows shown in Figures 12, 13 can be expanded with further improvements, such as integrating fNIRS measurements with simultaneous systemic physiology recordings or using short-separation channels to allow a better interpretation of fNIRS neuroimaging data and to formulate more accurate neuroscientific conclusions (Tachtsidis and Scholkmann, 2016). For instance, these measurements can be easily integrated in the GLM framework as additional regressors in the design matrix, making this approach even more powerful and versatile. Moreover, other approaches can be included as an additional step between phase 3 and phase 4 of the workflow in Figure 13, such as the principal component spatial filter developed by Zhang and colleagues (Zhang et al., 2016) to remove the global systemic effects from fNIRS data, or combining the HbO_2_ and HbR signals in e.g., the activation signal [through the correlation-based signal improvement (Cui et al., 2010)], total hemoglobin (HbT = HbO_2_ + HbR) or hemoglobin difference [Hb_diff_ = HbO_2_ – HbR (Tachtsidis et al., 2009)] and use the combined signal to carry out the statistical inference. However, the present workflows (Figures 12, 13) represent the starting point toward an improvement and standardization of fNIRS studies that could guide the community through all the phases of a neuroscience experiment with fNIRS.

## Author Contributions

PP, FS, and IT conceived and designed the study. PP carried out the data acquisition. PP and FS analyzed the data. PP, FS, IT, AH, and PB contributed to the interpretation of the results and to the manuscript writing. All authors provided critical feedback and helped shape the research, analysis, and manuscript.

### Conflict of Interest Statement

The authors declare that the research was conducted in the absence of any commercial or financial relationships that could be construed as a potential conflict of interest.
