# Supplementary material for: Current Status and Issues Regarding Pre-processing of fNIRS Neuroimaging Data: An Investigation of Diverse Signal Filtering Methods Within a General Linear Model Framework
Source: Front Hum Neurosci. 2019 Jan 11;12:505. doi: 10.3389/fnhum.2018.00505 (PMC6336925; doi:10.3389/fnhum.2018.00505)
Supplement: Supplementary file 2 [file Table_2.DOCX]

**1. Assessment of median *β*-values distribution and outliers at group-level**

Simulation analyses with synthetic fNIRS data were carried out with the aim of identifying the best filtering method to optimize the statistical analysis of fNIRS data in a GLM approach, and to better recovery the hemodynamic response (see Section 3.3 in the main text). Synthetic HbO_2_ and HbR signals were generated using task-related components with three amplitudes:

1. Amplitude 1: 0.8 μMol for ΔHbO_2_ and -0.27 μMol for ΔHbR
2. Amplitude 2: 0.5 μMol for ΔHbO_2_ and -0.17 μMol for ΔHbR
3. Amplitude 3: 0.3 μMol for ΔHbO_2_ and -0.1 μMol for ΔHbR

Synthetic fNIRS data were then filtered using the filter characteristics summarized in Table 1 in the main text, and analyzed through the GLM method. More precisely, for each participant, *β*-values were first estimated for each channel through the least square estimation, and the median *β*-value was then calculated across the 16 measurement channels. To investigate the effect of serial autocorrelations, the GLM was applied (i) using no correction, (ii) down-sampling the data to 1 Hz, (iii) using the precoloring method. In the sections below, we report the boxplots referring to the group median *β*-values for each task-related component amplitude and autocorrelation correction, computed on ΔHbO_2_^C^ and ΔHbR^C^ using the different filter characteristics (Table 1 in the main text).

**1.1. Boxplots amplitude 1**

**1.1.1. No correction for serial autocorrelations**


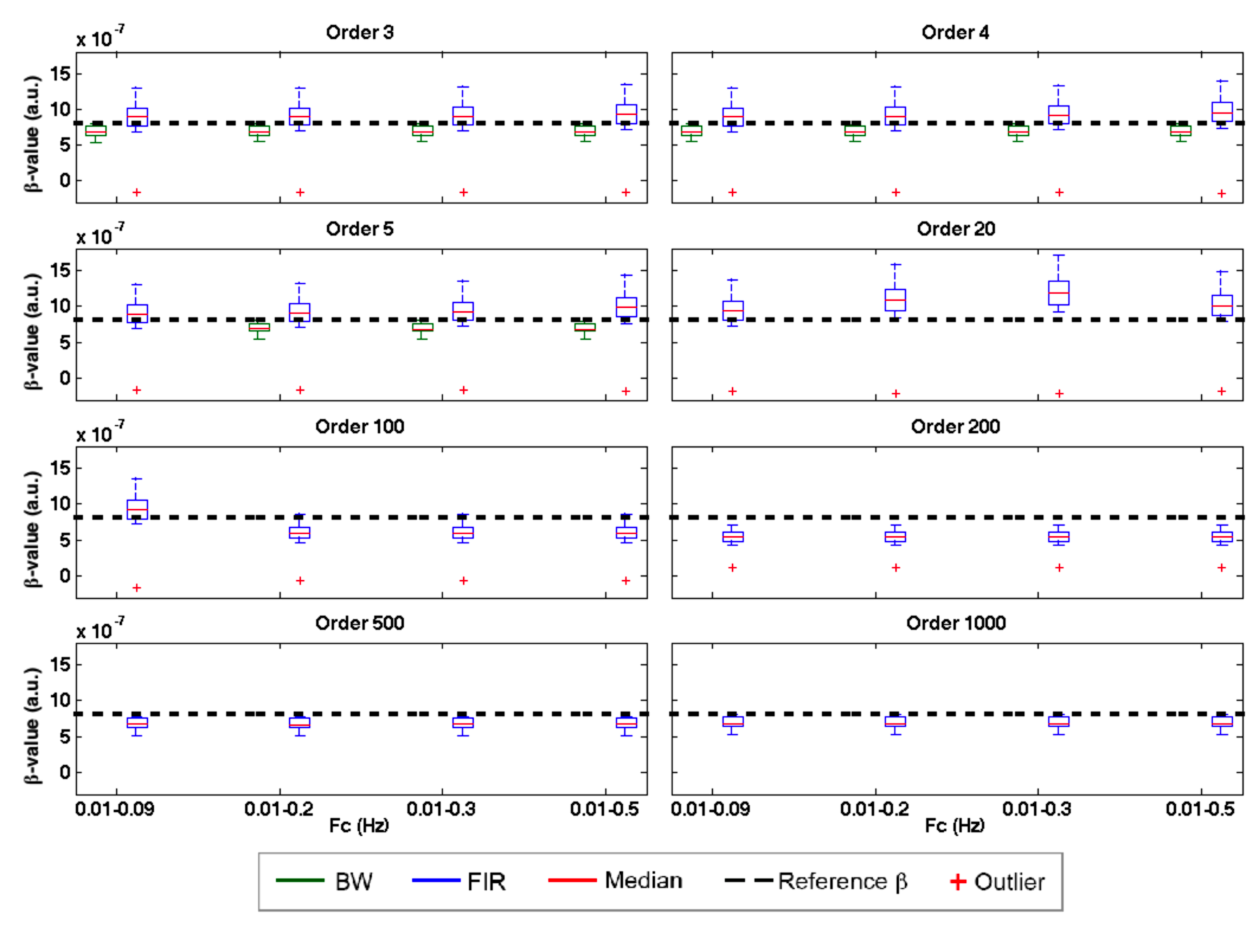


**Supplementary figure 1.** Boxplots referring to ΔHbO_2_^C^ BP filtered (green: BW; blue: FIR) data, with Amplitude 1 and no autocorrelation correction. Outliers are indicated as red crosses and can be observed in case of filters with low performance in signal denoising. The black dashed line represents the value of the reference *β*. Boxplots are not reported in case of unstable filters.

**
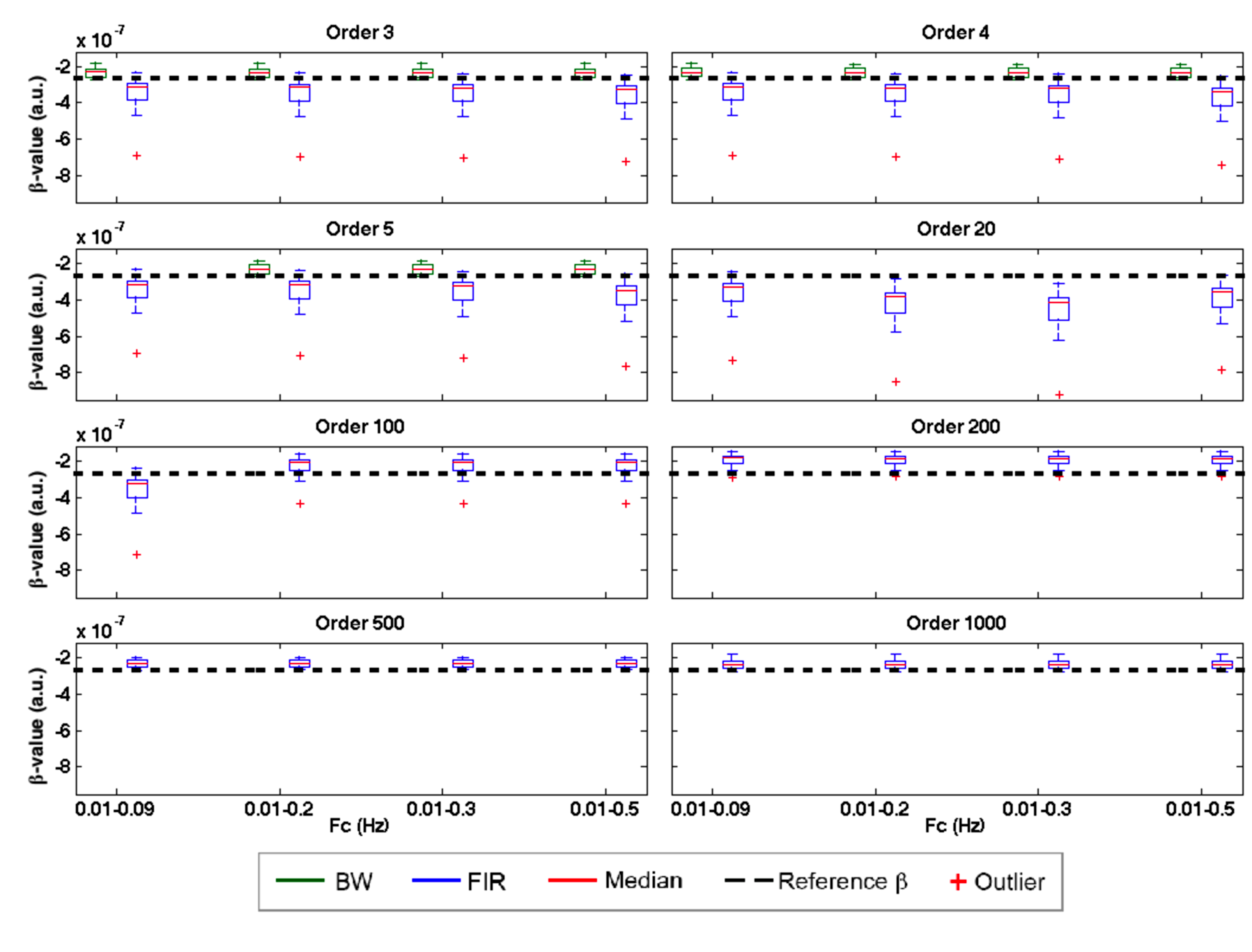
**

**Supplementary figure 2.** Boxplots referring to ΔHbR^C^ BP filtered (green: BW; blue: FIR) data, with Amplitude 1 and no autocorrelation correction. Outliers are indicated as red crosses and can be observed in case of filters with low performance in signal denoising. The black dashed line represents the value of the reference *β*. Boxplots are not reported in case of unstable filters.

**
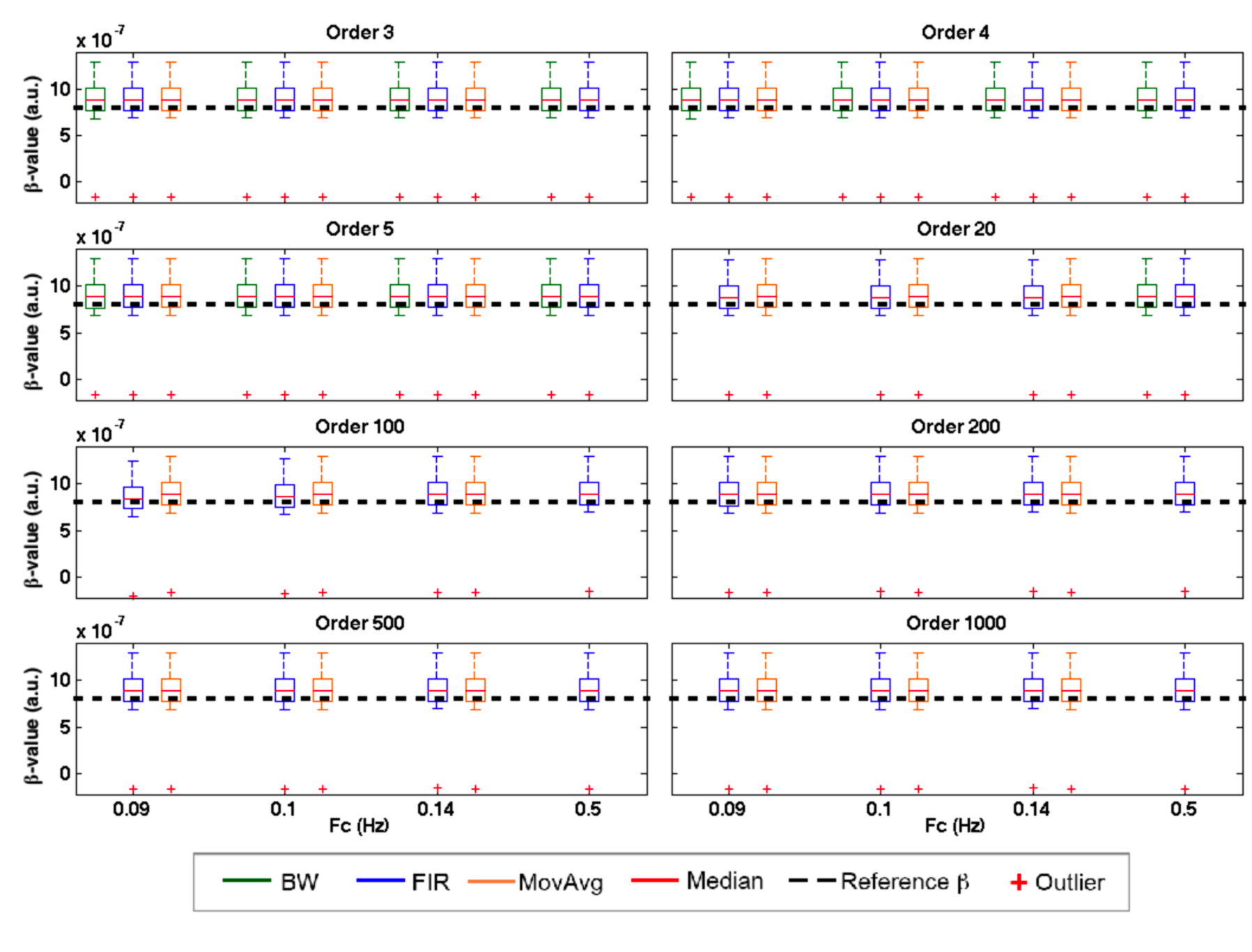
**

**Supplementary figure 3.** Boxplots referring to ΔHbO_2_^C^ LP filtered (green: BW; blue: FIR; orange: MovAvg) data, with Amplitude 1 and no autocorrelation correction. Outliers are indicated as red crosses and can be observed in case of filters with low performance in signal denoising. The black dashed line represents the value of the reference *β*. Boxplots are not reported in case of unstable filters and for *F*_c_ = 0.5 Hz for the MovAvg filter that corresponds to a null window length.

**
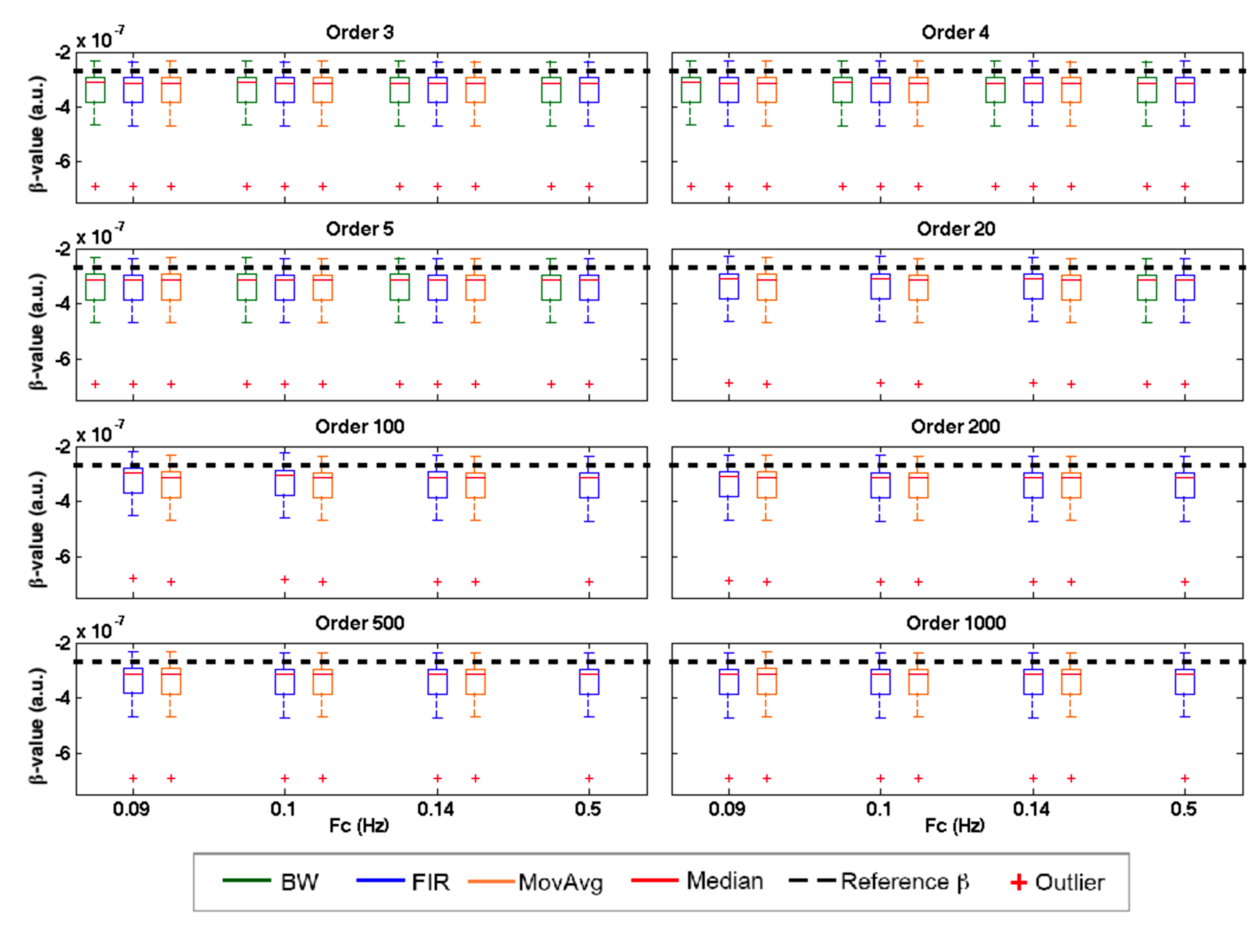
**

**Supplementary figure 4.** Boxplots referring to ΔHbR^C^ LP filtered (green: BW; blue: FIR; orange: MovAvg) data, with Amplitude 1 and no autocorrelation correction. Outliers are indicated as red crosses and can be observed in case of filters with low performance in signal denoising. The black dashed line represents the value of the reference *β*. Boxplots are not reported in case of unstable filters and for *F*_c_ = 0.5 Hz for the MovAvg filter that corresponds to a null window length.

**1.1.2. Down-sampled data**


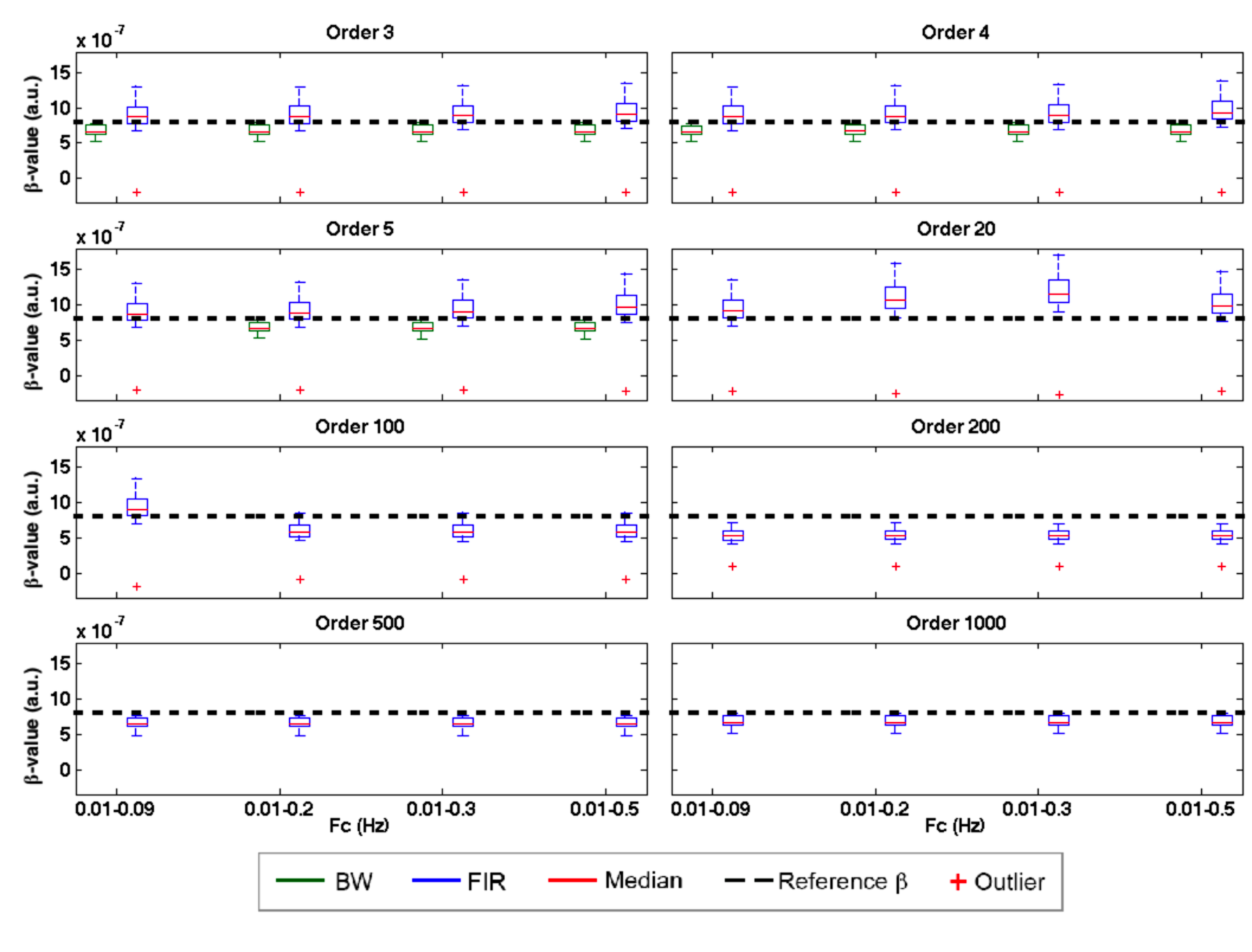


**Supplementary figure 5.** Boxplots referring to ΔHbO_2_^C^ BP filtered (green: BW; blue: FIR) data, with Amplitude 1 and down-sampled data. Outliers are indicated as red crosses and can be observed in case of filters with low performance in signal denoising. The black dashed line represents the value of the reference *β*. Boxplots are not reported in case of unstable filters.

**
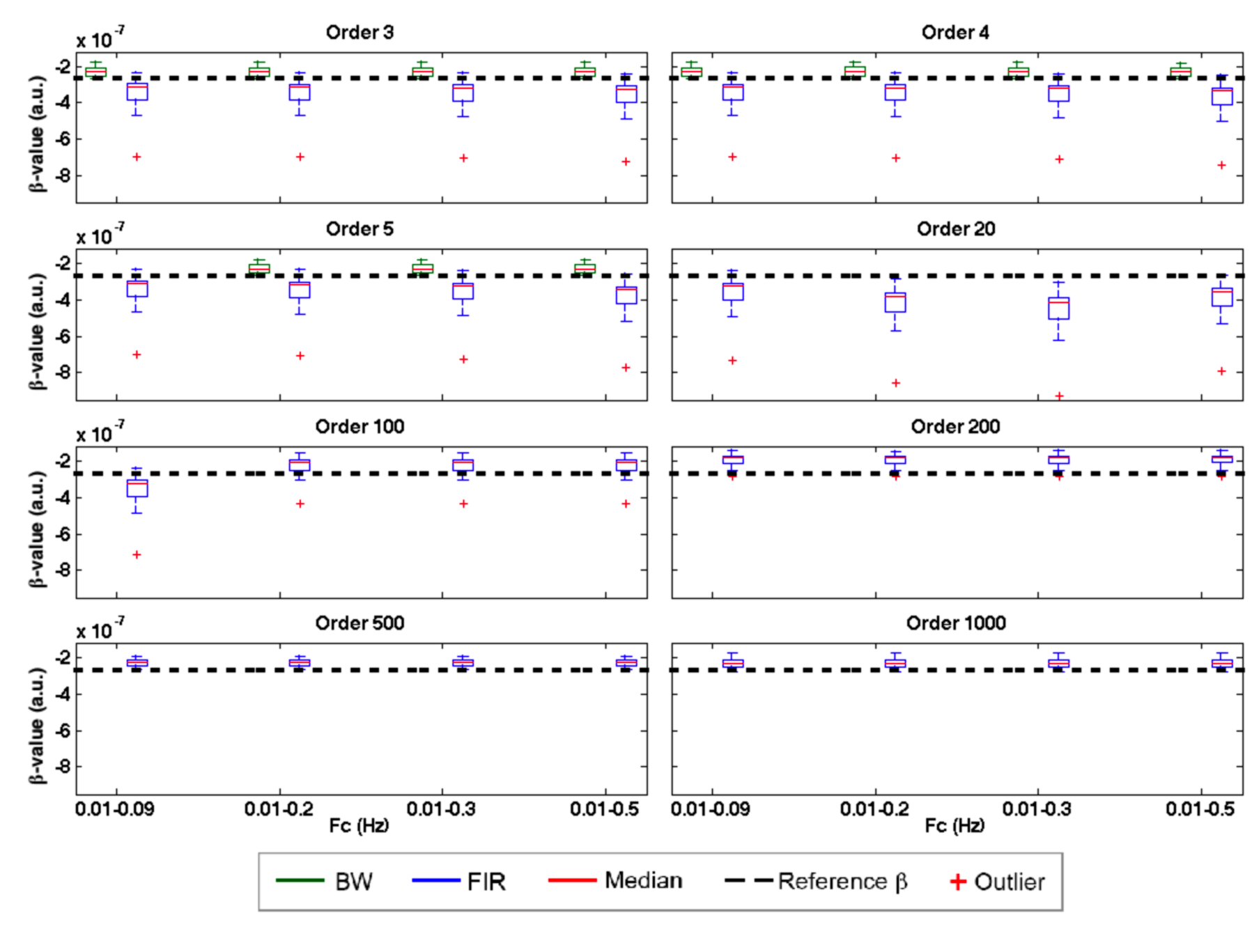
**

**Supplementary figure 6.** Boxplots referring to ΔHbR^C^ BP filtered (green: BW; blue: FIR) data, with Amplitude 1 and down-sampled data. Outliers are indicated as red crosses and can be observed in case of filters with low performance in signal denoising. The black dashed line represents the value of the reference *β*. Boxplots are not reported in case of unstable filters.

**
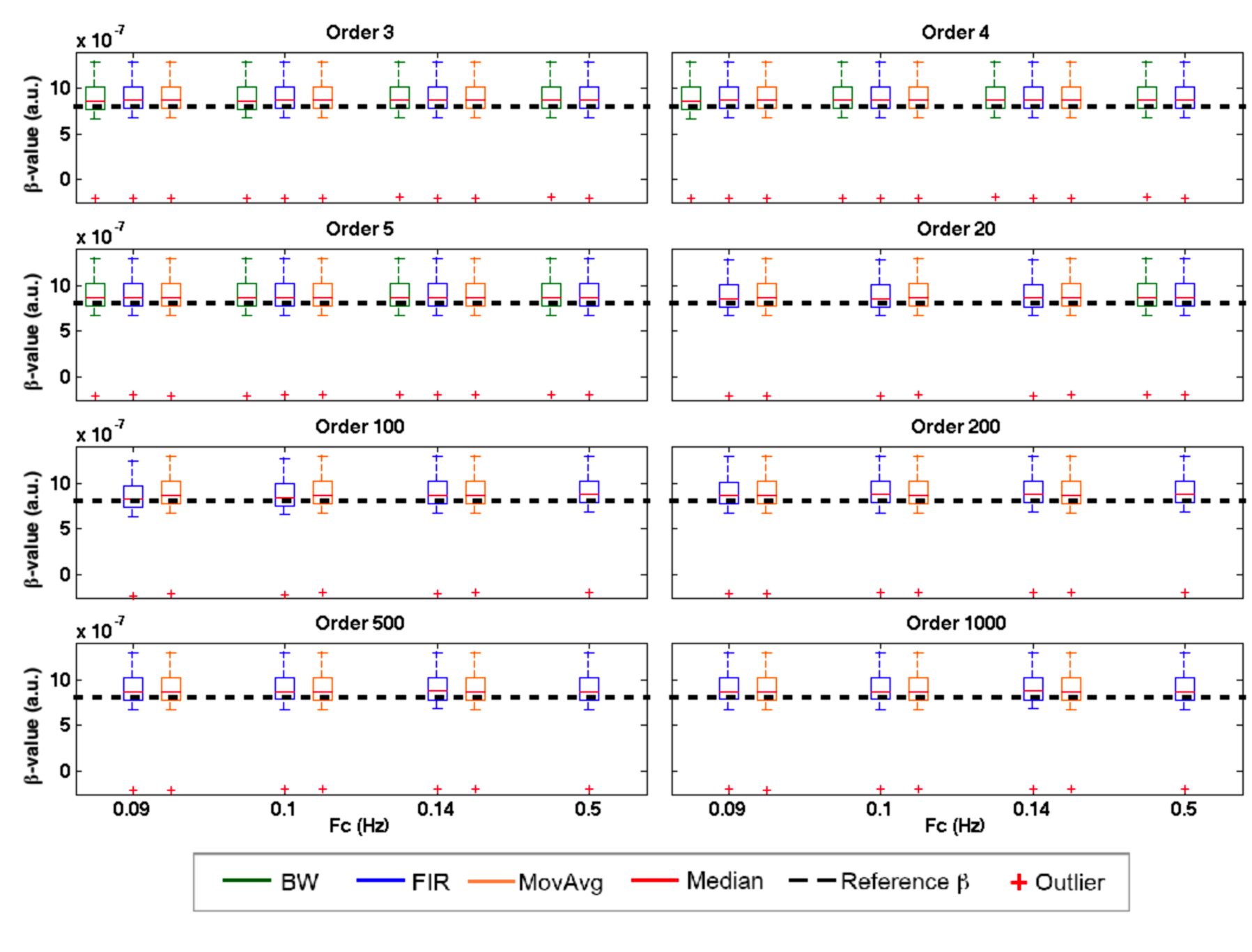
**

**Supplementary figure 7.** Boxplots referring to ΔHbO_2_^C^ LP filtered (green: BW; blue: FIR; orange: MovAvg) data, with Amplitude 1 and down-sampled data. Outliers are indicated as red crosses and can be observed in case of filters with low performance in signal denoising. The black dashed line represents the value of the reference *β*. Boxplots are not reported in case of unstable filters and for *F*_c_ = 0.5 Hz for the MovAvg filter that corresponds to a null window length.

**
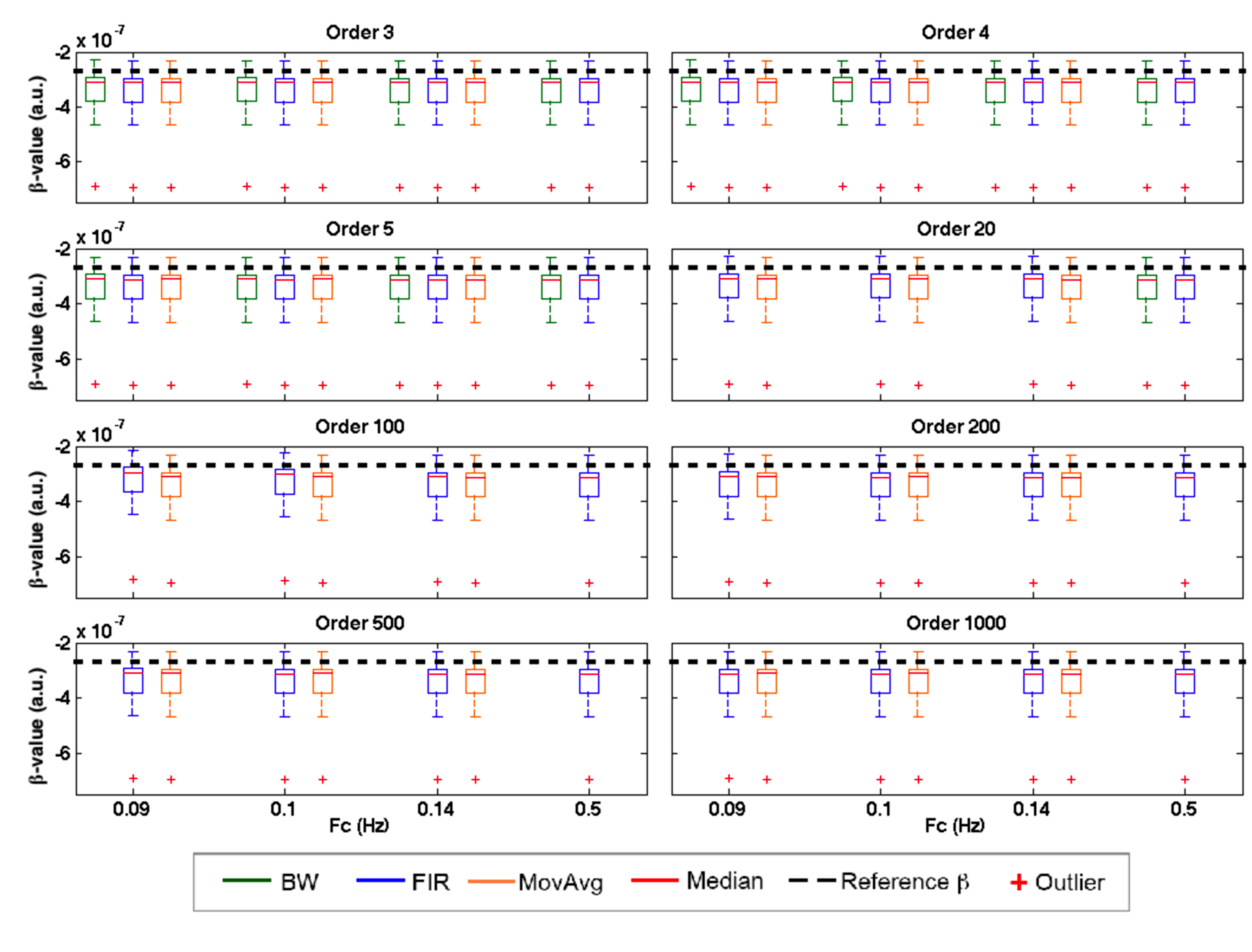
**

**Supplementary figure 8.** Boxplots referring to ΔHbR^C^ LP filtered (green: BW; blue: FIR; orange: MovAvg) data, with Amplitude 1 and down-sampled data. Outliers are indicated as red crosses and can be observed in case of filters with low performance in signal denoising. The black dashed line represents the value of the reference *β*. Boxplots are not reported in case of unstable filters and for *F*_c_ = 0.5 Hz for the MovAvg filter that corresponds to a null window length.

**1.1.3. Precoloring method**


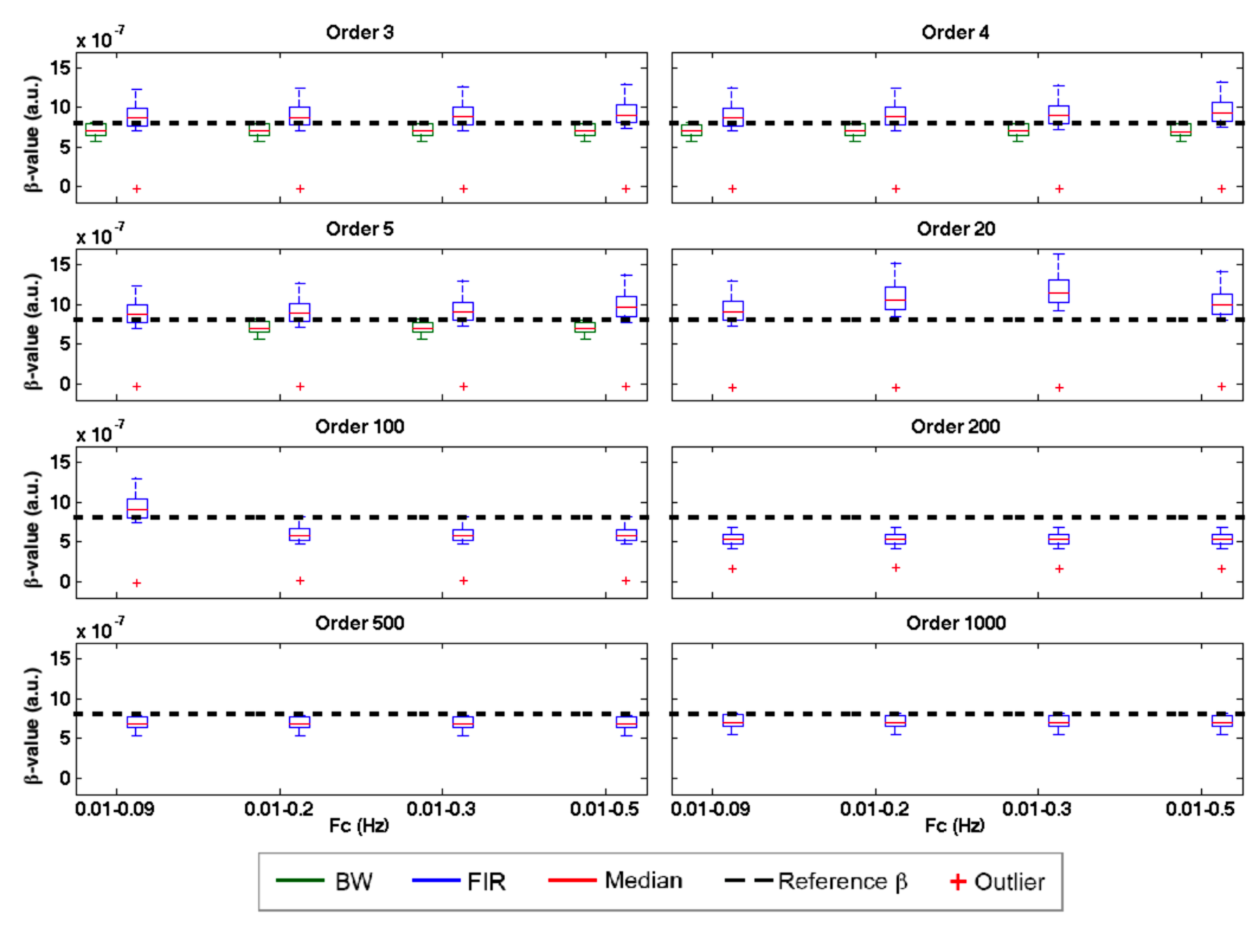


**Supplementary figure 9.** Boxplots referring to ΔHbO_2_^C^ BP filtered (green: BW; blue: FIR) data, with Amplitude 1 and the precoloring method. Outliers are indicated as red crosses and can be observed in case of filters with low performance in signal denoising. The black dashed line represents the value of the reference *β*. Boxplots are not reported in case of unstable filters.

**
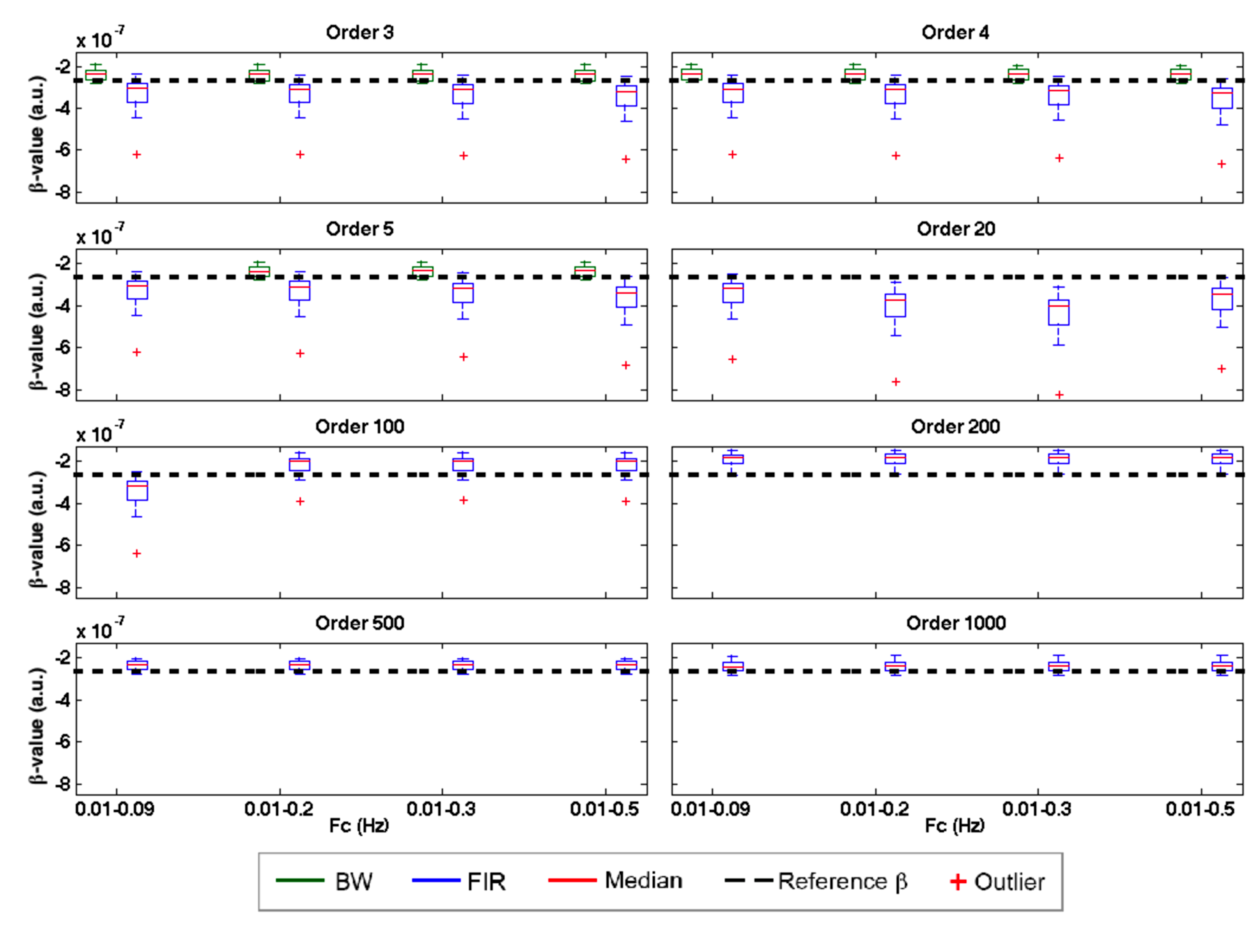
**

**Supplementary figure 10.** Boxplots referring to ΔHbR^C^ BP filtered (green: BW; blue: FIR) data, with Amplitude 1 and the precoloring method. Outliers are indicated as red crosses and can be observed in case of filters with low performance in signal denoising. The black dashed line represents the value of the reference *β*. Boxplots are not reported in case of unstable filters.

**
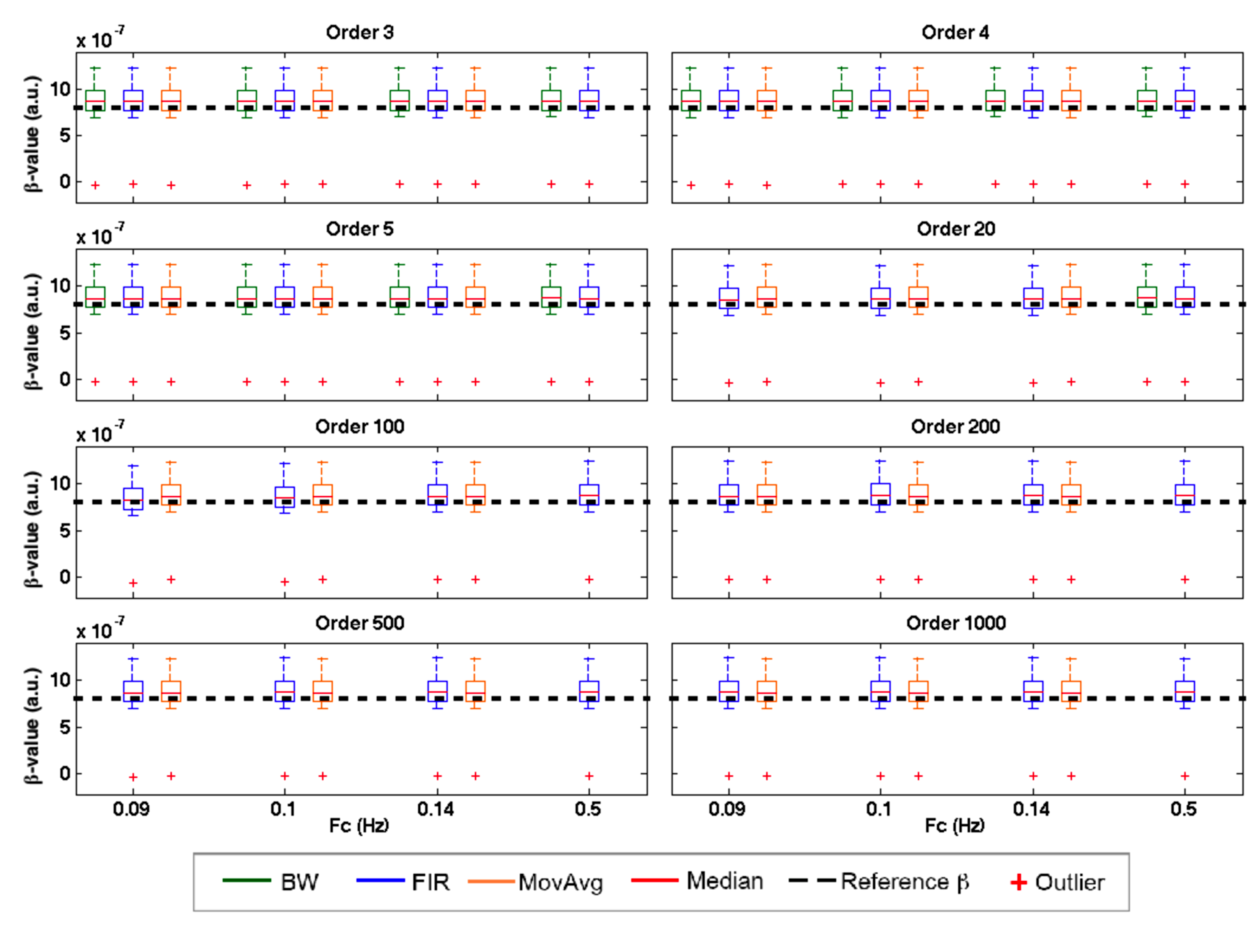
**

**Supplementary figure 11.** Boxplots referring to ΔHbO_2_^C^ LP filtered (green: BW; blue: FIR; orange: MovAvg) data, with Amplitude 1 and the precoloring method. Outliers are indicated as red crosses and can be observed in case of filters with low performance in signal denoising. The black dashed line represents the value of the reference *β*. Boxplots are not reported in case of unstable filters and for *F*_c_ = 0.5 Hz for the MovAvg filter that corresponds to a null window length.

**
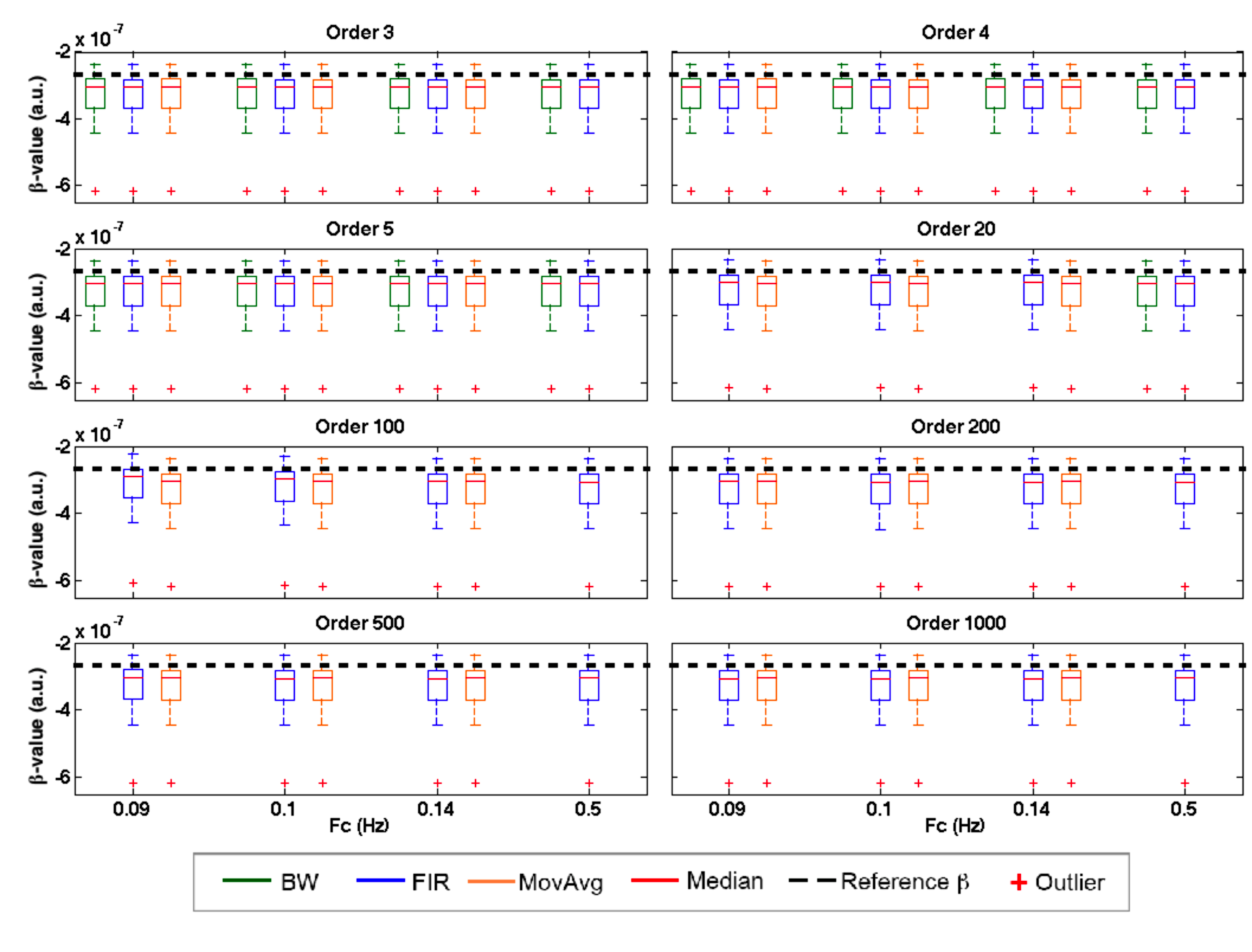
**

**Supplementary figure 12.** Boxplots referring to ΔHbR^C^ LP filtered (green: BW; blue: FIR; orange: MovAvg) data, with Amplitude 1 and the precoloring method. Outliers are indicated as red crosses and can be observed in case of filters with low performance in signal denoising. The black dashed line represents the value of the reference *β*. Boxplots are not reported in case of unstable filters and for *F*_c_ = 0.5 Hz for the MovAvg filter that corresponds to a null window length.

**1.2. Boxplots amplitude 2**

**1.2.1. No correction for serial autocorrelations**


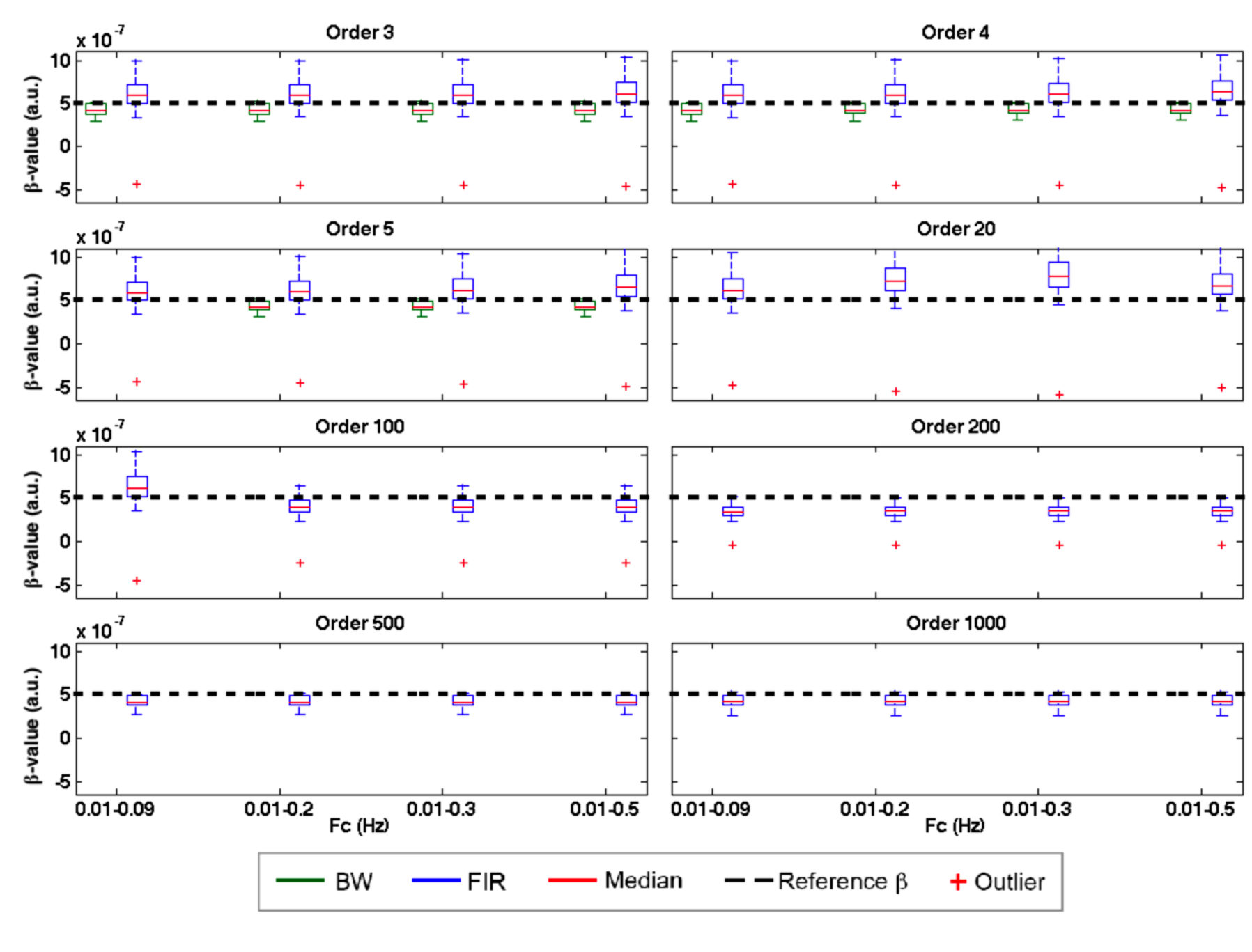


**Supplementary figure 13.** Boxplots referring to ΔHbO_2_^C^ BP filtered (green: BW; blue: FIR) data, with Amplitude 2 and no autocorrelation correction. Outliers are indicated as red crosses and can be observed in case of filters with low performance in signal denoising. The black dashed line represents the value of the reference *β*. Boxplots are not reported in case of unstable filters.

**
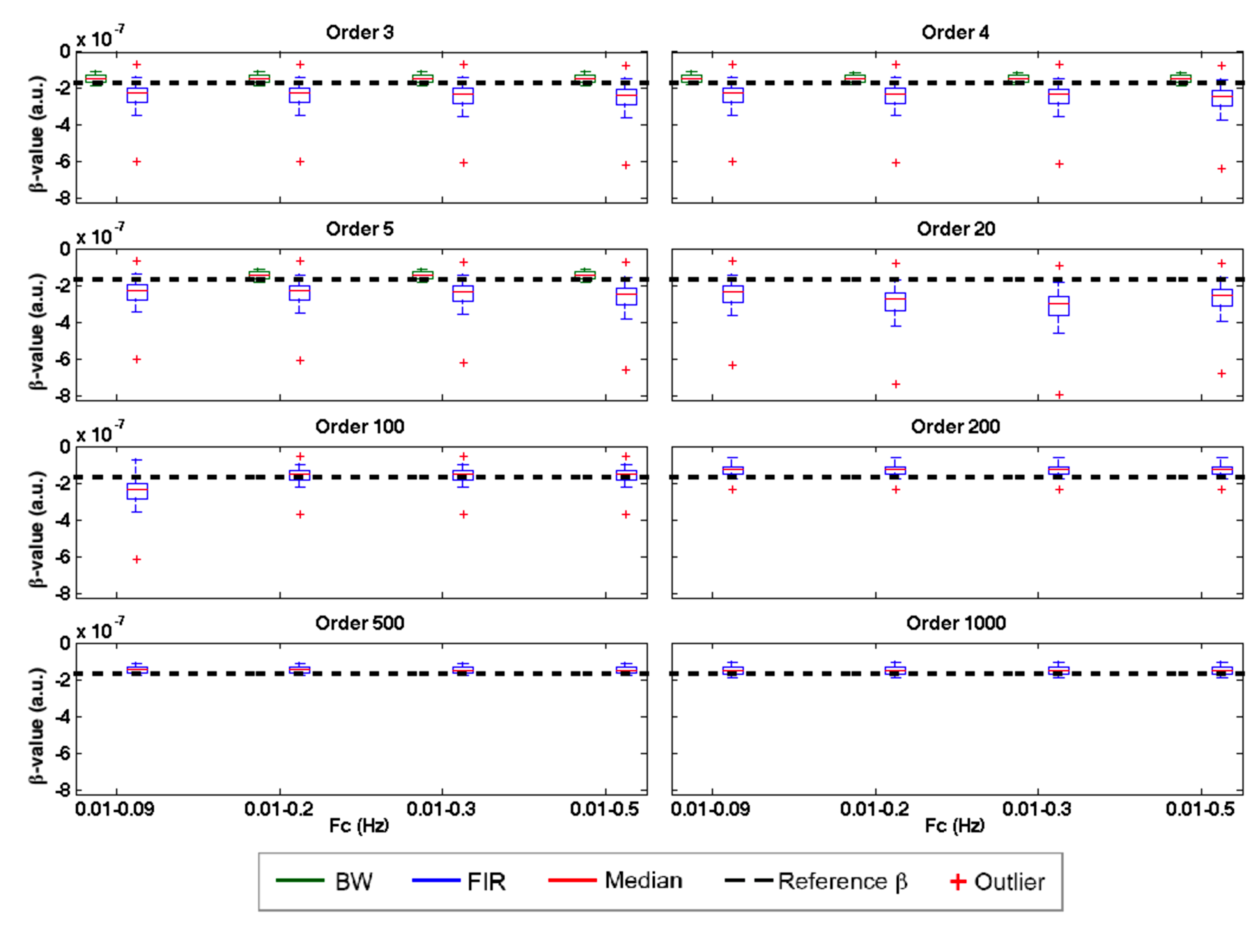
**

**Supplementary figure 14.** Boxplots referring to ΔHbR^C^ BP filtered (green: BW; blue: FIR) data, with Amplitude 2 and no autocorrelation correction. Outliers are indicated as red crosses and can be observed in case of filters with low performance in signal denoising. The black dashed line represents the value of the reference *β*. Boxplots are not reported in case of unstable filters.

**
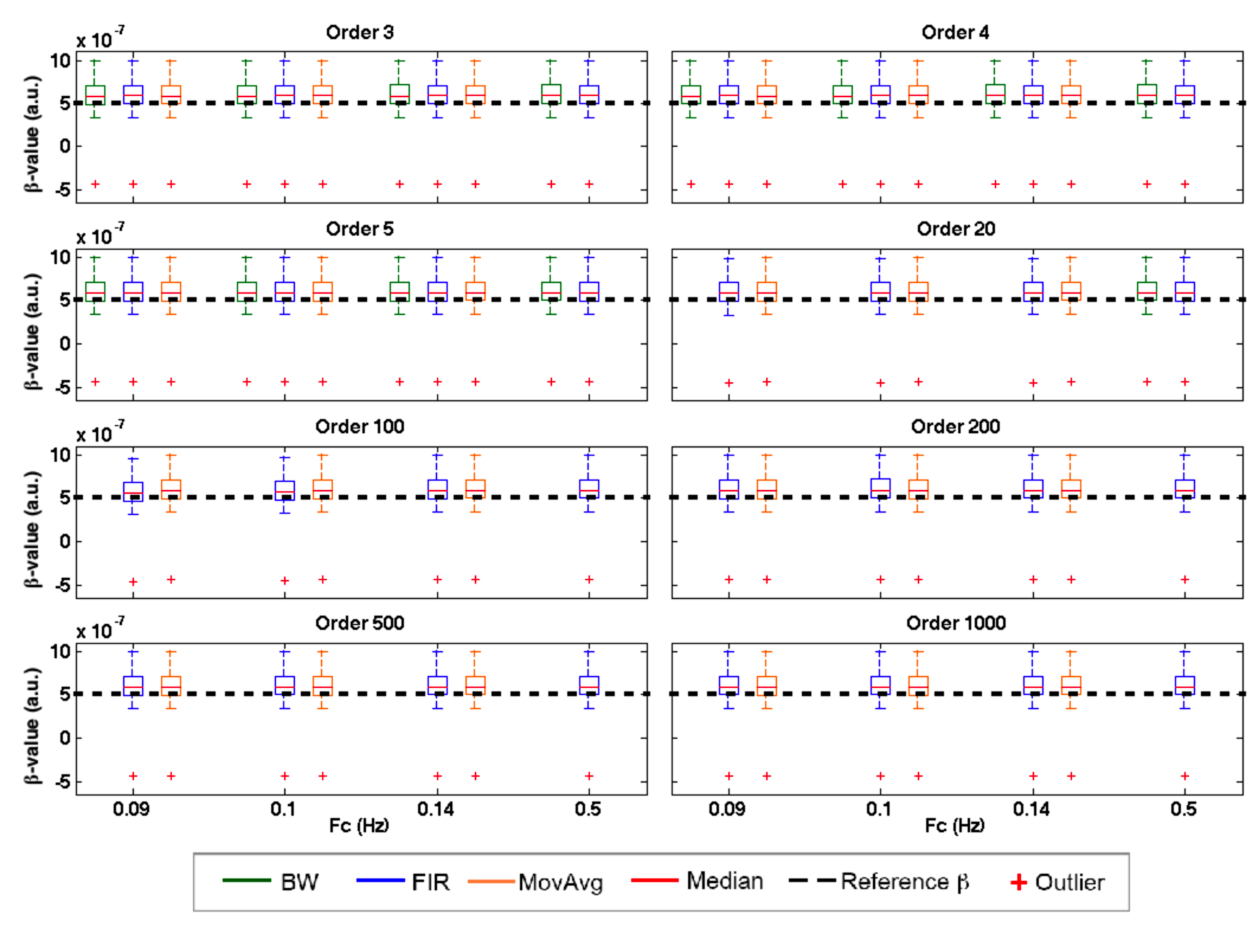
**

**Supplementary figure 15.** Boxplots referring to ΔHbO_2_^C^ LP filtered (green: BW; blue: FIR; orange: MovAvg) data, with Amplitude 2 and no autocorrelation correction. Outliers are indicated as red crosses and can be observed in case of filters with low performance in signal denoising. The black dashed line represents the value of the reference *β*. Boxplots are not reported in case of unstable filters and for *F*_c_ = 0.5 Hz for the MovAvg filter that corresponds to a null window length.

**
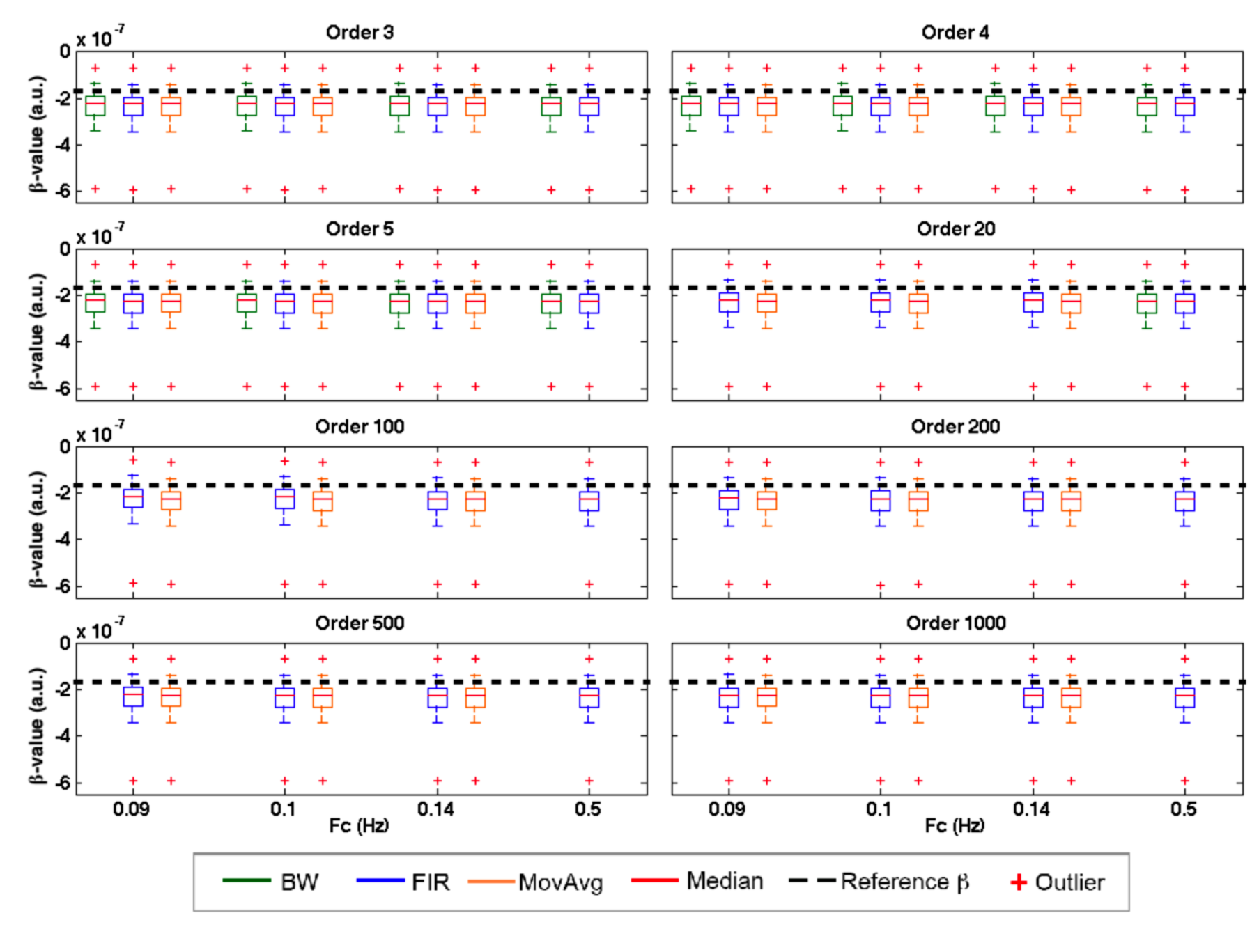
**

**Supplementary figure 16.** Boxplots referring to ΔHbR^C^ LP filtered (green: BW; blue: FIR; orange: MovAvg) data, with Amplitude 2 and no autocorrelation correction. Outliers are indicated as red crosses and can be observed in case of filters with low performance in signal denoising. The black dashed line represents the value of the reference *β*. Boxplots are not reported in case of unstable filters and for *F*_c_ = 0.5 Hz for the MovAvg filter that corresponds to a null window length.

**1.2.2. Down-sampled data**


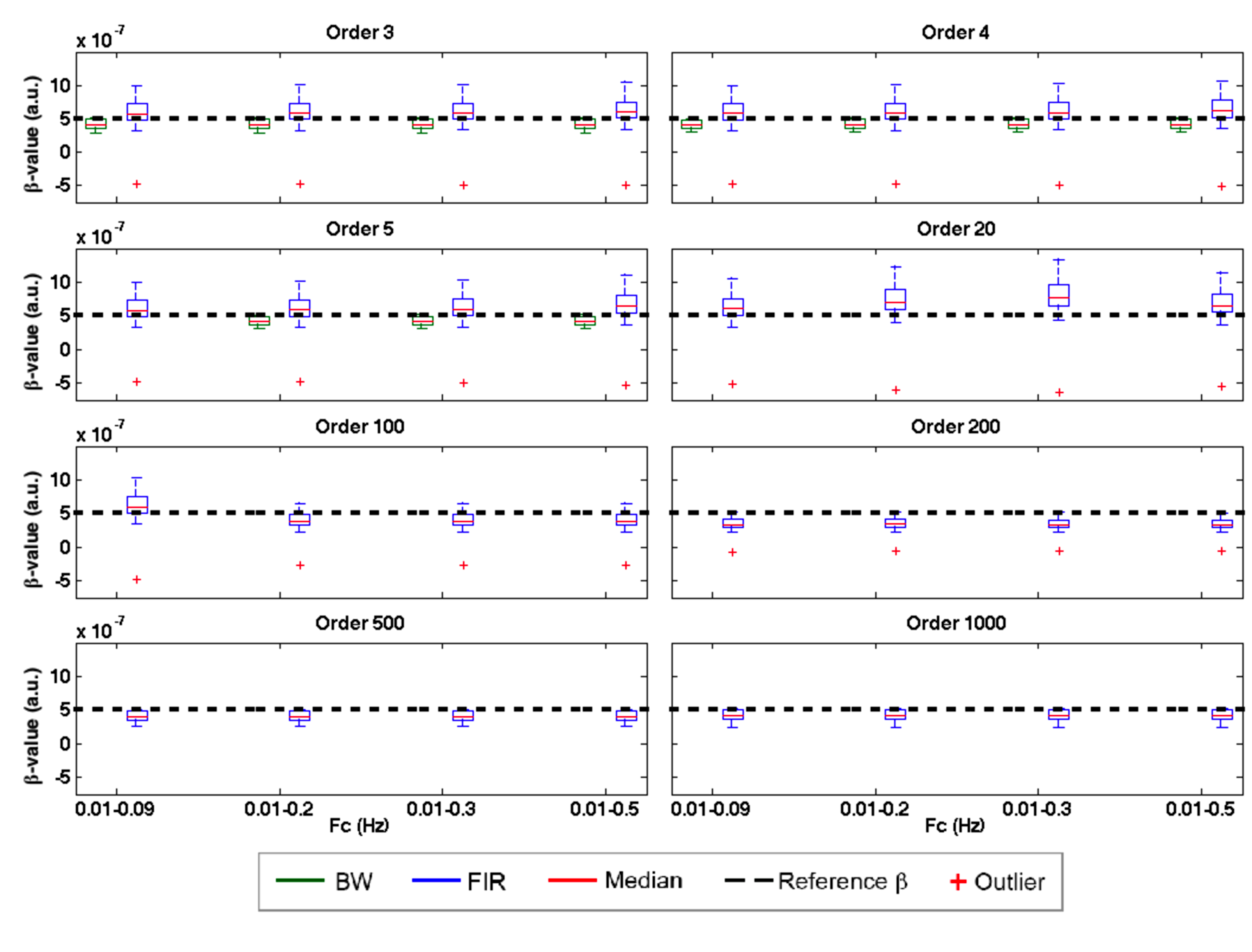


**Supplementary figure 17.** Boxplots referring to ΔHbO_2_^C^ BP filtered (green: BW; blue: FIR) data, with Amplitude 2 and down-sampled data. Outliers are indicated as red crosses and can be observed in case of filters with low performance in signal denoising. The black dashed line represents the value of the reference *β*. Boxplots are not reported in case of unstable filters.

**
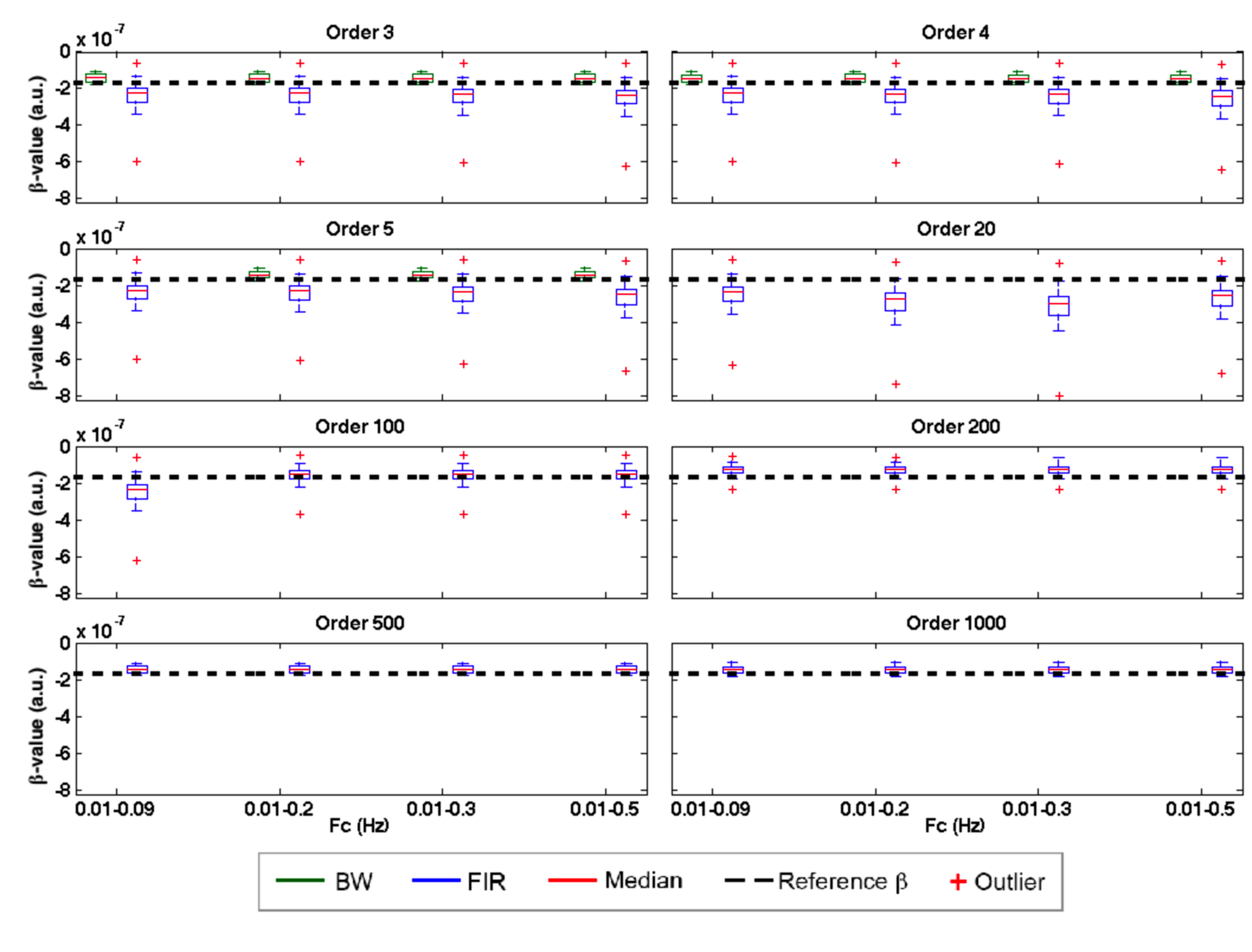
**

**Supplementary figure 18.** Boxplots referring to ΔHbR^C^ BP filtered (green: BW; blue: FIR) data, with Amplitude 2 and down-sampled data. Outliers are indicated as red crosses and can be observed in case of filters with low performance in signal denoising. The black dashed line represents the value of the reference *β*. Boxplots are not reported in case of unstable filters.

**
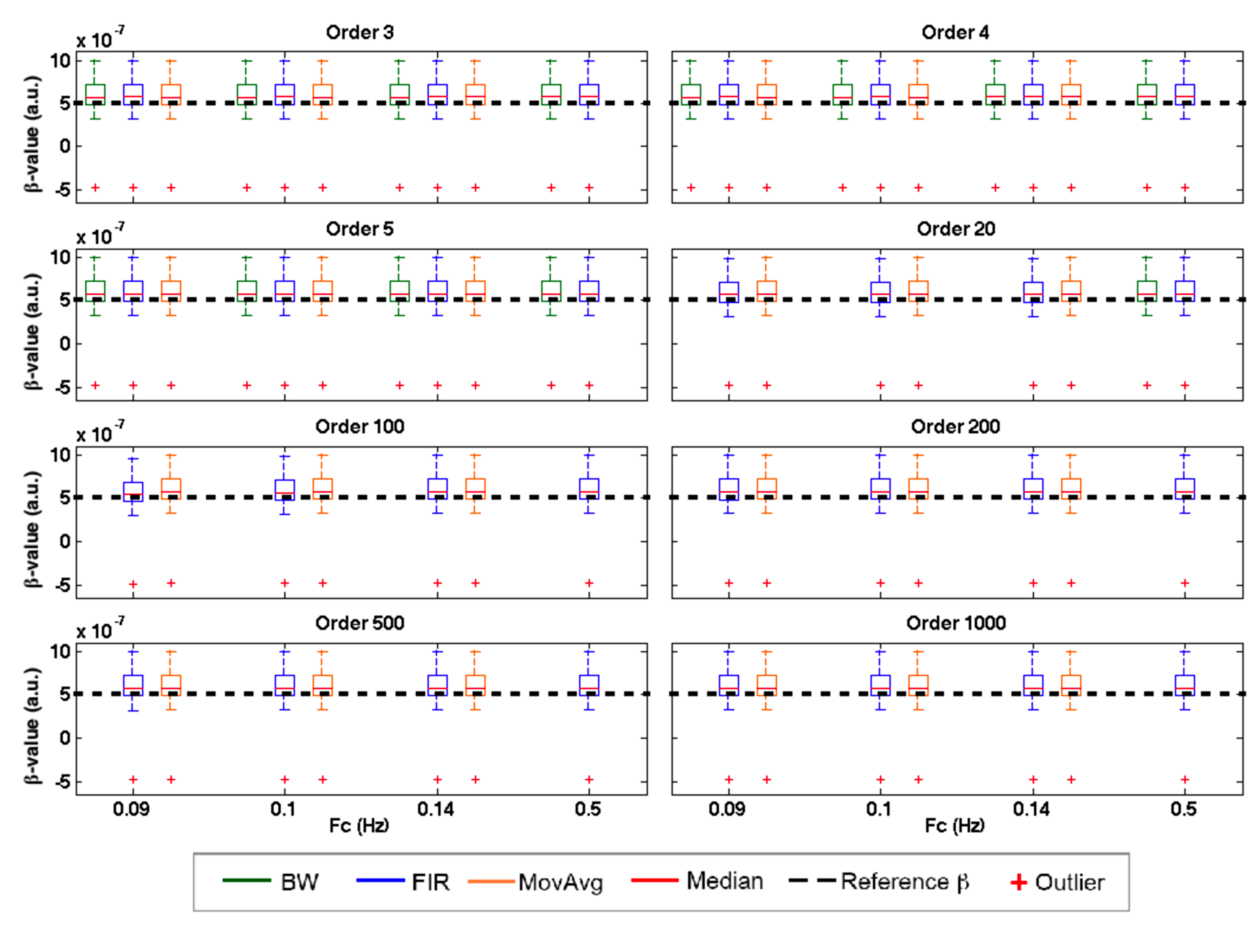
**

**Supplementary figure 19.** Boxplots referring to ΔHbO_2_^C^ LP filtered (green: BW; blue: FIR; orange: MovAvg) data, with Amplitude 2 and down-sampled data. Outliers are indicated as red crosses and can be observed in case of filters with low performance in signal denoising. The black dashed line represents the value of the reference *β*. Boxplots are not reported in case of unstable filters and for *F*_c_ = 0.5 Hz for the MovAvg filter that corresponds to a null window length.

**
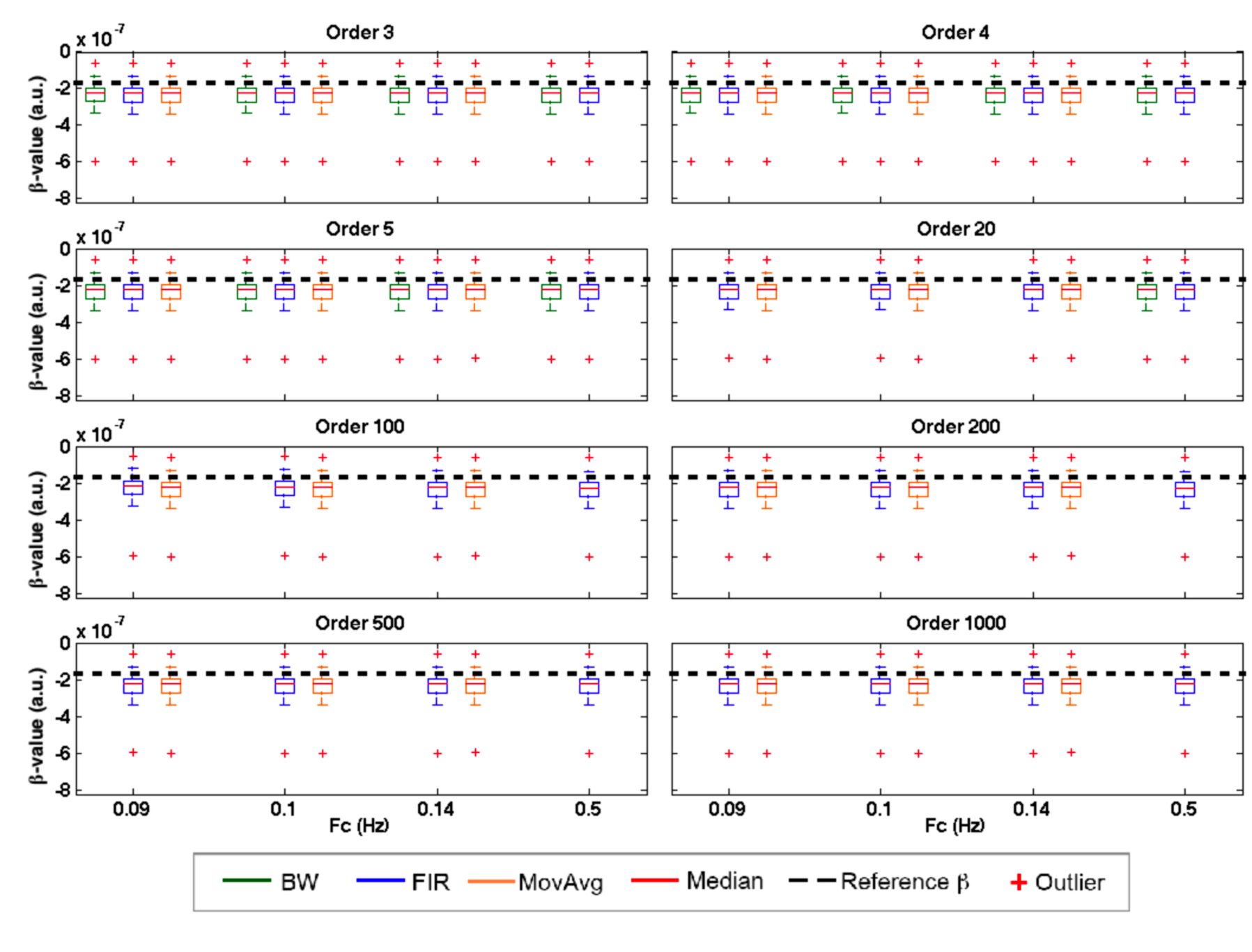
**

**Supplementary figure 20.** Boxplots referring to ΔHbR^C^ LP filtered (green: BW; blue: FIR; orange: MovAvg) data, with Amplitude 2 and down-sampled data. Outliers are indicated as red crosses and can be observed in case of filters with low performance in signal denoising. The black dashed line represents the value of the reference *β*. Boxplots are not reported in case of unstable filters and for *F*_c_ = 0.5 Hz for the MovAvg filter that corresponds to a null window length.

**1.2.3. Precoloring method**


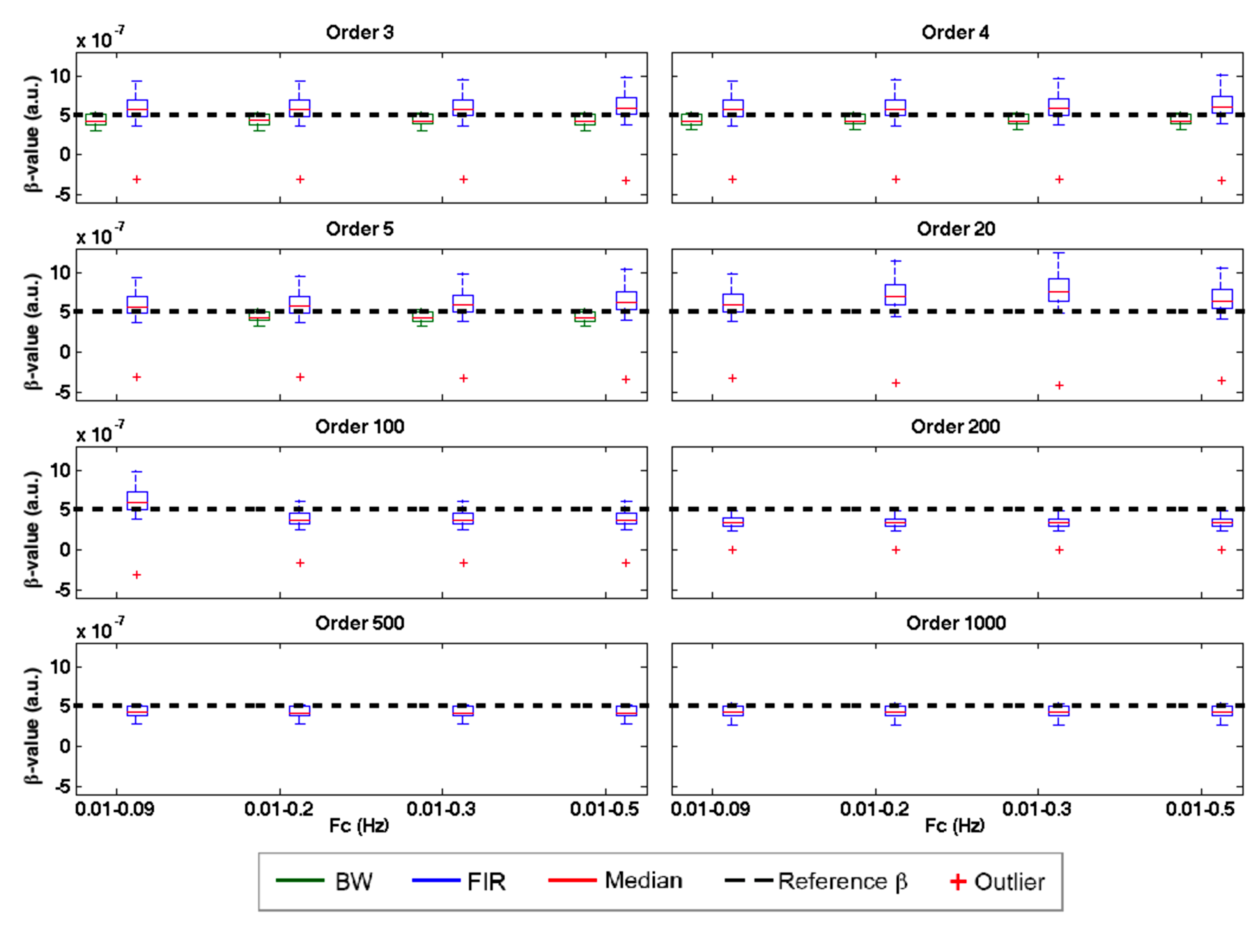


**Supplementary figure 21.** Boxplots referring to ΔHbO_2_^C^ BP filtered (green: BW; blue: FIR) data, with Amplitude 2 and the precoloring method. Outliers are indicated as red crosses and can be observed in case of filters with low performance in signal denoising. The black dashed line represents the value of the reference *β*. Boxplots are not reported in case of unstable filters.

**
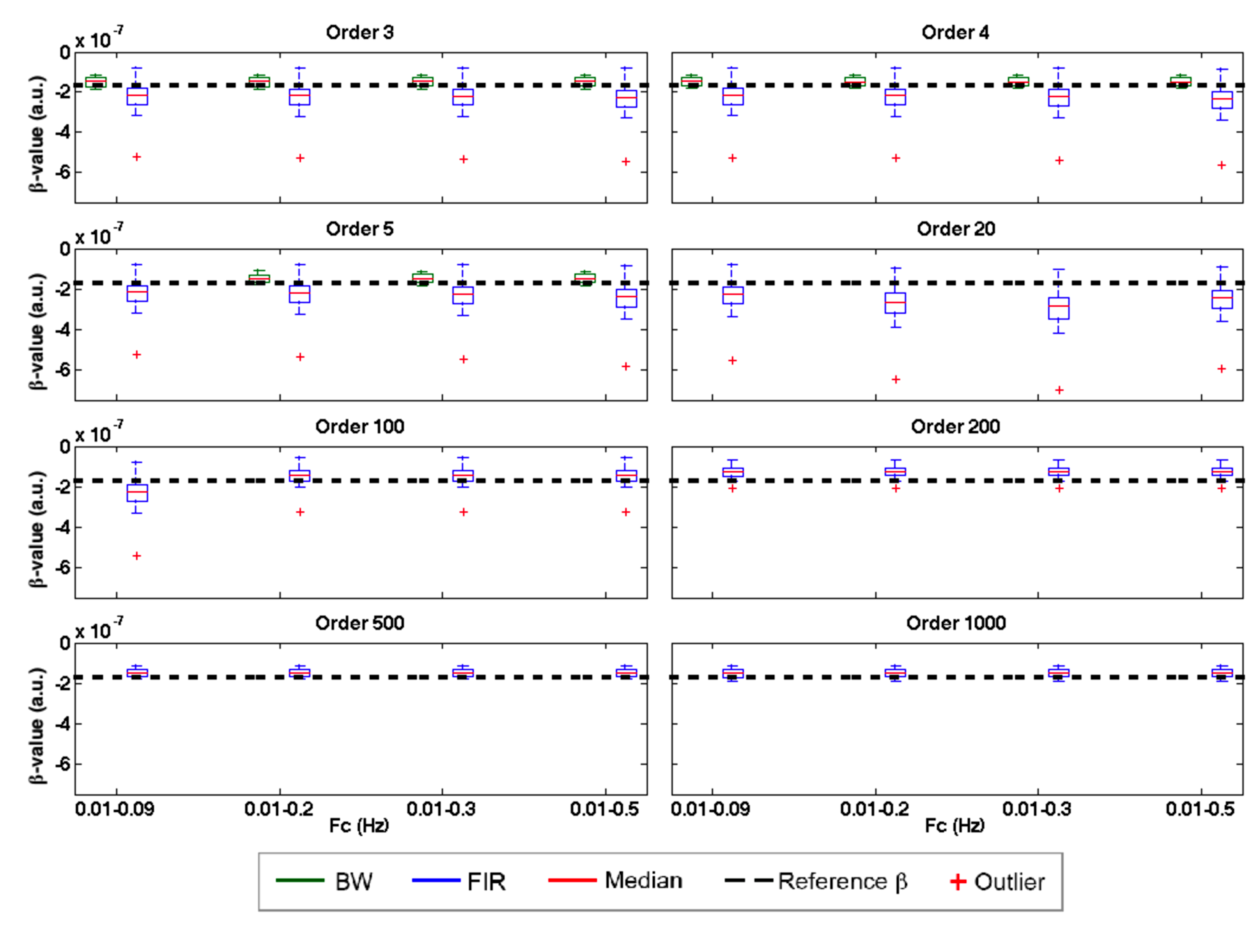
**

**Supplementary figure 22.** Boxplots referring to ΔHbR^C^ BP filtered (green: BW; blue: FIR) data, with Amplitude 2 and the precoloring method. Outliers are indicated as red crosses and can be observed in case of filters with low performance in signal denoising. The black dashed line represents the value of the reference *β*. Boxplots are not reported in case of unstable filters.

**
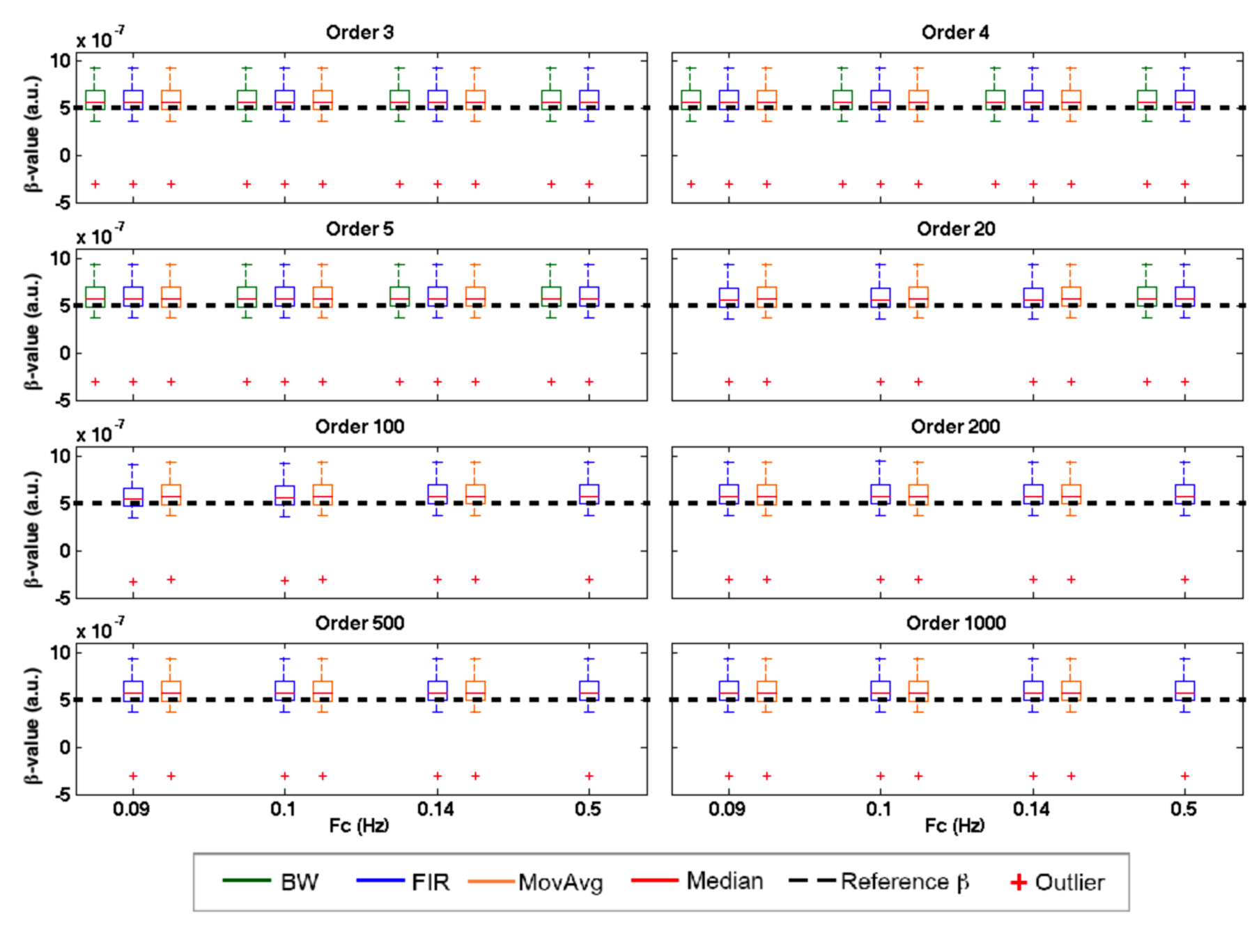
**

**Supplementary figure 23.** Boxplots referring to ΔHbO_2_^C^ LP filtered (green: BW; blue: FIR; orange: MovAvg) data, with Amplitude 2 and the precoloring method. Outliers are indicated as red crosses and can be observed in case of filters with low performance in signal denoising. The black dashed line represents the value of the reference *β*. Boxplots are not reported in case of unstable filters and for *F*_c_ = 0.5 Hz for the MovAvg filter that corresponds to a null window length.

**
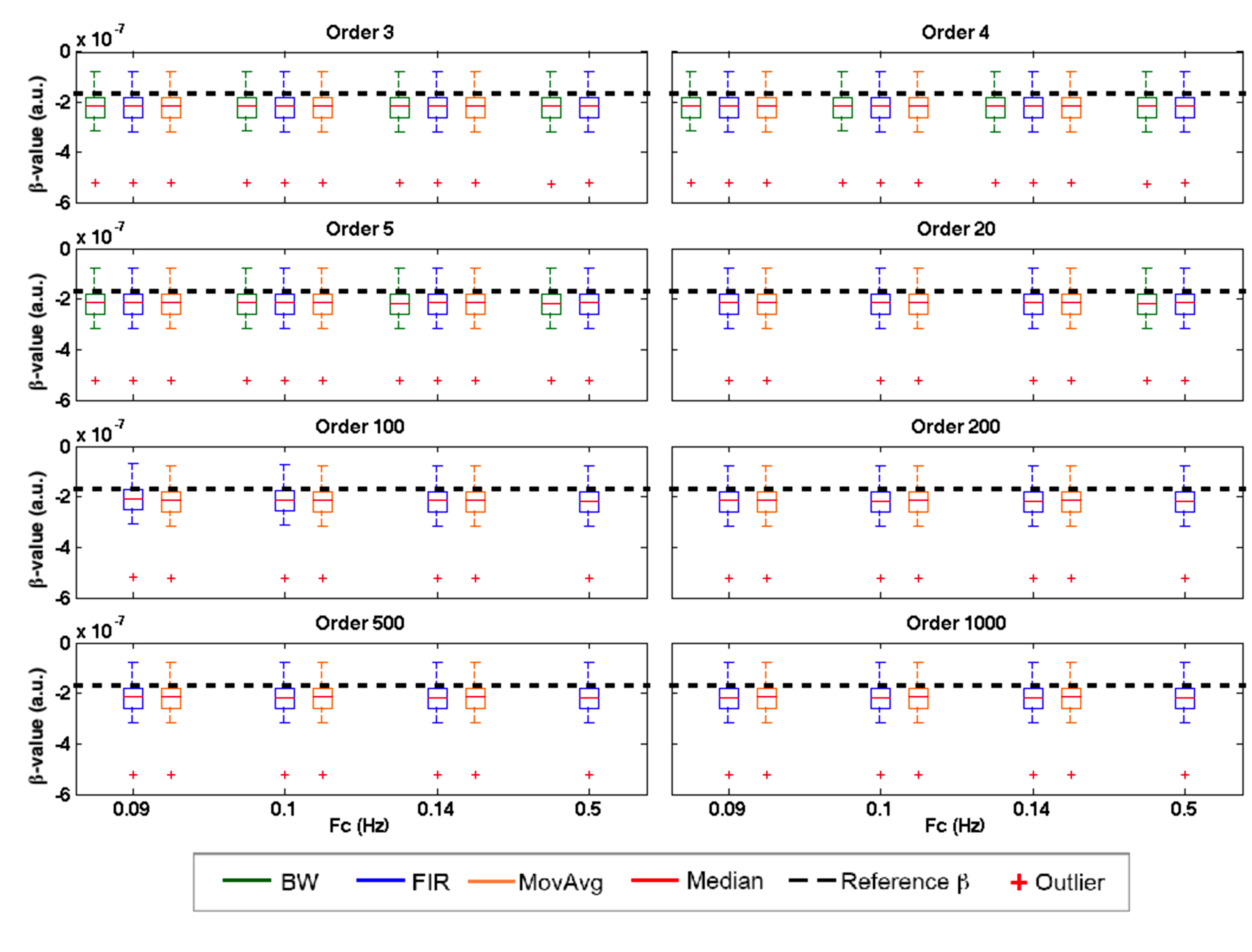
**

**Supplementary figure 24.** Boxplots referring to ΔHbR^C^ LP filtered (green: BW; blue: FIR; orange: MovAvg) data, with Amplitude 2 and the precoloring method. Outliers are indicated as red crosses and can be observed in case of filters with low performance in signal denoising. The black dashed line represents the value of the reference *β*. Boxplots are not reported in case of unstable filters and for *F*_c_ = 0.5 Hz for the MovAvg filter that corresponds to a null window length.

**1.3. Boxplots amplitude 3**

**1.3.1. No correction for serial autocorrelations**


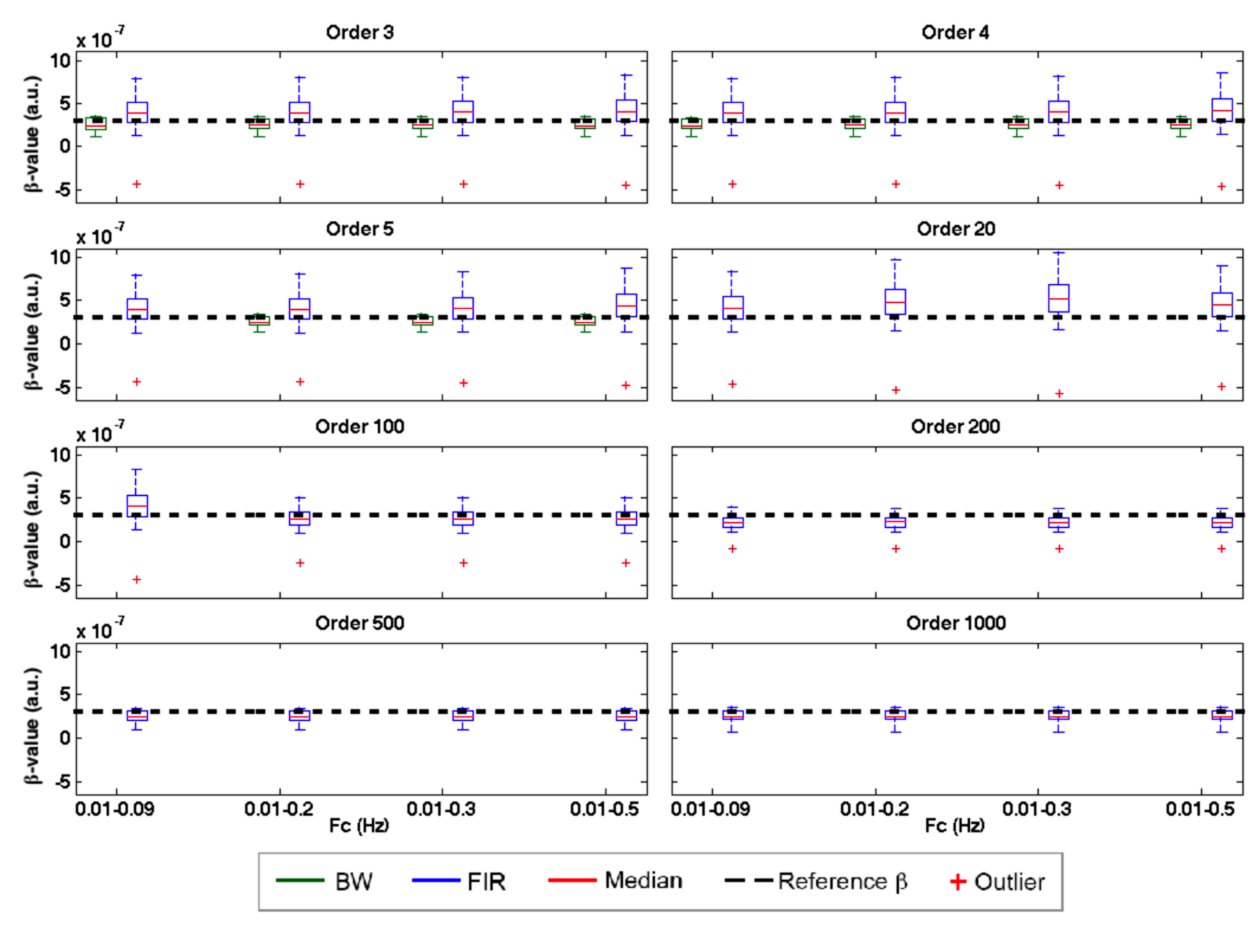


**Supplementary figure 25.** Boxplots referring to ΔHbO_2_^C^ BP filtered (green: BW; blue: FIR) data, with Amplitude 3 and no autocorrelation correction. Outliers are indicated as red crosses and can be observed in case of filters with low performance in signal denoising. The black dashed line represents the value of the reference *β*. Boxplots are not reported in case of unstable filters.

**
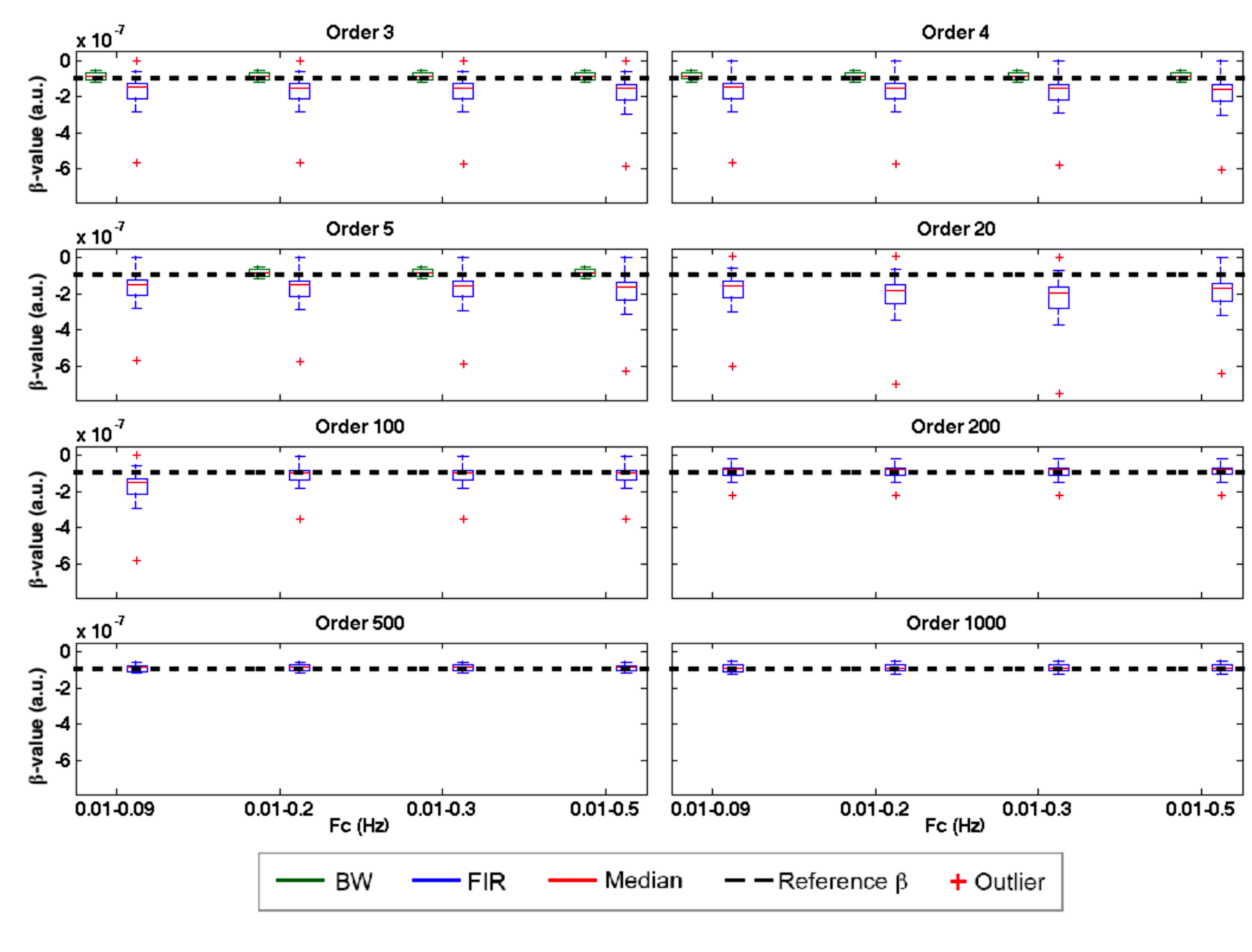
**

**Supplementary figure 26.** Boxplots referring to ΔHbR^C^ BP filtered (green: BW; blue: FIR) data, with Amplitude 3 and no autocorrelation correction. Outliers are indicated as red crosses and can be observed in case of filters with low performance in signal denoising. The black dashed line represents the value of the reference *β*. Boxplots are not reported in case of unstable filters.

**
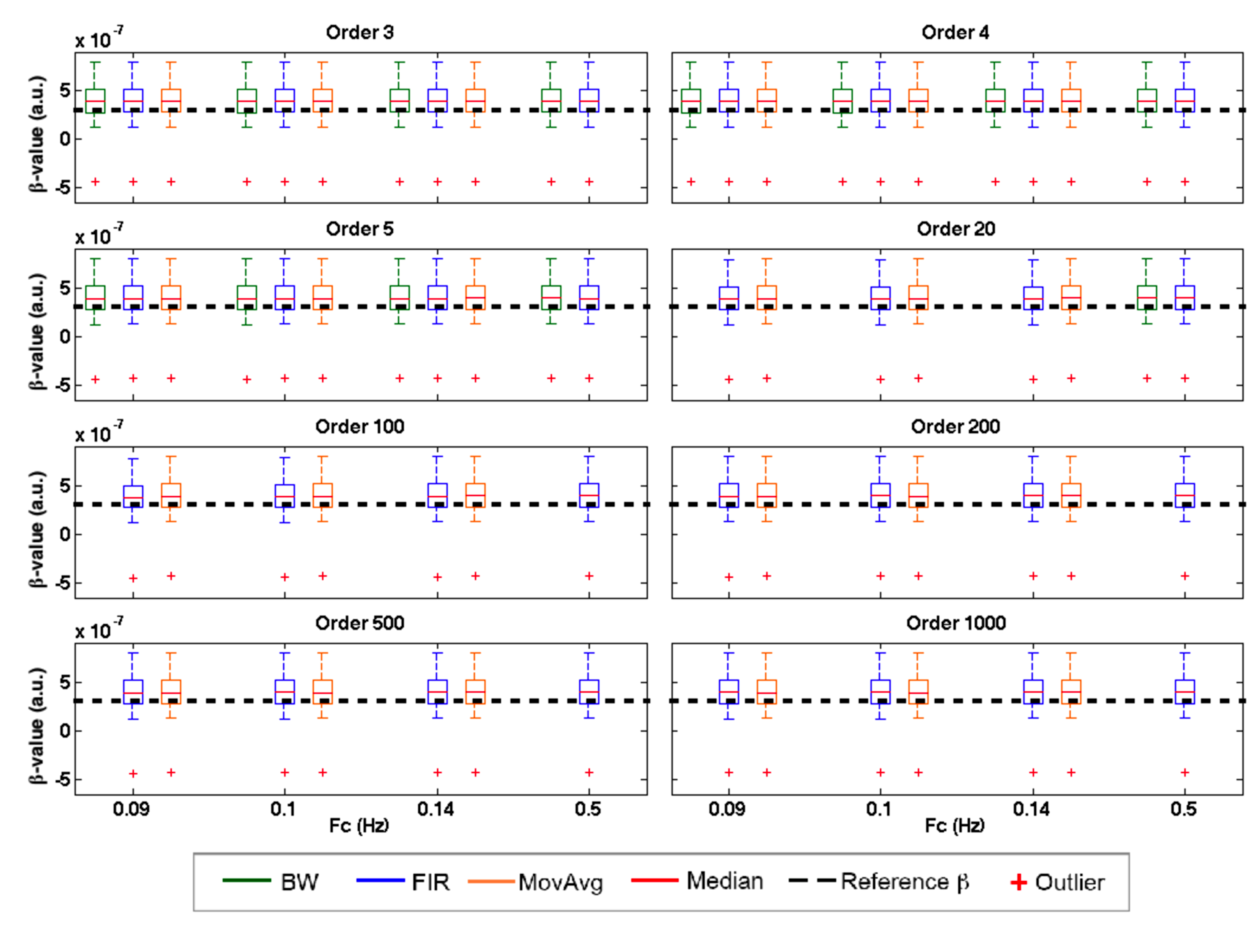
**

**Supplementary figure 27.** Boxplots referring to ΔHbO_2_^C^ LP filtered (green: BW; blue: FIR; orange: MovAvg) data, with Amplitude 3 and no autocorrelation correction. Outliers are indicated as red crosses and can be observed in case of filters with low performance in signal denoising. The black dashed line represents the value of the reference *β*. Boxplots are not reported in case of unstable filters and for *F*_c_ = 0.5 Hz for the MovAvg filter that corresponds to a null window length.

**
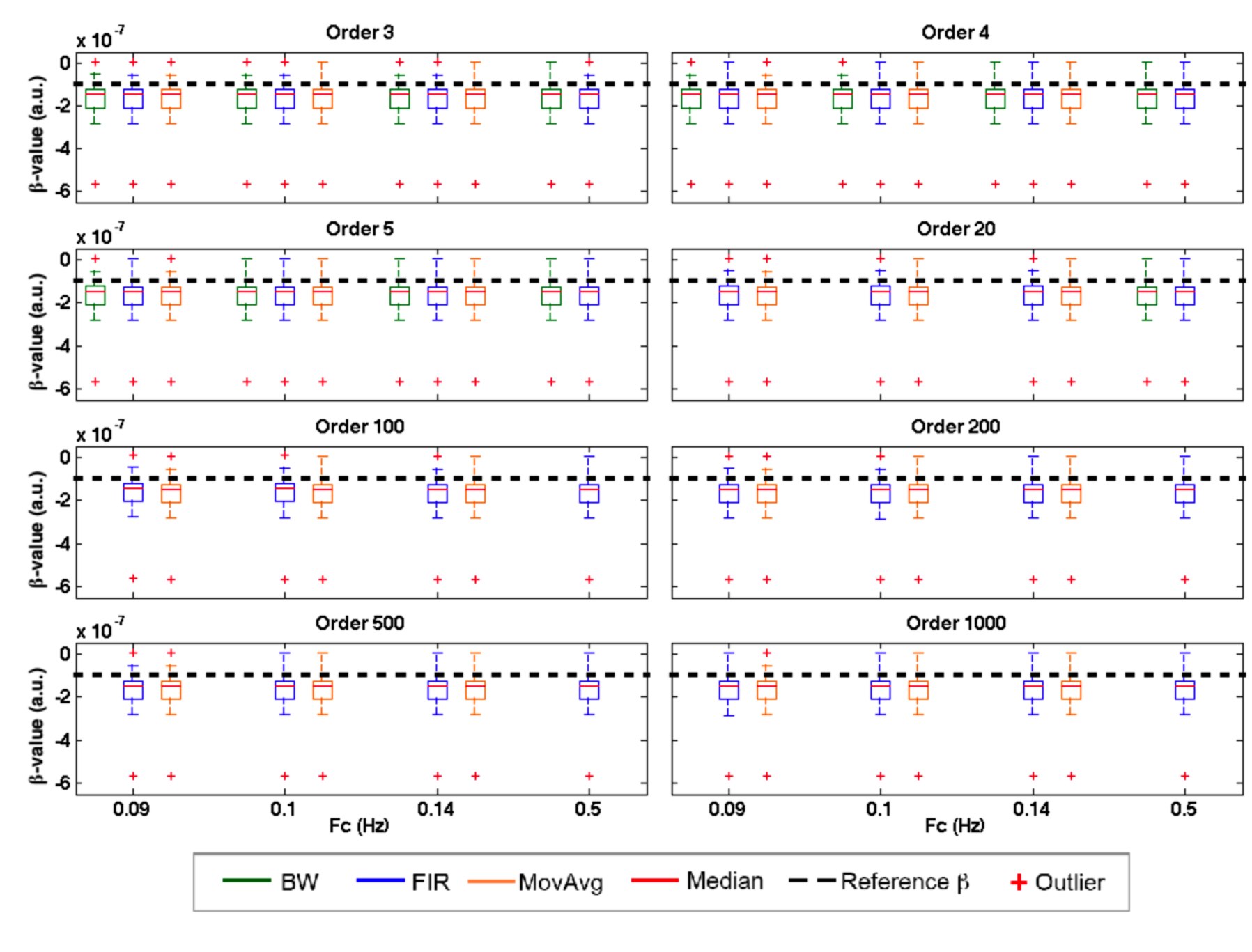
**

**Supplementary figure 28.** Boxplots referring to ΔHbR^C^ LP filtered (green: BW; blue: FIR; orange: MovAvg) data, with Amplitude 3 and no autocorrelation correction. Outliers are indicated as red crosses and can be observed in case of filters with low performance in signal denoising. The black dashed line represents the value of the reference *β*. Boxplots are not reported in case of unstable filters and for *F*_c_ = 0.5 Hz for the MovAvg filter that corresponds to a null window length.

**1.3.2. Down-sampled data**


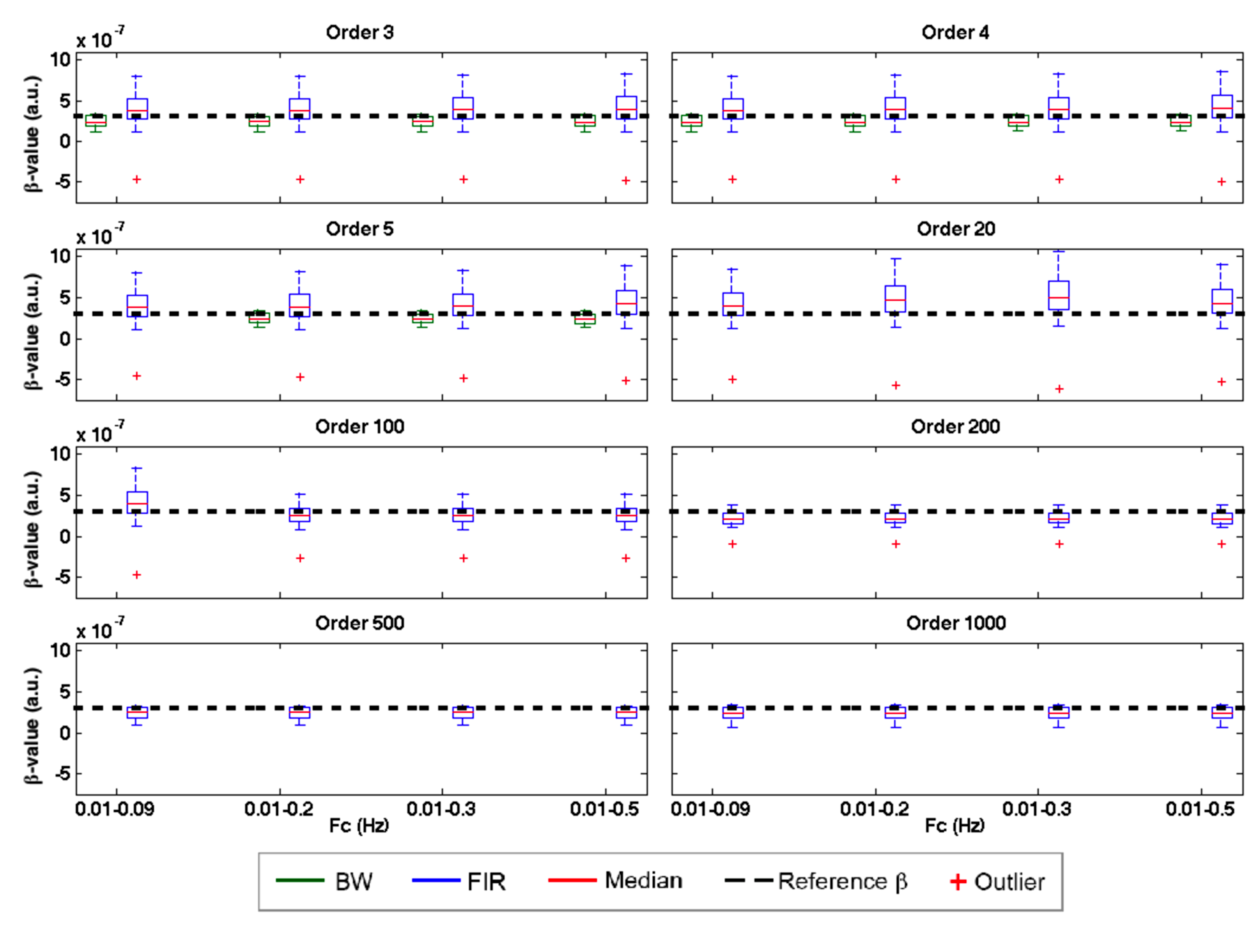


**Supplementary figure 29.** Boxplots referring to ΔHbO_2_^C^ BP filtered (green: BW; blue: FIR) data, with Amplitude 3 and down-sampled data. Outliers are indicated as red crosses and can be observed in case of filters with low performance in signal denoising. The black dashed line represents the value of the reference *β*. Boxplots are not reported in case of unstable filters.

**
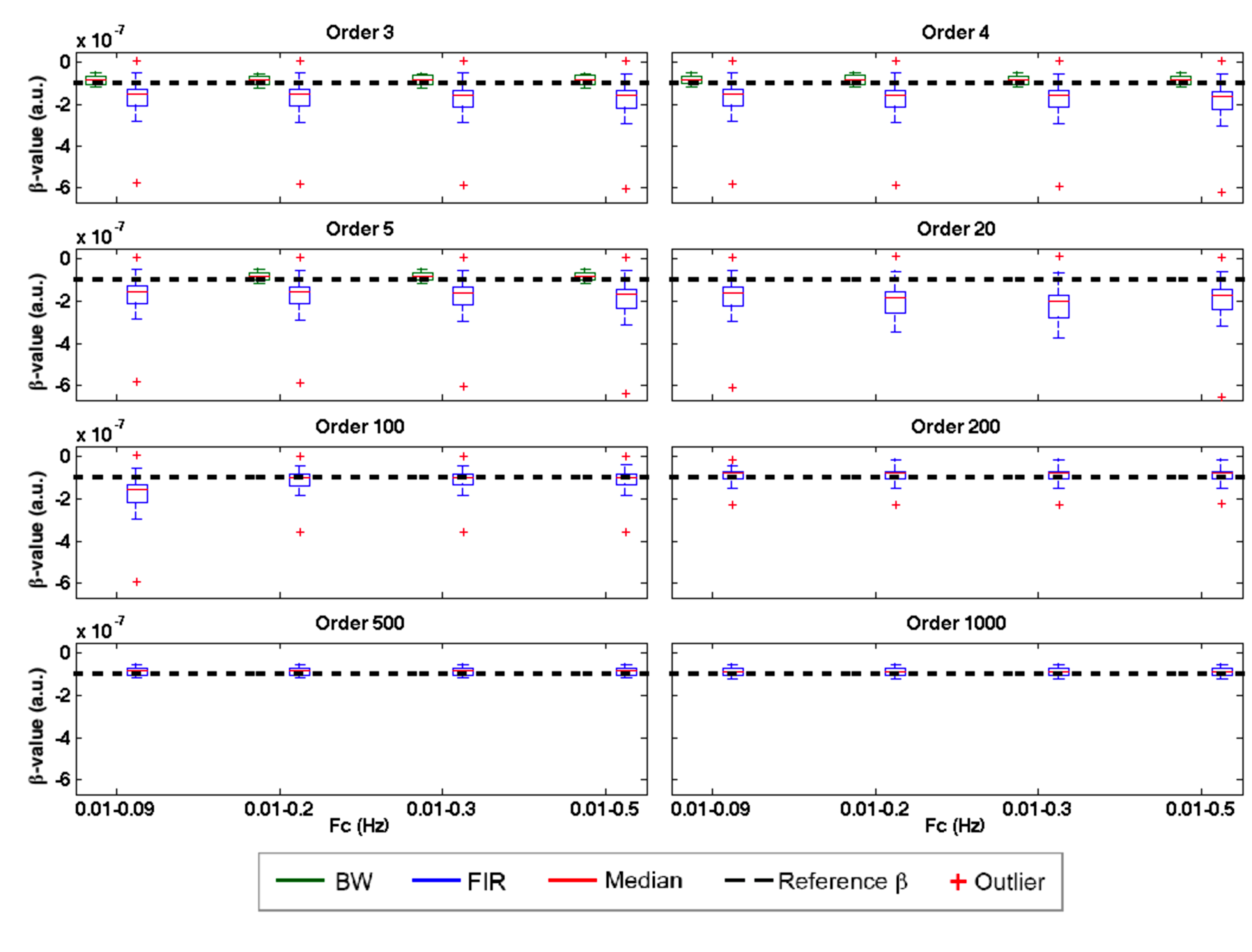
**

**Supplementary figure 30.** Boxplots referring to ΔHbR^C^ BP filtered (green: BW; blue: FIR) data, with Amplitude 3 and down-sampled data. Outliers are indicated as red crosses and can be observed in case of filters with low performance in signal denoising. The black dashed line represents the value of the reference *β*. Boxplots are not reported in case of unstable filters.

**
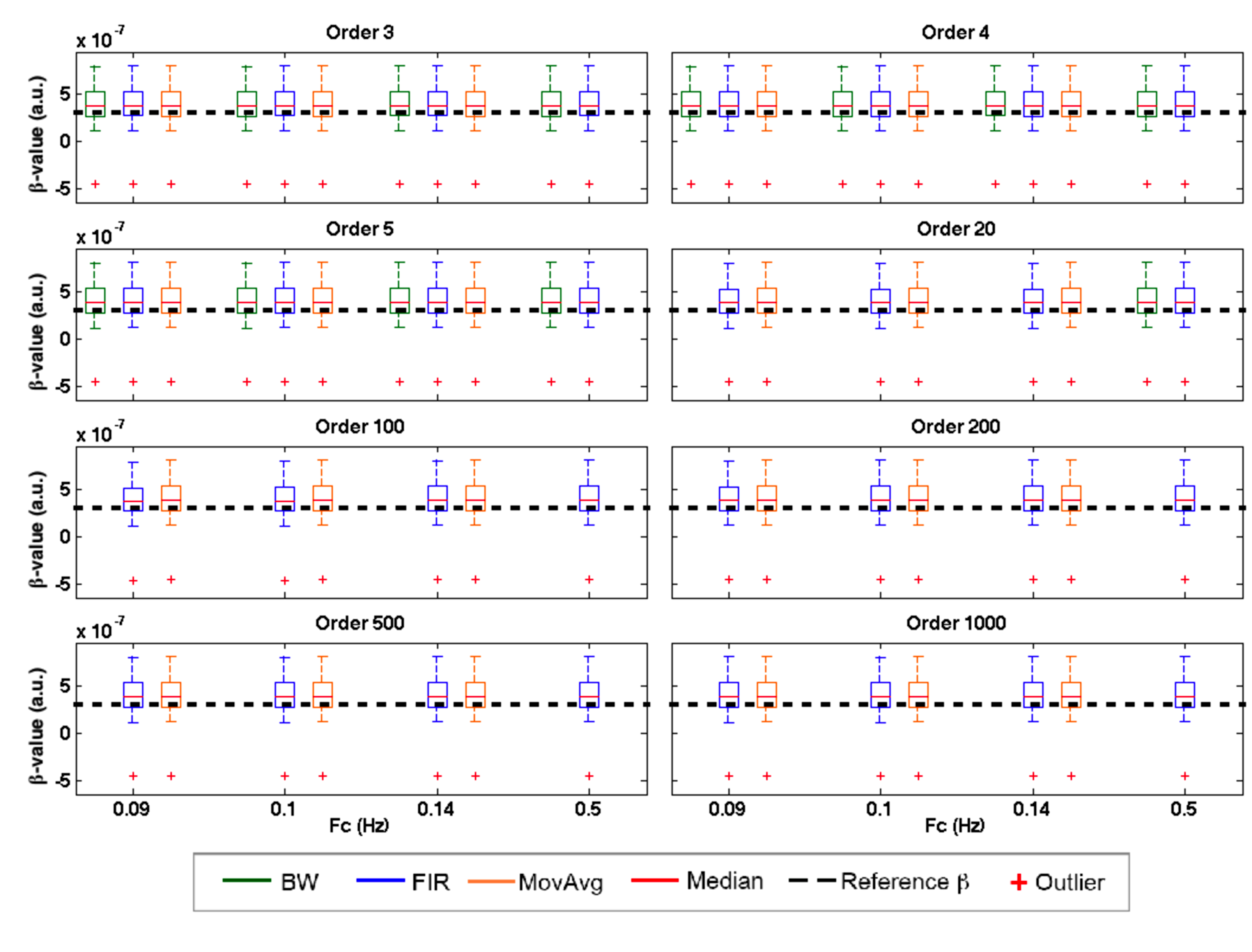
**

**Supplementary figure 31.** Boxplots referring to ΔHbO_2_^C^ LP filtered (green: BW; blue: FIR; orange: MovAvg) data, with Amplitude 3 and down-sampled data. Outliers are indicated as red crosses and can be observed in case of filters with low performance in signal denoising. The black dashed line represents the value of the reference *β*. Boxplots are not reported in case of unstable filters and for *F*_c_ = 0.5 Hz for the MovAvg filter that corresponds to a null window length.

**
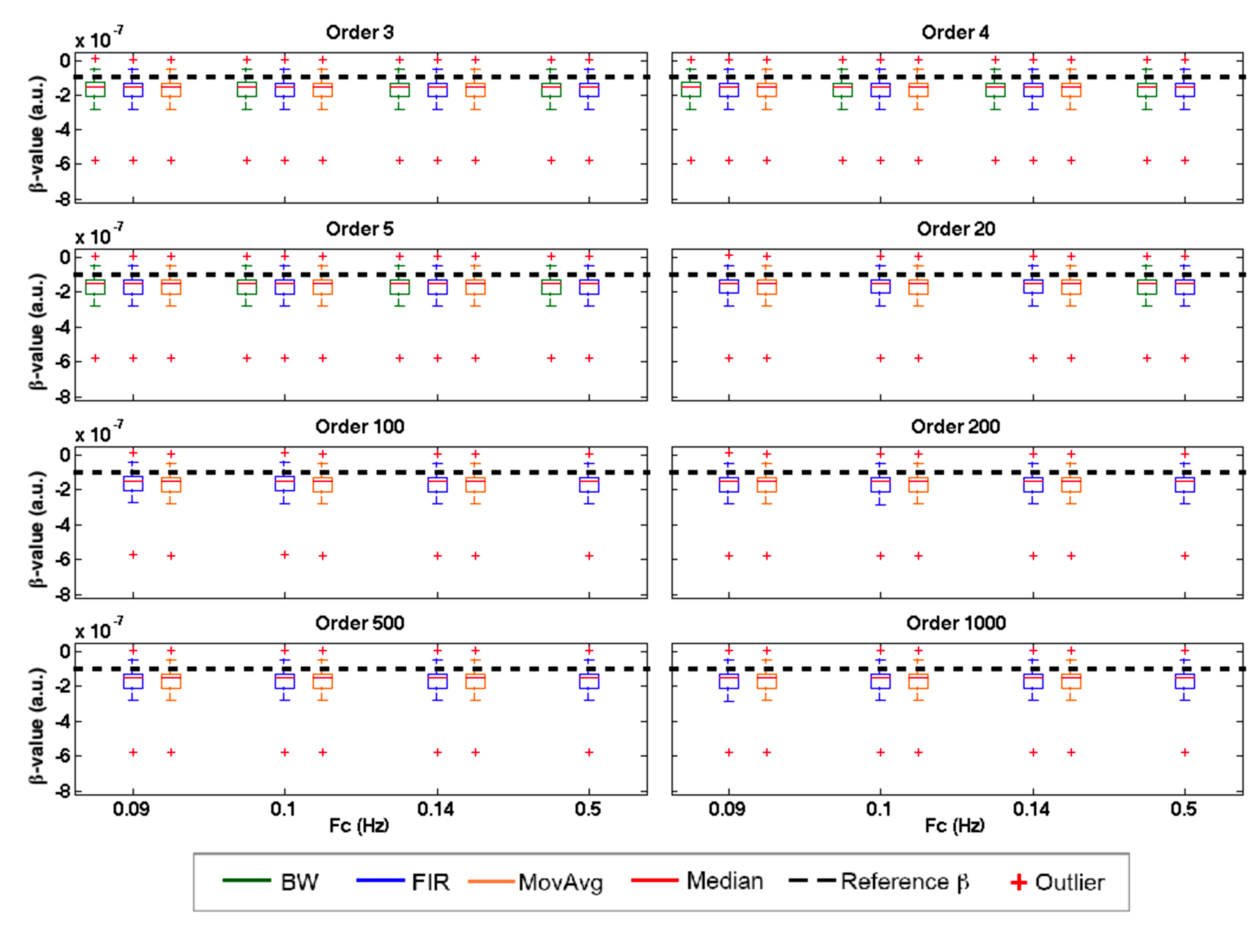
**

**Supplementary figure 32.** Boxplots referring to ΔHbR^C^ LP filtered (green: BW; blue: FIR; orange: MovAvg) data, with Amplitude 3 and down-sampled data. Outliers are indicated as red crosses and can be observed in case of filters with low performance in signal denoising. The black dashed line represents the value of the reference *β*. Boxplots are not reported in case of unstable filters and for *F*_c_ = 0.5 Hz for the MovAvg filter that corresponds to a null window length.

**1.3.3. Precoloring method**


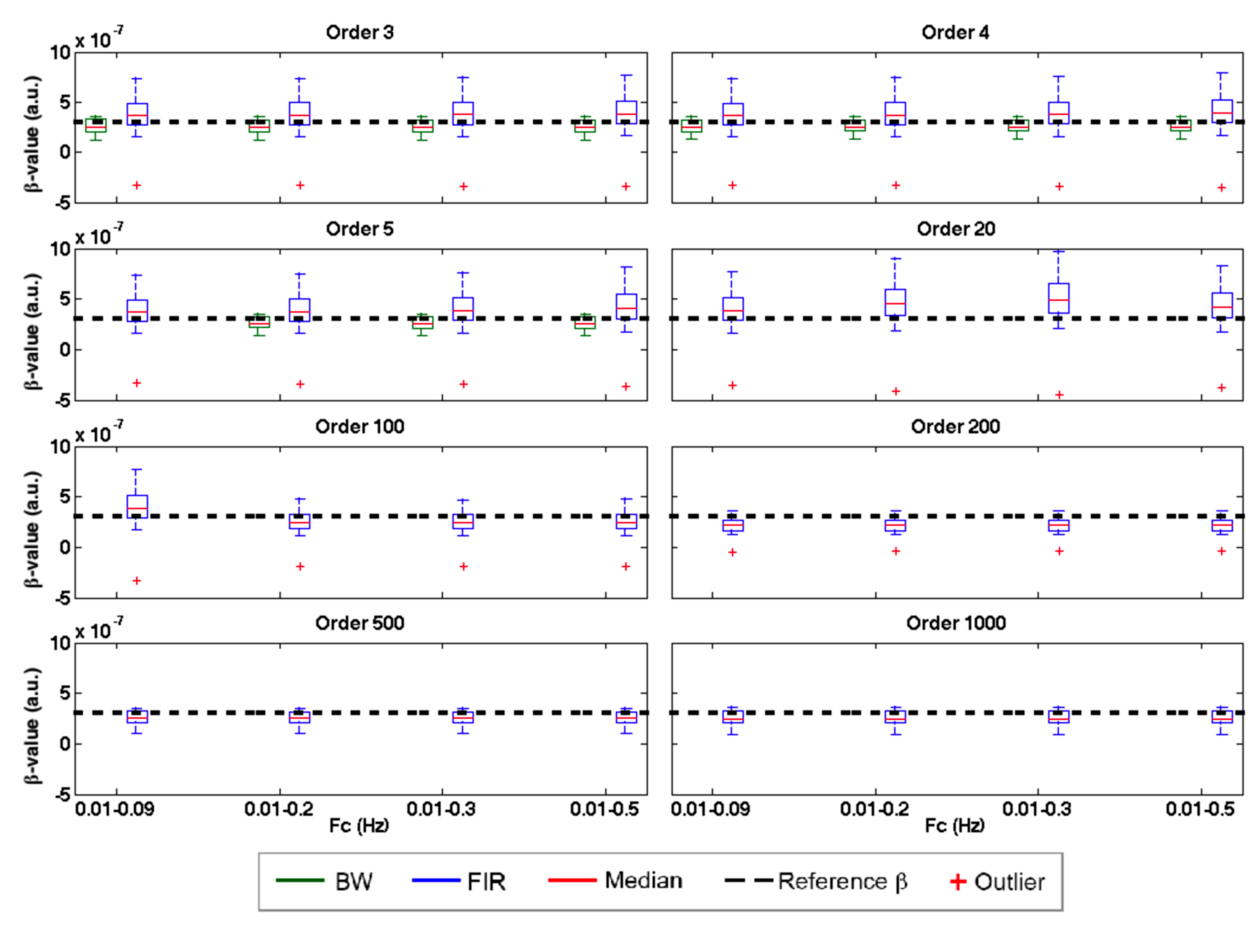


**Supplementary figure 33.** Boxplots referring to ΔHbO_2_^C^ BP filtered (green: BW; blue: FIR) data, with Amplitude 3 and the precoloring method. Outliers are indicated as red crosses and can be observed in case of filters with low performance in signal denoising. The black dashed line represents the value of the reference *β*. Boxplots are not reported in case of unstable filters.

**
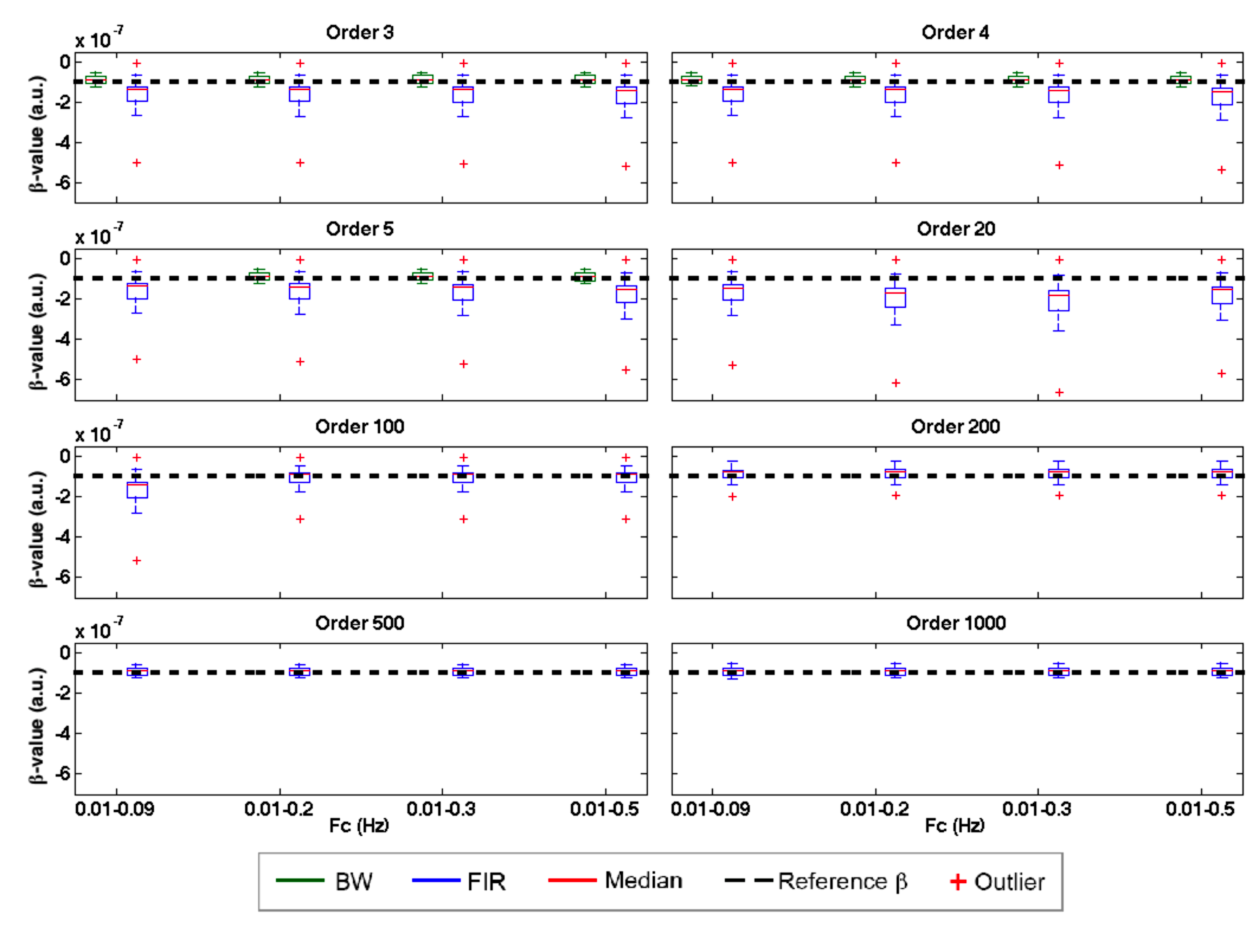
**

**Supplementary figure 34.** Boxplots referring to ΔHbR^C^ BP filtered (green: BW; blue: FIR) data, with Amplitude 3 and the precoloring method. Outliers are indicated as red crosses and can be observed in case of filters with low performance in signal denoising. The black dashed line represents the value of the reference *β*. Boxplots are not reported in case of unstable filters.

**
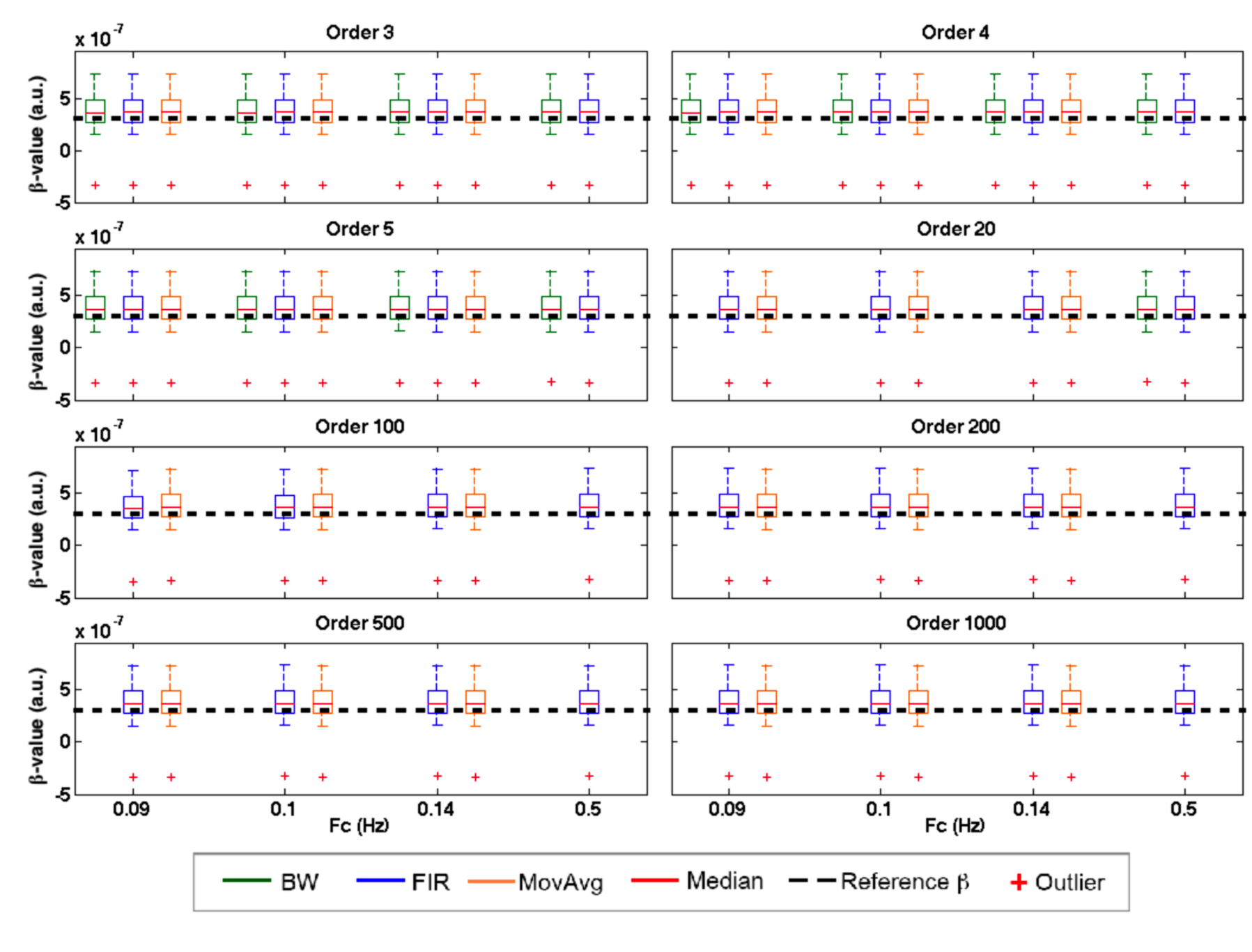
**

**Supplementary figure 35.** Boxplots referring to ΔHbO_2_^C^ LP filtered (green: BW; blue: FIR; orange: MovAvg) data, with Amplitude 3 and the precoloring method. Outliers are indicated as red crosses and can be observed in case of filters with low performance in signal denoising. The black dashed line represents the value of the reference *β*. Boxplots are not reported in case of unstable filters and for *F*_c_ = 0.5 Hz for the MovAvg filter that corresponds to a null window length.

**
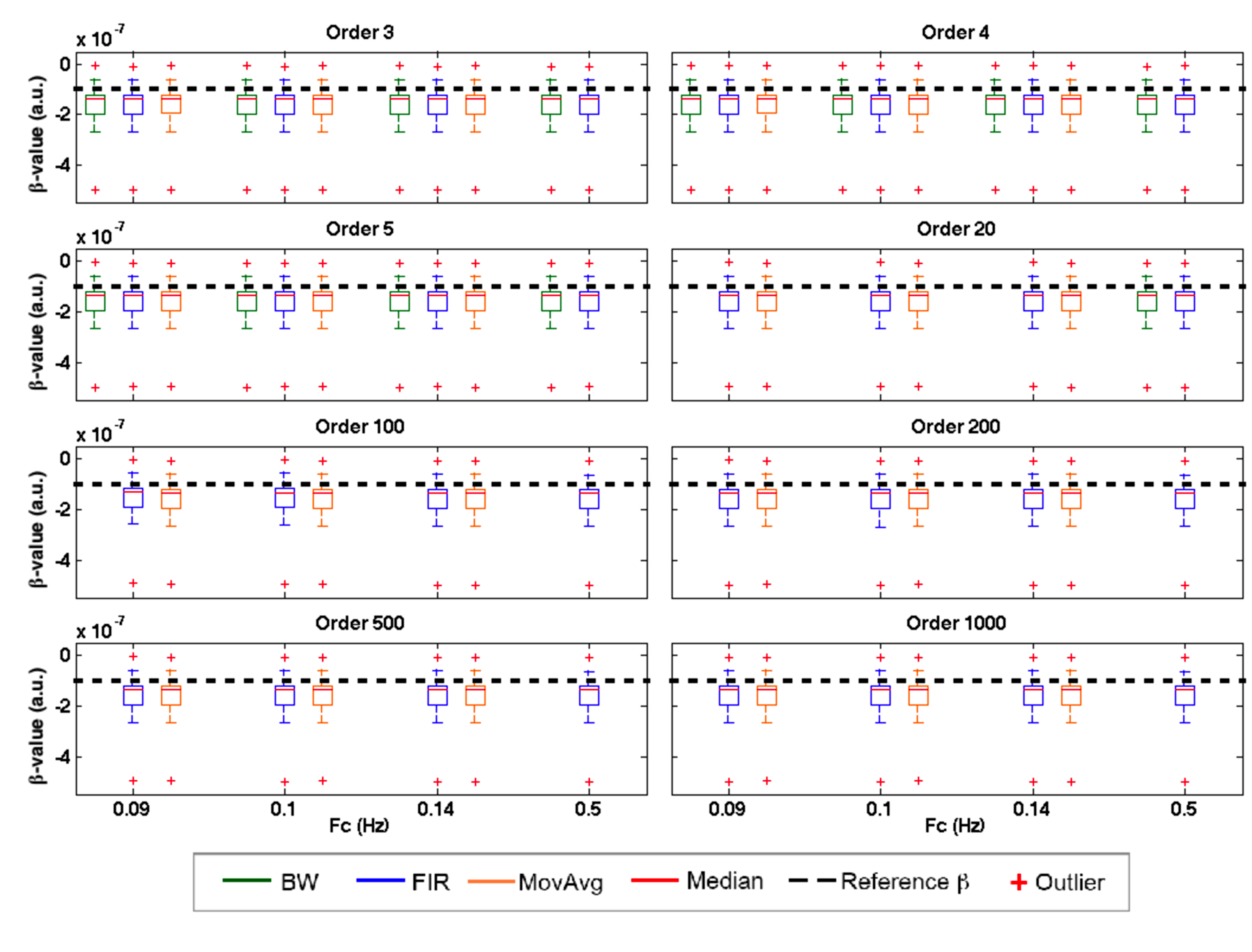
**

**Supplementary figure 36.** Boxplots referring to ΔHbR^C^ LP filtered (green: BW; blue: FIR; orange: MovAvg) data, with Amplitude 3 and the precoloring method. Outliers are indicated as red crosses and can be observed in case of filters with low performance in signal denoising. The black dashed line represents the value of the reference *β*. Boxplots are not reported in case of unstable filters and for *F*_c_ = 0.5 Hz for the MovAvg filter that corresponds to a null window length.
