# Supplementary material for: Current Status and Issues Regarding Pre-processing of fNIRS Neuroimaging Data: An Investigation of Diverse Signal Filtering Methods Within a General Linear Model Framework
Source: Front Hum Neurosci. 2019 Jan 11;12:505. doi: 10.3389/fnhum.2018.00505 (PMC6336925; doi:10.3389/fnhum.2018.00505)
Supplement: Supplementary file 4 [file Table_4.DOCX]

**List of reviewed studies**

Aasted, C. M., Yücel, M. A., Steele, S. C., Peng, K., Boas, D. A., Becerra, L., & Borsook, D. (2016). Frontal lobe hemodynamic responses to painful stimulation: a potential brain marker of nociception. *PLoS One*, *11*(11), e0165226.

Ahn, S., Nguyen, T., Jang, H., Kim, J. G., & Jun, S. C. (2016). Exploring neuro-physiological correlates of drivers' mental fatigue caused by sleep deprivation using simultaneous EEG, ECG, and fNIRS data. *Frontiers in human neuroscience*, *10*, 219.

Al-Shargie, F., Kiguchi, M., Badruddin, N., Dass, S. C., Hani, A. F. M., & Tang, T. B. (2016). Mental stress assessment using simultaneous measurement of EEG and fNIRS. *Biomedical optics express*, *7*(10), 3882-3898.

Aranyi, G., Pecune, F., Charles, F., Pelachaud, C., & Cavazza, M. (2016). Affective interaction with a virtual character through an fNIRS brain-computer interface. *Frontiers in computational neuroscience*, *10*, 70.

Balconi, M., & Vanutelli, M. E. (2016). Competition in the brain. The contribution of EEG and fNIRS modulation and personality effects in social ranking. *Frontiers in psychology*, *7*, 1587.

Balconi, M., & Vanutelli, M. E. (2016). Interbrains cooperation: hyperscanning and self-perception in joint actions. *Journal of clinical and experimental neuropsychology*, *39*(6), 607-620.

Balconi, M., & Vanutelli, M. E. (2016). Hemodynamic (fNIRS) and EEG (N200) correlates of emotional inter-species interactions modulated by visual and auditory stimulation. *Scientific reports*, *6*, 23083.

Baker, J. M., Liu, N., Cui, X., Vrticka, P., Saggar, M., Hosseini, S. H., & Reiss, A. L. (2016). Sex differences in neural and behavioral signatures of cooperation revealed by fNIRS hyperscanning. *Scientific reports*, *6*, 26492.

Bediz, C. S., Oniz, A., Guducu, C., Ural Demirci, E., Ogut, H., Gunay, E., ... & Ozgoren, M. (2016). Acute supramaximal exercise increases the brain oxygenation in relation to cognitive workload. *Frontiers in human neuroscience*, *10*, 174.

Cannizzaro, M. S., Stephens, S. R., Breidenstein, M., & Crovo, C. (2016). Prefrontal Cortical Activity During Discourse Processing. *Topics in Language Disorders*, *36*(1), 65-79.

Carius, D., Andrä, C., Clauß, M., Ragert, P., Bunk, M., & Mehnert, J. (2016). Hemodynamic response alteration as a function of task complexity and expertise—an fNIRS study in jugglers. *Frontiers in human neuroscience*, *10*, 126.

Carrieri, M., Petracca, A., Lancia, S., Basso Moro, S., Brigadoi, S., Spezialetti, M., ... & Quaresima, V. (2016). Prefrontal cortex activation upon a demanding virtual hand-controlled task: a new frontier for neuroergonomics. *Frontiers in human neuroscience*, *10*, 53.

Cavuoto, L. A., & Maikala, R. V. (2016). Obesity and the role of short duration submaximal work on cardiovascular and cerebral hemodynamics. *PloS one*, *11*(4), e0153826.

Chen, L. C., Sandmann, P., Thorne, J. D., Bleichner, M. G., & Debener, S. (2016 – Study 1). Cross-modal functional reorganization of visual and auditory cortex in adult cochlear implant users identified with fNIRS. *Neural plasticity*, *2016*.

Chen, L. C., Sandmann, P., Thorne, J. D., Bleichner, M. G., & Debener, S. (2016 – Study 2). Cross-modal functional reorganization of visual and auditory cortex in adult cochlear implant users identified with fNIRS. *Neural plasticity*, *2016*.

Chitnis, D., Airantzis, D., Highton, D., Williams, R., Phan, P., Giagka, V., ... & Elwell, C. E. (2016). Towards a wearable near infrared spectroscopic probe for monitoring concentrations of multiple chromophores in biological tissue in vivo. *Review of Scientific Instruments*, *87*(6), 065112.

Choe, J., Coffman, B. A., Bergstedt, D. T., Ziegler, M. D., & Phillips, M. E. (2016). Transcranial direct current stimulation modulates neuronal activity and learning in pilot training. *Frontiers in human neuroscience*, *10*, 34.

Cutini, S., Szűcs, D., Mead, N., Huss, M., & Goswami, U. (2016). Atypical right hemisphere response to slow temporal modulations in children with developmental dyslexia. *NeuroImage*, *143*, 40-49.

Davranche, K., Casini, L., Arnal, P. J., Rupp, T., Perrey, S., & Verges, S. (2016). Cognitive functions and cerebral oxygenation changes during acute and prolonged hypoxic exposure. *Physiology & behavior*, *164*, 189-197.

Ehlis, A. C., Haeussinger, F. B., Gastel, A., Fallgatter, A. J., & Plewnia, C. (2016). Task-dependent and polarity-specific effects of prefrontal transcranial direct current stimulation on cortical activation during word fluency. *Neuroimage*, *140*, 134-140.

Fernandez-Rojas, R., Huang, X., & Lopez-Aparicio, J.(2016) Nirs-based cortical activation analysis by temporal cross correlation. Signal & Image Processing: An International Journal (SIPIJ), 7, 3-41.

Foy, H. J., Runham, P., & Chapman, P. (2016). Prefrontal cortex activation and young driver behaviour: a fNIRS study. *PLoS one*, *11*(5), e0156512.

Fraser, S. A., Dupuy, O., Pouliot, P., Lesage, F., & Bherer, L. (2016). Comparable cerebral oxygenation patterns in younger and older adults during dual-task walking with increasing load. *Frontiers in aging neuroscience*, *8*, 240.

Fujimaki, K., Takemoto, H., & Morinobu, S. (2016). Cortical activation changes and sub-threshold affective symptoms are associated with social functioning in a non-clinical population: A multi-channel near-infrared spectroscopy study. *Psychiatry Research: Neuroimaging*, *248*, 73-82.

Fujita, H., Kasubuchi, K., Wakata, S., Hiyamizu, M., & Morioka, S. (2016). Role of the frontal cortex in standing postural sway tasks while dual-tasking: a functional near-infrared spectroscopy study examining working memory capacity. *BioMed research international*, *2016*.

Glassman, L. H., Forman, E. M., Herbert, J. D., Bradley, L. E., Foster, E. E., Izzetoglu, M., & Ruocco, A. C. (2016). The effects of a brief acceptance-based behavioral treatment versus traditional cognitive-behavioral treatment for public speaking anxiety: an exploratory trial examining differential effects on performance and neurophysiology. *Behavior modification*, *40*(5), 748-776.

Glassman, L. H., Kuster, A. T., Shaw, J. A., Forman, E. M., Izzetoglu, M., Matteucci, A., & Herbert, J. D. (2016). The relationship between dorsolateral prefrontal activation and speech performance-based social anxiety using functional near infrared spectroscopy. *Brain imaging and behavior*, *11*(3), 797-807.

Hancock, N. J., de Joux, N. R., Wingreen, S. C., Kemp, S., Thomas, J., & Helton, W. S. (2016). Positive post‐disaster images: A daydream machine?. *British Journal of Psychology*, *108*(3), 528-543.

Harrivel, A. R., Weissman, D. H., Noll, D. C., Huppert, T., & Peltier, S. J. (2016). Dynamic filtering improves attentional state prediction with fNIRS. *Biomedical optics express*, *7*(3), 979-1002.

Herrmann, M. J., Neueder, D., Troeller, A. K., & Schulz, S. M. (2016). Simultaneous recording of EEG and fNIRS during visuo-spatial and facial expression processing in a dual task paradigm. *International Journal of Psychophysiology*, *109*, 21-28.

Hernandez, M. E., Holtzer, R., Chaparro, G., Jean, K., Balto, J. M., Sandroff, B. M., ... & Motl, R. W. (2016). Brain activation changes during locomotion in middle-aged to older adults with multiple sclerosis. *Journal of the neurological sciences*, *370*, 277-283.

Higaki, N., Goto, T., & Ichikawa, T. (2016). Periodontal tactile input activates the prefrontal cortex. *Scientific reports*, *6*, 36893.

Holper, L. K., Aleksandrowicz, A., Müller, M., Ajdacic-Gross, V., Haker, H., Fallgatter, A. J., ... & Rössler, W. (2016). Distribution of Response Time, Cortical, and Cardiac Correlates during Emotional Interference in Persons with Subclinical Psychotic Symptoms. *Frontiers in behavioral neuroscience*, *10*, 172.

Hong, K. S., & Santosa, H. (2016). Decoding four different sound-categories in the auditory cortex using functional near-infrared spectroscopy. *Hearing Research*, *333*, 157-166.

Hong, K. S., & Naseer, N. (2016). Reduction of delay in detecting initial dips from functional near-infrared spectroscopy signals using vector-based phase analysis. *International journal of neural systems*, *26*(03), 1650012.

Huang, F., Hirano, D., Shi, Y., & Taniguchi, T. (2016). Comparison of cortical activation in an upper limb added-purpose task versus a single-purpose task: a near-infrared spectroscopy study. *Journal of physical therapy science*, *27*(12), 3891-3894.

Huhn, A. S., Meyer, R. E., Harris, J. D., Ayaz, H., Deneke, E., Stankoski, D. M., & Bunce, S. C. (2016). Evidence of anhedonia and differential reward processing in prefrontal cortex among post-withdrawal patients with prescription opiate dependence. *Brain research bulletin*, *123*, 102-109.

Hwang, H. J., Choi, H., Kim, J. Y., Chang, W. D., Kim, D. W., Kim, K., ... & Im, C. H. (2016). Toward more intuitive brain–computer interfacing: classification of binary covert intentions using functional near-infrared spectroscopy. *Journal of biomedical optics*, *21*(9), 091303.

Hyodo, K., Dan, I., Kyutoku, Y., Suwabe, K., Byun, K., Ochi, G., ... & Soya, H. (2016). The association between aerobic fitness and cognitive function in older men mediated by frontal lateralization. *Neuroimage*, *125*, 291-300.

Iso, N., Moriuchi, T., Sagari, A., Kitajima, E., Iso, F., Tanaka, K., ... & Higashi, T. (2016). Monitoring local regional hemodynamic signal changes during motor execution and motor imagery using near-infrared spectroscopy. *Frontiers in physiology*, *6*, 416.

Issa, M., Bisconti, S., Kovelman, I., Kileny, P., & Basura, G. J. (2016). Human auditory and adjacent nonauditory cerebral cortices are hypermetabolic in tinnitus as measured by functional near-infrared spectroscopy (fNIRS). *Neural plasticity*, *2016*.

Iwashiro, N., Koike, S., Satomura, Y., Suga, M., Nagai, T., Natsubori, T., ... & Yamasue, H. (2016). Association between impaired brain activity and volume at the sub-region of Broca's area in ultra-high risk and first-episode schizophrenia: A multi-modal neuroimaging study. *Schizophrenia research*, *172*(1-3), 9-15.

Jeong, E., & Ryu, H. (2016). Melodic Contour Identification Reflects the Cognitive Threshold of Aging. *Frontiers in aging neuroscience*, *8*, 134.

Jeong, E., & Ryu, H. (2016). Nonverbal auditory working memory: Can music indicate the capacity?. *Brain and cognition*, *105*, 9-21.

Jung, C. E., Strother, L., Feil-Seifer, D. J., & Hutsler, J. J. (2016). Atypical asymmetry for processing human and robot faces in autism revealed by fNIRS. *PloS one*, *11*(7), e0158804.

Kamiya, K., Narita, N., & Iwaki, S. (2016). Improved prefrontal activity and chewing performance as function of wearing denture in partially edentulous elderly individuals: functional near-infrared spectroscopy study. *PloS one*, *11*(6), e0158070.

Kashou, N. H., Giacherio, B. M., Nahhas, R. W., & Jadcherla, S. R. (2016). Hand-grasping and finger tapping induced similar functional near-infrared spectroscopy cortical responses. *Neurophotonics*, *3*(2), 025006.

Kempny, A. M., James, L., Yelden, K., Duport, S., Farmer, S., Playford, E. D., & Leff, A. P. (2016). Functional near infrared spectroscopy as a probe of brain function in people with prolonged disorders of consciousness. *NeuroImage: Clinical*, *12*, 312-319.

Kennedy, D. O., Stevenson, E. J., Jackson, P. A., Dunn, S., Wishart, K., Bieri, G., ... & Forster, J. (2016). Multivitamins and minerals modulate whole-body energy metabolism and cerebral blood-flow during cognitive task performance: a double-blind, randomised, placebo-controlled trial. *Nutrition & metabolism*, *13*(1), 11.

Kim, H. Y., Yang, S. P., Park, G. L., Kim, E. J., & You, J. S. H. (2016). Best facilitated cortical activation during different stepping, treadmill, and robot-assisted walking training paradigms and speeds: a functional near-infrared spectroscopy neuroimaging study. *NeuroRehabilitation*, *38*(2), 171-178.

Kohno, S., & Hoshi, Y. (2016). Spatial distributions of hemoglobin signals from superficial layers in the forehead during a verbal-fluency task. *Journal of biomedical optics*, *21*(6), 066009.

Koike, S., Satomura, Y., Kawasaki, S., Nishimura, Y., Takano, Y., Iwashiro, N., ... & Ichikawa, E. (2016). Association between rostral prefrontal cortical activity and functional outcome in first-episode psychosis: a longitudinal functional near-infrared spectroscopy study. *Schizophrenia research*, *170*(2-3), 304-310.

Li, L., Cazzell, M., Zeng, L., & Liu, H. (2016). Are there gender differences in young vs. aging brains under risk decision-making? An optical brain imaging study. *Brain imaging and behavior*, *11*(4), 1085-1098.

Lin, M. I. B., & Lin, K. H. (2016). Walking while performing working memory tasks changes the prefrontal cortex hemodynamic activations and gait kinematics. *Frontiers in Behavioral Neuroscience*, *10*, 92.

Liu, N., Mok, C., Witt, E. E., Pradhan, A. H., Chen, J. E., & Reiss, A. L. (2016). NIRS-based hyperscanning reveals inter-brain neural synchronization during cooperative Jenga game with face-to-face communication. *Frontiers in human neuroscience*, *10*, 82.

Liu, T., Liu, Y., He, W., He, W., Yu, X., Guo, S., & Zhang, G. (2016). A passenger reduces sleepy driver's activation in the right prefrontal cortex: a laboratory study using near-infrared spectroscopy. *Accident Analysis & Prevention*, *95*, 358-361.

Maidan, I., Nieuwhof, F., Bernad-Elazari, H., Reelick, M. F., Bloem, B. R., Giladi, N., ... & Mirelman, A. (2016). The role of the frontal lobe in complex walking among patients with Parkinson’s disease and healthy older adults: an fNIRS study. *Neurorehabilitation and neural repair*, *30*(10), 963-971.

Mahoney, J. R., Holtzer, R., Izzetoglu, M., Zemon, V., Verghese, J., & Allali, G. (2016). The role of prefrontal cortex during postural control in Parkinsonian syndromes a functional near-infrared spectroscopy study. *Brain research*, *1633*, 126-138.

McKendrick, R., Parasuraman, R., Murtza, R., Formwalt, A., Baccus, W., Paczynski, M., & Ayaz, H. (2016). Into the wild: neuroergonomic differentiation of hand-held and augmented reality wearable displays during outdoor navigation with functional near infrared spectroscopy. *Frontiers in human neuroscience*, *10*, 216.

Mehta, R. K. (2016). Stunted PFC activity during neuromuscular control under stress with obesity. *European journal of applied physiology*, *116*(2), 319-326.

Meng, S., Oi, M., Sekiyama, K., & Saito, H. (2016). The neural mechanism of biomechanical constraints in the hand laterality judgment task: A near-infrared spectroscopy study. *Neuroscience letters*, *627*, 211-215.

Metzger, F. G., Schopp, B., Haeussinger, F. B., Dehnen, K., Synofzik, M., Fallgatter, A. J., & Ehlis, A. C. (2016). Brain activation in frontotemporal and Alzheimer’s dementia: a functional near-infrared spectroscopy study. *Alzheimer's research & therapy*, *8*(1), 56.

Miura, N., Shirasawa, N., & Kanoh, S. I. (2016). Left lateral prefrontal activity reflects a change of behavioral tactics to cope with a given rule: An fNIRS study. *Frontiers in human neuroscience*, *10*, 558.

Moro, S. B., Carrieri, M., Avola, D., Brigadoi, S., Lancia, S., Petracca, A., ... & Quaresima, V. (2016). A novel semi-immersive virtual reality visuo-motor task activates ventrolateral prefrontal cortex: a functional near-infrared spectroscopy study. *Journal of neural engineering*, *13*(3), 036002.

Muthalib, M., Besson, P., Rothwell, J., Ward, T., & Perrey, S. (2016). Effects of anodal high-definition transcranial direct current stimulation on bilateral sensorimotor cortex activation during sequential finger movements: an fNIRS study. In *Oxygen Transport to Tissue XXXVII* (pp. 351-359). Springer, New York, NY.

Naseer, N., Noori, F. M., Qureshi, N. K., & Hong, K. S. (2016). Determining optimal feature-combination for LDA classification of functional near-infrared spectroscopy signals in brain-computer interface application. *Frontiers in human neuroscience*, *10*, 237.

Nieuwhof, F., Reelick, M. F., Maidan, I., Mirelman, A., Hausdorff, J. M., Rikkert, M. G. O., ... & Claassen, J. A. (2016). Measuring prefrontal cortical activity during dual task walking in patients with Parkinson’s disease: feasibility of using a new portable fNIRS device. *Pilot and feasibility studies*, *2*(1), 59.

Nishida, M., Kikuchi, S., Fukuda, K., & Kato, S. (2016). Jogging therapy for Hikikomori social withdrawal and increased cerebral hemodynamics: a case report. *Clinical practice and epidemiology in mental health: CP & EMH*, *12*, 38.

Nishiyori, R., Bisconti, S., & Ulrich, B. (2016). Motor cortex activity during functional motor skills: an fNIRS study. *Brain topography*, *29*(1), 42-55.

Nozawa, T., Sasaki, Y., Sakaki, K., Yokoyama, R., & Kawashima, R. (2016). Interpersonal frontopolar neural synchronization in group communication: an exploration toward fNIRS hyperscanning of natural interactions. *Neuroimage*, *133*, 484-497.

Olds, C., Pollonini, L., Abaya, H., Larky, J., Loy, M., Bortfeld, H., ... & Oghalai, J. S. (2016). Cortical activation patterns correlate with speech understanding after cochlear implantation. *Ear and hearing*, *37*(3), e160.

Osawa, T., Asano, H., Mizuno, T., Nozawa, A., Tanaka, H., Nomura, S., ... & Ide, H. (2016). Physiological and psychological evaluations in low-and high-frequency noise using near-infrared spectroscopy. *Artificial Life and Robotics*, *21*(1), 5-10.

Osofundiya, O., Benden, M. E., Dowdy, D., & Mehta, R. K. (2016). Obesity-specific neural cost of maintaining gait performance under complex conditions in community-dwelling older adults. *Clinical Biomechanics*, *35*, 42-48.

Pelowski, M., Oi, M., Liu, T., Meng, S., Saito, G., & Saito, H. (2016). Understand after like, viewer's delight? A fNIRS study of order-effect in combined hedonic and cognitive appraisal of art. *Acta psychologica*, *170*, 127-138.

Plenger, P., Krishnan, K., Cloud, M., Bosworth, C., Qualls, D., & de la Plata, C. M. (2016). fNIRS-based investigation of the Stroop task after TBI. *Brain imaging and behavior*, *10*(2), 357-366.

Pourshoghi, A., Zakeri, I., & Pourrezaei, K. (2016). Application of functional data analysis in classification and clustering of functional near-infrared spectroscopy signal in response to noxious stimuli. *Journal of Biomedical Optics*, *21*(10), 101411.

Propper, R. E., Dodd, K., Christman, S. D., & Brunyé, T. T. (2016). Relationship between sustained unilateral hand clench, emotional state, line bisection performance, and prefrontal cortical activity: A functional near-infrared spectroscopy study. *Laterality: Asymmetries of Body, Brain and Cognition*, *22*(6), 671-689.

Pu, S., Nakagome, K., Yamada, T., Itakura, M., Yamanashi, T., Yamada, S., ... & Iwata, M. (2016). Social cognition and prefrontal hemodynamic responses during a working memory task in schizophrenia. *Scientific reports*, *6*, 22500.

Pu, S., Nakagome, K., Itakura, M., Iwata, M., Nagata, I., & Kaneko, K. (2016). The association between cognitive deficits and prefrontal hemodynamic responses during performance of working memory task in patients with schizophrenia. *Schizophrenia research*, *172*(1-3), 114-122.

Rosner, A. O., & Barlow, S. M. (2016). Hemodynamic changes in cortical sensorimotor systems following hand and orofacial motor tasks and pulsed pneumotactile stimulation. *Somatosensory & motor research*, *33*(3-4), 145-155.

Sato, T., Nambu, I., Takeda, K., Aihara, T., Yamashita, O., Isogaya, Y., ... & Sato, M. A. (2016 – Study 1). Reduction of global interference of scalp-hemodynamics in functional near-infrared spectroscopy using short distance probes. *NeuroImage*, *141*, 120-132.

Sato, T., Nambu, I., Takeda, K., Aihara, T., Yamashita, O., Isogaya, Y., ... & Sato, M. A. (2016 – Study 2). Reduction of global interference of scalp-hemodynamics in functional near-infrared spectroscopy using short distance probes. *NeuroImage*, *141*, 120-132.

Shen, C. Y., Wang, Y. J., Zhang, X. Q., Liu, X. M., Ren, X. J., Ma, X. Y., ... & Liu, P. Z. (2016). Prefrontal Hemodynamic Functions during a Verbal Fluency Task in Blepharospasm Using Multi-Channel NIRS. *PloS one*, *11*(3), e0150804.

Shi, J., Zhou, W., Geng, T., Zuo, H., Tanida, M., & Sakatani, K. (2016). Effects of aging on working memory performance and prefrontal cortex activity: A time-resolved spectroscopy study. *临床转化神经科学*, *2*(1), 3-7.

Si, J., Zhang, X., Li, Y., Zhang, Y., Zuo, N., & Jiang, T. (2016). Correlation between electrical and hemodynamic responses during visual stimulation with graded contrasts. *Journal of Biomedical Optics*, *21*(9), 091315.

Singh, K., Bhargav, H., & Srinivasan, T. M. (2016). Effect of uninostril yoga breathing on brain hemodynamics: a functional near-infrared spectroscopy study. *International journal of yoga*, *9*(1), 12.

Stephens, J. A., & Berryhill, M. E. (2016). Older adults improve on everyday tasks after working memory training and neurostimulation. *Brain stimulation*, *9*(4), 553-559.

Sugai, M., & Adachi, M. (2016). Quantitative and qualitative discrimination of task periods from non-task periods by recurrence plots. *Nonlinear Theory and Its Applications, IEICE*, *7*(2), 283-294.

Tak, S., Uga, M., Flandin, G., Dan, I., & Penny, W. D. (2016). Sensor space group analysis for fNIRS data. *Journal of neuroscience methods*, *264*, 103-112.

Takeuchi, N., Mori, T., Suzukamo, Y., Tanaka, N., & Izumi, S. I. (2016). Parallel processing of cognitive and physical demands in left and right prefrontal cortices during smartphone use while walking. *BMC neuroscience*, *17*(1), 9.

Telles, S., Gupta, R. K., Singh, N., & Balkrishna, A. (2016). A functional near-infrared spectroscopy study of high-frequency yoga breathing compared to breath awareness. *Medical science monitor basic research*, *22*, 58.

Tempest, G. D., Eston, R. G., & Parfitt, G. (2016). A comparison of head motion and prefrontal haemodynamics during upright and recumbent cycling exercise. *Clinical physiology and functional imaging*, *37*(6), 723-729.

Tsunoda, K., Sekimoto, S., & Itoh, K. (2016). Near-infrared-spectroscopic study on processing of sounds in the brain; a comparison between native and non-native speakers of Japanese. *Acta oto-laryngologica*, *136*(6), 568-574.

Van de Rijt, L. P., van Opstal, A. J., Mylanus, E. A., Straatman, L. V., Hu, H. Y., Snik, A. F., & van Wanrooij, M. M. (2016). Temporal cortex activation to audiovisual speech in normal-hearing and cochlear implant users measured with functional near-infrared spectroscopy. *Frontiers in human neuroscience*, *10*, 48.

Vannasing, P., Cornaggia, I., Vanasse, C., Tremblay, J., Diadori, P., Perreault, S., ... & Gallagher, A. (2016). Potential brain language reorganization in a boy with refractory epilepsy; an fNIRS–EEG and fMRI comparison. *Epilepsy & behavior case reports*, *5*, 34-37.

Vasta, R., Cerasa, A., Gramigna, V., Augimeri, A., Olivadese, G., Pellegrino, G., ... & Grova, C. (2016). The movement time analyser task investigated with functional near infrared spectroscopy: an ecologic approach for measuring hemodynamic response in the motor system. *Aging clinical and experimental research*, *29*(2), 311-318.

Vega, R., Hernandez-Reynoso, A. G., Linn, E. K., Fuentes-Aguilar, R. Q., Sanchez-Ante, G., Santos-Garcia, A., & Garcia-Gonzalez, A. (2016). Hemodynamic pattern recognition during deception process using functional near-infrared spectroscopy. *Journal of medical and biological engineering*, *36*(1), 22-31.

Vermeij, A., Kessels, R. P., Heskamp, L., Simons, E. M., Dautzenberg, P. L., & Claassen, J. A. (2016). Prefrontal activation may predict working-memory training gain in normal aging and mild cognitive impairment. *Brain imaging and behavior*, *11*(1), 141-154.

Vrana, A., Meier, M. L., Hotz‐Boendermaker, S., Humphreys, B. K., & Scholkmann, F. (2016). Different mechanosensory stimulations of the lower back elicit specific changes in hemodynamics and oxygenation in cortical sensorimotor areas—A fNIRS study. *Brain and behavior*, *6*(12), e00575.

Ward, L. M., Morison, G., Simpson, W. A., Simmers, A. J., & Shahani, U. (2016). Using functional near infrared spectroscopy (fNIRS) to study dynamic stereoscopic depth perception. *Brain topography*, *29*(4), 515-523.

Watanabe, J. I., Atsumori, H., & Kiguchi, M. (2016). Informal face-to-face interaction improves mood state reflected in prefrontal cortex activity. *Frontiers in human neuroscience*, *10*, 194.

Watanuki, T., Matsuo, K., Egashira, K., Nakashima, M., Harada, K., Nakano, M., ... & Watanabe, Y. (2016). Precentral and inferior prefrontal hypoactivation during facial emotion recognition in patients with schizophrenia: A functional near-infrared spectroscopy study. *Schizophrenia research*, *170*(1), 109-114.

Wiggins, I. M., Anderson, C. A., Kitterick, P. T., & Hartley, D. E. (2016). Speech-evoked activation in adult temporal cortex measured using functional near-infrared spectroscopy (fNIRS): Are the measurements reliable?. *Hearing research*, *339*, 142-154.

Yamamuro, K., Kimoto, S., Iida, J., Kishimoto, N., Nakanishi, Y., Tanaka, S., ... & Kishimoto, T. (2016). Reduced prefrontal cortex hemodynamic response in adults with methamphetamine induced psychosis: relevance for impulsivity. *PloS one*, *11*(4), e0152373.

Yanagisawa, K., Nakamura, N., Tsunashima, H., & Narita, N. (2016). Proposal of auxiliary diagnosis index for autism spectrum disorder using near-infrared spectroscopy. *Neurophotonics*, *3*(3), 031413.

Yennu, A., Tian, F., Smith-Osborne, A., Gatchel, R. J., Woon, F. L., & Liu, H. (2016). Prefrontal responses to Stroop tasks in subjects with post-traumatic stress disorder assessed by functional near infrared spectroscopy. *Scientific reports*, *6*, 30157.

Yennu, A., Tian, F., Gatchel, R. J., & Liu, H. (2016). Prefrontal hemodynamic mapping by functional near-infrared spectroscopy in response to thermal stimulations over three body sites. *Neurophotonics*, *3*(4), 045008.

Yücel, M. A., Selb, J., Aasted, C. M., Lin, P. Y., Borsook, D., Becerra, L., & Boas, D. A. (2016). Mayer waves reduce the accuracy of estimated hemodynamic response functions in functional near-infrared spectroscopy. *Biomedical optics express*, *7*(8), 3078-3088.

Zafar, A., & Hong, K. S. (2016). Detection and classification of three-class initial dips from prefrontal cortex. *Biomedical optics express*, *8*(1), 367-383.

Zhang, J., Lin, X., Fu, G., Sai, L., Chen, H., Yang, J., ... & Yuan, Z. (2016). Mapping the small-world properties of brain networks in deception with functional near-infrared spectroscopy. *Scientific reports*, *6*, 25297.
